# Genomic analyses of pneumococci reveal a wide diversity of bacteriocins – including pneumocyclicin, a novel circular bacteriocin

**DOI:** 10.1186/s12864-015-1729-4

**Published:** 2015-07-28

**Authors:** Carlijn Bogaardt, Andries J van Tonder, Angela B Brueggemann

**Affiliations:** Nuffield Department of Medicine, Peter Medawar Building for Pathogen Research, University of Oxford, Oxford, United Kingdom

## Abstract

**Background:**

One of the most important global pathogens infecting all age groups is *Streptococcus pneumoniae* (the ‘pneumococcus’). Pneumococci reside in the paediatric nasopharynx, where they compete for space and resources, and one competition strategy is to produce a bacteriocin (antimicrobial peptide or protein) to attack other bacteria and an immunity protein to protect against self-destruction. We analysed a collection of 336 diverse pneumococcal genomes dating from 1916 onwards, identified bacteriocin cassettes, detailed their genetic composition and sequence diversity, and evaluated the data in the context of the pneumococcal population structure.

**Results:**

We found that all genomes maintained a *blp* bacteriocin cassette and we identified several novel *blp* cassettes and genes. The composition of the ‘bacteriocin/immunity region’ of the *blp* cassette was highly variable: one cassette possessed six bacteriocin genes and eight putative immunity genes, whereas another cassette had only one of each. Both widely-distributed and highly clonal *blp* cassettes were identified. Most surprisingly, one-third of pneumococcal genomes also possessed a cassette encoding a novel circular bacteriocin that we called pneumocyclicin, which shared a similar genetic organisation to well-characterised circular bacteriocin cassettes in other bacterial species. Pneumocyclicin cassettes were mainly of one genetic cluster and largely found among seven major pneumococcal clonal complexes.

**Conclusions:**

These detailed genomic analyses revealed a novel pneumocyclicin cassette and a wide variety of *blp* bacteriocin cassettes, suggesting that competition in the nasopharynx is a complex biological phenomenon.

**Electronic supplementary material:**

The online version of this article (doi:10.1186/s12864-015-1729-4) contains supplementary material, which is available to authorized users.

## Background

The pneumococcus is among the most important pathogens worldwide: in 2000, ~14.5 million estimated cases of life-threatening pneumococcal diseases like pneumonia, bacteraemia and meningitis occurred and ~826,000 children died [1]. Pneumococcal disease can be treated with antibiotics, but antibiotic-resistant pneumococci are found worldwide, e.g. 60 % of pneumococci recovered in Asia are multidrug-resistant [2]. Pneumococcal conjugate vaccines (PCVs) are administered to children in many developed countries and some resource-poor countries, which has significantly reduced morbidity and mortality [3]; however, current PCVs only protect against 10 or 13 pneumococcal types, depending on the vaccine. Pneumococcal types are defined by an antigenic polysaccharide capsule or ‘serotype’: over 90 different serotypes have been characterised and new ones continue to be discovered [4, 5]. Consequently, after PCV is introduced, vaccine-serotype disease decreases and nonvaccine-serotype disease often increases [6]. The prevalence of commensal pneumococci in the paediatric nasopharynx, its ecological niche, generally remains the same post-PCV, but reorders in favour of nonvaccine serotypes [7]. Vaccine escape is also possible and new variants can spread rapidly [8–10].

Nasopharyngeal colonisation of one or more pneumococcal serotypes is ubiquitous among children and usually asymptomatic [11]. The composition of colonising pneumococci fluctuates over time, indicating the importance of intraspecies competition in pneumococcal ecology [12, 13]. Understanding the dynamics of competition is important in the context of understanding how perturbations such as vaccine introduction affect the pneumococcal population structure and result in changes in the pneumococci competing for space and nutrients in the nasopharynx.

Bacteriocins are small, ribosomally-synthesised antimicrobial peptides or proteins produced by bacteria to inhibit other bacteria, and the producer strain has a dedicated immunity system that protects it from its own bacteriocin. Bacteriocins are a diverse group of compounds in terms of size, mode of action and immunity mechanisms, and are produced by both Gram-positive and Gram-negative bacteria. Given that the producer and target strains are often of the same bacterial species, the bacteriocins are largely believed to be involved in competition for limited resources within an ecological niche, but they may also be contributing to the maintenance of microbial diversity at a population level [14–16].

Pneumococci have been shown to mediate intraspecies competition by the production of bacteriocins encoded by the highly variable *blp* (also known as *spi/pnc*) cassette [17–22]. This cassette typically contains several bacteriocin-like peptide genes, clustered together with genes for putative membrane proteins that may have functions in immunity, in a region labelled the ‘bacteriocin/immunity region’ (BIR). The predicted pneumococcal bacteriocin peptides show homology to type II bacteriocin precursor peptides, and consist of a conserved N-terminal leader sequence ending in a double glycine motif and an alanine/glycine-rich mature peptide [17]. Previous studies have demonstrated the existence of genes for at least 10 such peptides within the *blp* cassette [17, 20–22].

The *blp* locus is regulated by quorum sensing, in a manner reminiscent of the regulation of competence by ComCDE [17, 18]. This is typically effected by a three-component system consisting of sensor histidine kinase BlpH (SpiH), response regulator BlpR (SpiR2), and peptide pheromone BlpC (SpiP). At high extracellular concentrations of BlpC the pheromone binds to BlpH, in turn activating BlpR [23]. BlpR then binds to conserved motifs in promoters, inducing expression of all genes in the locus, resulting in the production of more BlpC and bacteriocins. Similar to the bacteriocin-like peptides, BlpC has an N-terminal leader sequence [17, 18]; upon recognition by the dedicated ABC transporter BlpAB (SpiABCD), the signal peptide is cleaved off, and the mature peptide is transported out of the cell [24]. Sequence analyses and induction experiments showed that different BlpC sequences correspond to separate pherotypes, with no cross-induction of the *blp* genes [17, 18].

The aims of our study were to: i) provide a detailed characterisation and comparison of the *blp* bacteriocin cassettes from a large and diverse set of historical and modern pneumococcal genomes; ii) investigate cassette diversity in the context of the pneumococcal population structure; and iii) investigate the genetic stability of *blp* cassettes over time. We classified the *blp* bacteriocin cassettes based on gene content and nucleotide sequence, and revealed several novel cassette types. Surprisingly, we also discovered evidence for a circular bacteriocin cassette, which has not previously been described among pneumococci.

## Results

### Presence, nomenclature and classification of *blp* cassettes

All 336 of the pneumococcal genomes investigated (Additional file 1) had a *blp* bacteriocin cassette. In the literature there are two sets of gene names associated with these cassettes in pneumococci and we have identified additional novel genes in this study, adding to the confusion around nomenclature. Therefore, the gene names used in this study are presented in Table 1. Unresolvable ambiguities were encountered in seven cassette assemblies and these were excluded from further analyses. Sequences for the 329 remaining *blp* cassettes were separated by gene content into 33 Categories and by sequence similarity into 79 Groups. 79 Group prototype cassettes were analysed further.

**Table 1:** *blp* genes and predicted functions of their products

Gene	Synonym(s)	Predicted function of product^b^
*blpT*	--	**Hypothetical protein**
*blpS*	*spiR1*	**LytTR DNA-binding domain protein**
*blpR*	*spiR2*	**Response regulator** ^**c**^
*blpH*	*spiH*	**Histidine kinase** ^**c**^
*blpC*	*spiP*	**Peptide pheromone** ^**c**^
*blpB*	*spiD*	**Transport accessory protein**
*blpA*	*spiABC*	**ABC transporter** ^**c**^
*blpI*	*pncA*	Class II bacteriocin precursor^c^
*blpJ*	*pncD*	Class II bacteriocin precursor^c^
*blpK*	*pncE, pncE2*	Class II bacteriocin precursor^c^
*blpKN*	*pncU*	Class II bacteriocin precursor (hybrid)
*pncG*	--	Membrane protein (putative function in immunity)
*blpL*	*pncH*	Membrane protein (putative function in immunity)
*blpM*	*pncI*	Class II bacteriocin precursor^c^
*blpN*	*pncJ*	Class II bacteriocin precursor^c^
*blpMN1* ^*a*^	--	Class II bacteriocin precursor (hybrid)
*blpMN2* ^*a*^	--	Class II bacteriocin precursor (hybrid)
*blpP*	*pncK*	Membrane protein (function in immunity)^c^
*blpO*	*pncV*	Class II bacteriocin precursor
*pncM*	--	Membrane protein (putative function in immunity)
*blpQ*	*pncR*	Class II bacteriocin precursor
*blpQM* ^*a*^	--	Class II bacteriocin precursor (hybrid)
*pncT*	--	Class II bacteriocin precursor
*blpD* ^*a*^	--	Class II bacteriocin precursor
*blpE* ^*a*^	--	Class II bacteriocin precursor
*blpF* ^*a*^	--	Membrane protein (putative function in immunity)
*blpG* ^*a*^	--	CAAX protease (putative function in immunity)
*blpV* ^*a*^	--	Hypothetical protein
*blpW* ^*a*^	--	Class II bacteriocin precursor
*tdpA*	--	Thioredoxin domain containing protein
*pncW*	--	Class II bacteriocin precursor
*blpX*	*pncN*	Membrane protein (putative function in immunity)
*blpY*	*pncO*	**CAAX protease (function in immunity)** ^**c**^
*blpZ*	*pncQ*	**Membrane protein (putative function in immunity)**
*pncP*	SP0547	**CAAX protease (putative function in immunity)**

^a^Novel gene identified in this study.

^b^Genes with products marked with boldface font are found in all cassettes; the remaining genes are found in the bacteriocin/immunity region (BIR).

^c^The indicated function is supported by experimental evidence.

### Description of the *blp* cassettes at the level of gene organisation

**Fig. 1:**
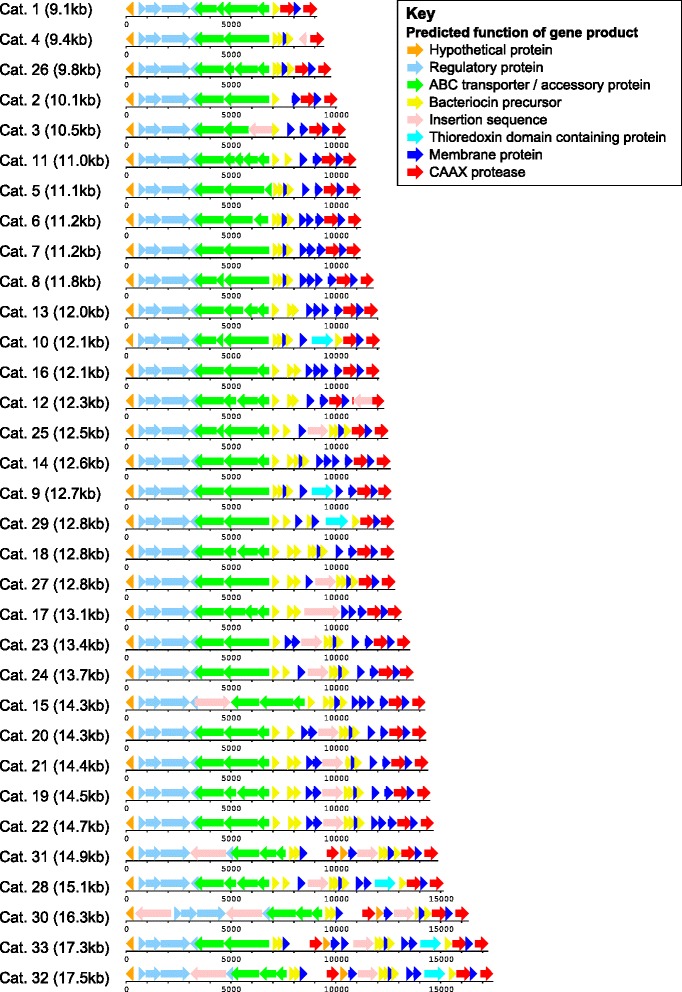
Schematic representation of the *blp* cassette Categories found in the pneumococcal genome collection. Genes are coloured according to the predicted functions of their products. Depicted cassettes are Group prototypes belonging to each Category. The organisation of the ABC transporter genes and the presence of insertion sequences are not representative of all cassettes in the labelled Category (see Methods section in main text)

The length of the *blp* bacteriocin gene cassettes ranged from 9.1 to 17.5 kb (Fig. 1). All Categories had a number of genes in common at the start and end of the cassette, including the regulatory, ABC transporter and CAAX protease genes (possibly related to bacteriocin self-immunity [25]), and a membrane protein gene putatively associated with immunity (Tables 1 and 2). One exception to this was that *blpY* and *blpZ* were missing in Category 4, having been replaced by the remnants of an insertion sequence (IS) element.

**Table 2:** Description of the 33 *blp* cassette Categories identified in 329 genomes

Cat^a^	Groups	Genomes (n)	Gene organisation^b, c, d^																																	Pherogroup (*blp* Group)^e^
1	a, b	3	T	S	R	H	C	B	A																							W*	Y	Z	P*	B (1a), A (1b)
2	--	1	T	S	R	H	C	B	A										**K**													X	Y	Z	P*	A
3	--	3	T	S	R	H	C	B	A																		**O**			M*		X	Y	Z	P*	D
4	--	1	T	S	R	H	C	B	A															**M**	**N**	P	**O**								P*	B
5	a, b	2	T	S	R	H	C	B	A															**M**	**N**	P	**O**			M*		X	Y	Z	P*	A (5a), H/M (5b)
6	--	8	T	S	R	H	C	B	A															**M**	**N**	P	**K**	G*	L			X	Y	Z	P*	A
7	a, b	3	T	S	R	H	C	B	A															**M**	**N**	P	**O**	G*	L			X	Y	Z	P*	B (7a), D (7b)
8	a - h	15	T	S	R	H	C	B	A															**M**	**N**	P	**O**	G*	L	M*		X	Y	Z	P*	C (8a, d-f), H/M (8bc), D (8 g), B (8 h)
9	--	1	T	S	R	H	C	B	A															**M**	**N**	P	**O**	G*		M*	*tdpA*	X	Y	Z	P*	A
10	a, b	2	T	S	R	H	C	B	A															**M**	**N**	P	**O**	G*			*tdpA*	W*	Y	Z	P*	D (10ab)
11	--	8	T	S	R	H	C	B	A								**I**										**O**			M*		X	Y	Z	P*	B
12	--	1	T	S	R	H	C	B	A								**I**	**J**									**O**			M*		X	Y	Z	P*	B
13	a, b	2	T	S	R	H	C	B	A								**I**	**J**									**O**	G*	L	M*		X	Y	Z	P*	A (13a), B (13b)
14	a - h	94	T	S	R	H	C	B	A								**I**	**J**	**KN**							P	**O**	G*	L	M*		X	Y	Z	P*	D (14ab), H/M (14 cd), B (14e-h)
15	a, b	4	T	S	R	H	C	B	A								**I**	**J**	**KN**							P	**K**	G*	L	M*		X	Y	Z	P*	A (15a), D (15b)
16	a, b	2	T	S	R	H	C	B	A								**I**	**J**	**K**	G*	L									M*		X	Y	Z	P*	A (16a), B (16b)
17	a - e	21	T	S	R	H	C	B	A								**I**	**J**	**K**	G*	L											X	Y	Z	P*	A (17ab), H/M (17 cd), B (17e)
18	--	1	T	S	R	H	C	B	A								**I**	**J**	**K**					**M**	**N**	P	**O**			M*		X	Y	Z	P*	D
19	a - m	41	T	S	R	H	C	B	A								**I**	**J**	**K**	G*	L			**M**	**N**	P	**O**			M*		X	Y	Z	P*	D (19ab, d, m), B (19c, l), C (19e), H/M (19f-j), A (19 k)
20	--	2	T	S	R	H	C	B	A								**I**	**J**		G*	L			**M**	**N**	P	**O**			M*		X	Y	Z	P*	C
21	--	3	T	S	R	H	C	B	A								**I**	**J**	**K**	G*	L			**MN1**		P	**O**			M*		X	Y	Z	P*	H/M
22	--	1	T	S	R	H	C	B	A								**I**	**J**	**K**	G*	L			**M**	**N**	P	**O**	G*	L			X	Y	Z	P*	D
23	a, b	26	T	S	R	H	C	B	A										**K**	G*	L			**M**	**N**	P	**O**			M*		X	Y	Z	P*	H/M (23a), A (23b)
24	a - f	27	T	S	R	H	C	B	A						**Q**	T*				G*				**M**	**N**	P	**O**			M*		X	Y	Z	P*	B (24a), H/M (24b, f), D (24 cd), C (24e)
25	a - d	33	T	S	R	H	C	B	A						**Q**	T*				G*				**M**	**N**	P						W*	Y	Z	P*	B (25ab), H/M (25c), C (25d)
26	--	1	T	S	R	H	C	B	A						**QM**										**N**	P						W*	Y	Z	P*	A
27	--	1	T	S	R	H	C	B	A								**I**	**J**	**K**	G*				**M**	**N**	P						W*	Y	Z	P*	C
28	--	1	T	S	R	H	C	B	A						**Q**	T*				G*				**M**	**N**	P	**O**	G*	L		*tdpA*	W*	Y	Z	P*	D
29	--	1	T	S	R	H	C	B	A						**Q**	T*				G*		**W**								M*	*tdpA*	W*	Y	Z	P*	C
30	--	16	T	S	R	H	C	B	A	**D**	**E**	F	G	V							L			**MN2**		P						W*	Y	Z	P*	A
31	--	2	T	S	R	H	C	B	A	**D**	**E**	F	G	V							L			**M**	**N**	P						W*	Y	Z	P*	A
32	--	1	T	S	R	H	C	B	A	**D**	**E**	F	G	V							L			**M**	**N**	P	**O**	G*	L		*tdpA*	W*	Y	Z	P*	A
33	--	1	T	S	R	H	C	B	A	**D**	**E**	F	G	V							L		M*	**M**	**N**	P	**O**	G*	L		*tdpA*	W*	Y	Z	P*	A

^a^Cat = Category

^b^Letters represent *blp* genes; those marked with an asterisk represent genes with only a *pnc* name.

^c^Letters in boldface font represent bacteriocin precursor genes; those underlined represent membrane protein genes (with a putative function in immunity).

^d^Insertion sequences are not shown in this table.

^e^Four pherogroups, A-D, plus a heterogeneous group, H/M, were identified among the *blp* cassettes. See Additional file 3 for further details.

The demarcation of the ABC transporter genes (*blpA*) and transport accessory protein gene (*blpB*) was highly variable even within Categories, and the frequent division of *blpA* into multiple ORFs was consistent with findings from previous studies [21, 22]. Son *et al.* analysed the sequences of the transporter genes and linked the frequent presence of frameshift-causing repeats and deletions in *blpA* to ‘cheater’ (immunity only, non-inhibitory) phenotypes.

Located between the common genes, the composition of the BIR was highly variable among Categories (Table 2). The number of BIR genes ranged from 1–15 and included structural bacteriocin genes, putative immunity genes, a CAAX protease and genes with products of unknown function.

Overall, most cassettes had one copy of each gene, although Categories 22 and 28 had two copies of *pncG* and Categories 22, 32 and 33 had two copies of *blpL*. In every case, the paralogues were separated by multiple genes and their sequences differed considerably. Furthermore, both *blpK* and a hybrid gene *blpKN* were present in Category 15. We found three additional hybrid *blp* bacteriocin genes, resulting from the contraction of *blpM* + *blpN* in Categories 21 and 30, and *blpQ* + *blpM* in Category 26. A deletion in Category 20 fused *blpJ* + *blpK*, but left the coding sequences in separate frames, thus resulting in a different ending for *blpJ* and the loss of function of *blpK*.

Interestingly, the BIR of Categories 30–33 contained genes predicted to encode two novel class II bacteriocin precursors (*blpD* and *blpE*), a novel putative immunity protein (*blpF*), a CAAX protease (*blpG*) and a hypothetical protein (*blpV*; Tables 1 and 2). *blpV* appeared to be a partial bacteriocin precursor gene: its amino acid sequence contained two GxxxG motifs, and a typical class II bacteriocin leader sequence was located directly upstream but in a different frame. Another novel bacteriocin precursor gene (*blpW*) was found in the BIR of Category 29. A search of the NCBI non-redundant protein database yielded no homologous sequences in other pneumococcal genomes, but orthologues were found in *Streptococcus pseudopneumoniae* [RefSeq:WP_001093252; 92 % identity] and *Streptococcus mitis* (GenBank:KEQ38555.1; 91 % identity).

Cassettes belonging to Categories 9, 10, 28, 29, 32 and 33 contained one or two ORFs that, when considered together as a single gene (*tdpA*), encoded a protein with a thioredoxin domain and a Gram-positive anchor domain. The sequence of the complete gene from Category 29 was found to be homologous to genes found in *S. pseudopneumoniae* and *Streptococcus mitis* (99 % and 94 % amino acid sequence identity, respectively). The products of these orthologues were labelled as putative bacteriocin transport accessory proteins [GenBank:EID24163; GenBank:EID31510] or transposases [RefSeq:WP_000744512; RefSeq:WP_000795769].

Finally, it was known that *blp* bacteriocin gene clusters often contain IS elements [17, 18, 21, 22] and we also found that 40 of the 79 prototypes contained one or more IS elements (49 in total), 34 of which were an ISSpn7/IS1381-like sequence located in non-coding sequences upstream of *blpM, blpMN1* or *blpMN2*, in all prototypes of the Categories 19–25, 27–28 and 30–33. The remaining IS elements were: ISSpn5 (n = 10); IS1515 (n = 3); and ISSpn8 (n = 2).

### Sequence diversity of predicted products of *blp* genes (excluding ABC transporter genes and *tdpA*)

The *blp* bacteriocin cassettes each had four regulatory genes: *blpS, blpR, blpH* and *blpC*. Based on amino acid sequences of the deduced products, 13–26 allelic variants were revealed for each protein, although each protein had 2–3 predominant alleles (Additional file 2). It was previously shown that allelic diversity of the peptide pheromone BlpC corresponds to distinct pherotypes; based upon the BlpC peptide sequences there was evidence for four distinctly different pherogroups A-D (Table 2), which corresponded to the 6A, R6, P164 and TIGR4 pherotypes plus minor variants, respectively, as detailed in Additional file 3 [17, 18, 22]. The diversity of BlpC was concentrated in the C-terminal half (the mature peptide), after cleavage by the ABC transporter. The BlpC pherogroups were associated with specific allelic versions of histidine kinase BlpH and response regulator BlpR. There was also a heterogeneous fifth group with mismatched BlpC/BlpH combinations and proteins encoded by sequence regions derived from multiple pherogroups.

Sixteen different putative *blp* bacteriocin precursor genes were identified among the cassettes: ten were described previously [17, 18, 21]; three were novel (*blpD*, *blpE*, *blpW*); and three were newly-identified hybrid genes (*blpMN1*, *blpMN2*, *blpQM*; Table 1). *blpQ*, *pncT* and *pncW* were classified as putative bacteriocin precursor genes due to the encoded peptides containing a typical leader sequence, although they lacked other salient features of bacteriocins, i.e. Ala/Gly-rich sequence and GxxxG motifs [17]. The gene *pncW* was previously described as a fusion gene with a mutated cleavage motif [21]; however, in our dataset there were multiple prototypes in which PncW had a presumably functional double-glycine motif and thus we included it among the putative bacteriocin precursors.

The number of *blp* bacteriocin precursor genes ranged from 1–6 per cassette (Table 2). Most had 1–2 predominant amino acid alleles, and some also had minor allelic variants (Additional file 2). The peptides BlpM and BlpN were previously shown to contribute to intraspecies competition in a murine model of colonisation, and a difference of five amino acids in bacteriocin BlpMN was sufficient to change the inhibitory properties of a strain in overlay assays. Further *in vitro* mutagenesis work indicated that the product of *blpP*, located directly downstream of *blpMN*, mediates immunity against this bacteriocin [20].

The number of genes encoding putative immunity proteins ranged from 1–7 per cassette (Table 2). Six such genes were found within the BIR, and common gene *blpZ* was located near the end of the bacteriocin cassette. Based on the amino acid sequences, between 2–20 alleles per protein were identified, although each putative immunity protein had 1–3 predominant alleles (Additional file 2).

Three genes in the bacteriocin cassettes coded for putative CAAX amino terminal proteases: *blpY* and *pncP*, located at the end of every cassette, and a novel gene (*blpG*) within the BIR of prototypes 30 – 33. Several major and minor alleles for BlpY and PncP*,* but only one BlpG allele, were identified. Roles in immunity against bacteriocins have been suggested for CAAX proteases: they may contribute to immunity by processing or degrading proteins, but their targets are unknown [17, 21, 25, 26]. *In vitro* site-directed mutagenesis indicated that CAAX protease BlpY is essential for immunity and bacteriocin activity [21].

Finally, two genes encoding hypothetical proteins were identified in the *blp* bacteriocin cassettes: *blpT* is the first gene present in all cassettes and most prototypes possessed one of two major amino acid alleles at this locus; and *blpV* was newly identified in this study in four prototypes, all of which had identical BlpV sequences (Additional file 2).

### Molecular epidemiology of the *blp* bacteriocin cassettes

Category 14 was the most prevalent and widely-distributed cassette: it was present in 94 pneumococci of 13 serotypes, recovered from 1939–2007 in 18 countries around the world (Table 3). These pneumococci were members of 15 different clonal complexes (CCs), seven of which are major pneumococcal CCs circulating globally (Table 4; [27]). *blp* cassettes in Category 14 were further divided into eight Groups, based on variation among ~600 nucleotides across the 12.6 Kb cassette. Variable nucleotide maps of the Group variation within Categories are contained in Additional file 4.

**Table 3:** Summary metadata for the *blp* bacteriocin cassettes

*blp* Category^a^	Genomes (n)	Years of isolation	Countries (n)	CCs (n)	Serotypes (n)	*blp* Groups (n)
14	94	1939-2007	18	15	13	8
19	41	1952-2006	8	11	11	13
25	33	1945-2004	8	11	8	4
24	27	1939-2006	4	7	10	6
23	26	1967-2006	8	4	4	2
17	21	1952-2008	6	4	5	5
8	15	1948-2006	5	9	7	8
15	4	2005-2007	1	2	2	2
1	3	1916-2003	2	2	3	2
3	3	1952-1963	1	2	2	0
7	3	1978-1999	2	2	2	2
5	2	1939, 1976	1	2	1	2
10	2	1962, 1988	2	2	2	2
13	2	1943, 1952	2	2	2	2
16	2	1989, 2004	2	2	2	2
30	16	1997-2006	2	1	2	0
6	8	1937-2006	6	1	2	0
11	8	1997-2004	3	1	1	0
21	3	1997-2004	2	1	1	0
31	2	1995, 1999	1	1	1	0
20	2	2006	1	1	1	0
Other^b^	12	1969-2007	1 ea	1 ea	1 ea	0

^a^ *blp* Categories are numbered.

^b^‘Other’ indicates *blp* Categories that were each only represented by a single pneumococcal genome.

**Table 4:** Distribution of each *blp* cassette Category among genomes of major pneumococcal clonal complexes (CCs)

	Number of genomes with *blp* cassettes of the different Categories, stratified by CC^b^:																						
CC^serotype a^	14	19	25	24	23	17	8	15	1	3	7	5	10	13	16	30	6	11	21	31	20	Other	Total
81^23F^	39																						39
1094^6A^		22																					22
236/271/320^19F/A^			1													16				2		1	20
124^14^		2	1			16																	19
15^14^					19																		19
None41^19A^	14																					1	15
199^19A^	12																						12
180^3^									1									8				1	10
113^18C^				9		1																	10
66^14/19F/9V^	8						2																10
439^23F^			9																				9
218^12F/7F^	8													1									9
156/162^9V/19A^		6				3																	9
191^7F^																	8						8
2090^6A/19A^				8																			8
90^6B^			7																				7
176^19A^	1				5	1																	7
385^6B^			6																				6
490^misc c^	2	1	1	1										1									6
247^4/19A^				5																			5
306^1^		4																					4
62^11A^							3								1								4
Other^d^	10	6	8	4	2	0	10	4	2	3	3	2	2	0	1	0	0	0	3	0	2	9	71
Total	94	41	33	27	26	21	15	4	3	3	3	2	2	2	2	16	8	8	3	2	2	12	329

^a^CC = clonal complex; superscript indicates the predominant serotype associated with that CC.

^b^ ‘Other’ indicates *blp* Categories that were each only represented by a single pneumococcal genome.

^c^Misc = miscellaneous: genomes in CC490 encoded serotypes 2, 10 F, 18 F, 22A, 6B or 6C.

^d^Each of the Other CCs contributed ≤3 isolates to the Other CCs total.

Category 19 was genetically the most diverse type of *blp* cassette: these cassettes were found in pneumococci of 11 serotypes isolated from 1952–2006 in eight countries, and the pneumococci were members of 11 CCs. 13 different Groups could be identified based on variation among ~1700 nucleotides across the 14.5 Kb cassette, and these Groups could be clustered into three major phylogenetic clusters based on the nucleotide sequences (Additional file 4).

In contrast, Categories 30, 6 and 11 were found in pneumococci isolated from several countries, but from a single CC associated with 1–2 serotypes, as shown in Tables 3 and 4. All eight examples of cassette Category 6 had nearly identical nucleotide sequences across the 11.2 Kb *blp* cassette, and they were found among CC191^7F/A^ pneumococci recovered over the last seven decades. Category 30 and 11 *blp* cassettes were also nearly identical among modern pneumococci, although genomes of older isolates of either CC236/271/320^19F/A^ or CC180^3^ were not available to assess whether stability among Categories 30 and 11 persists over longer time periods, and the same *blp* cassette sequences were not found among any of the historical pneumococci of other CCs. Table 4 also shows that CCs generally possessed a *blp* cassette of only one, or one predominant, Category. This was not true for serotype and *blp* Category: among the most prevalent serotypes each was associated with several *blp* Categories (Additional file 5).

Category 6 cassettes were genetically stable, but there were also Groups within Categories that appeared to be similarly stable over several decades and found in widely-circulating CCs. Three examples were: i) Group 14a, identified in 61 genomes from 1952–2007, all but two of which were in CC81^23F^ (n = 39), CC199^19A^ (n = 12) and CC66^14/19F/9V^ (n = 8); ii) Group 23a, found in 21 genomes dating from 1967–2006, all but two of which were CC15^14^; and iii) Group 24d, identified in 9 genomes from 1939–1999, all of which were CC113^18C^. Further details are provided in Additional files 6 and 7.

**Fig. 2:**
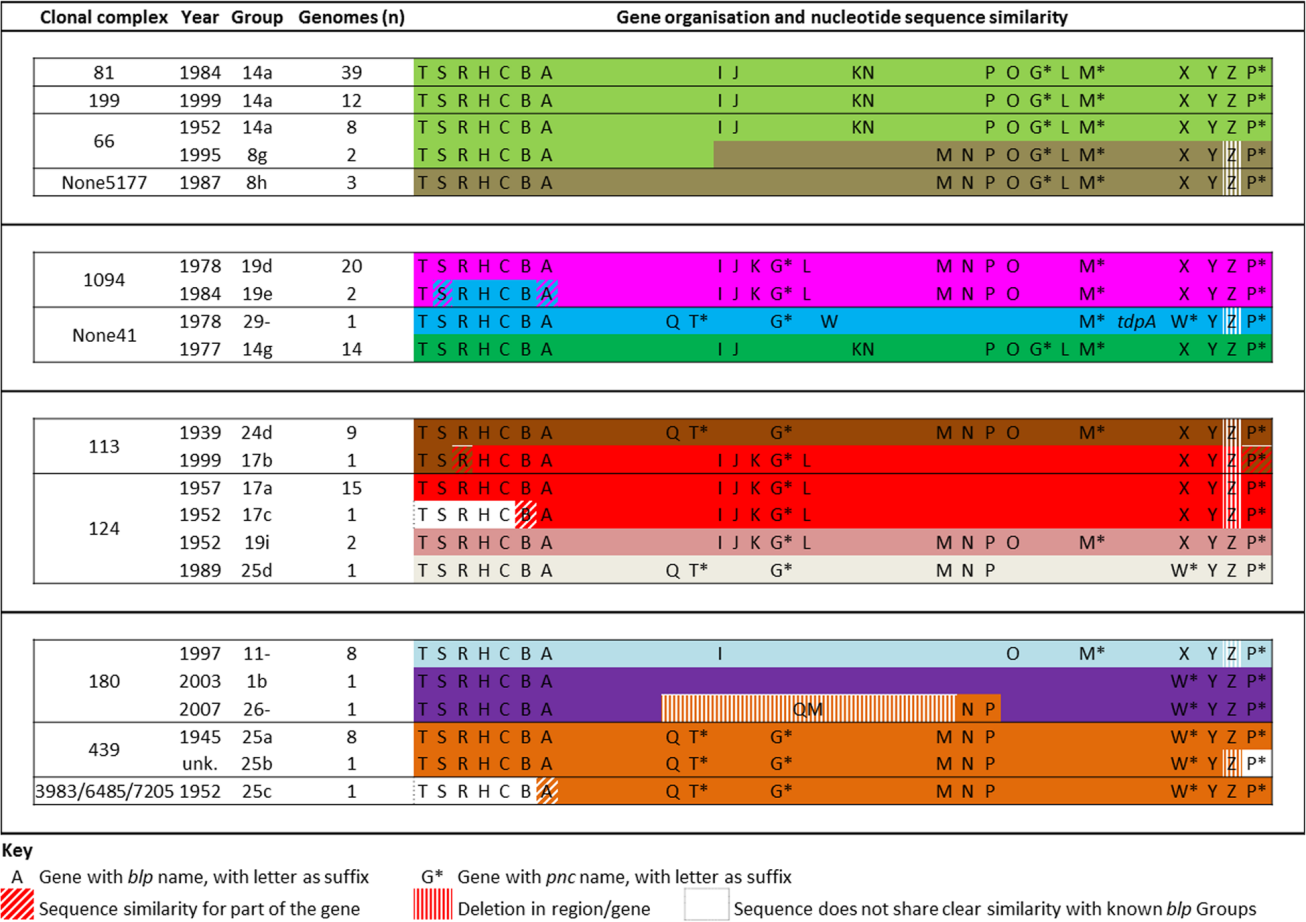
Examples of *blp* Groups and putative recombination blocks shared between different pneumococcal clonal complexes. Genes are coloured according to sequence similarity (or lack thereof), with matching colours indicating identical or highly similar sequences. Insertion sequences are not shown

Moreover, pneumococci are known to be recombinogenic and able to exchange large DNA fragments between unrelated pneumococcal lineages [8, 10, 28, 29], therefore we were interested in whether we could also identify evidence for regions of *blp* cassette sequence that were shared between unrelated CCs. The assembled prototype *blp* cassette sequences were thus aligned and inspected, and regions of identical or nearly identical sequence were indeed found between sequences of different prototypes. Several examples are shown in Fig. 2, which depicts shared *blp* cassette sequences between some of the major pneumococcal CCs, the pattern of which was consistent with evidence for recombination. Other examples of putative *blp* cassette recombination can be found in Additional file 8.

### Discovery of a novel pneumococcal circular bacteriocin (pneumocyclicin) cassette

During the investigation of the *blp* cassette, we identified a cluster of six genes located upstream of *comAB* in several strains: sequence analyses and BLAST searches suggested that this gene cluster, with a length of ~4.4 Kb, encodes the biosynthetic locus of a novel circular bacteriocin, which we provisionally named pneumocyclicin. This is the first report of circular bacteriocin cassettes among pneumococci, although they have been described in other Gram-positive species, including other *Streptococcus* spp, as explained below.

**Fig. 3:**
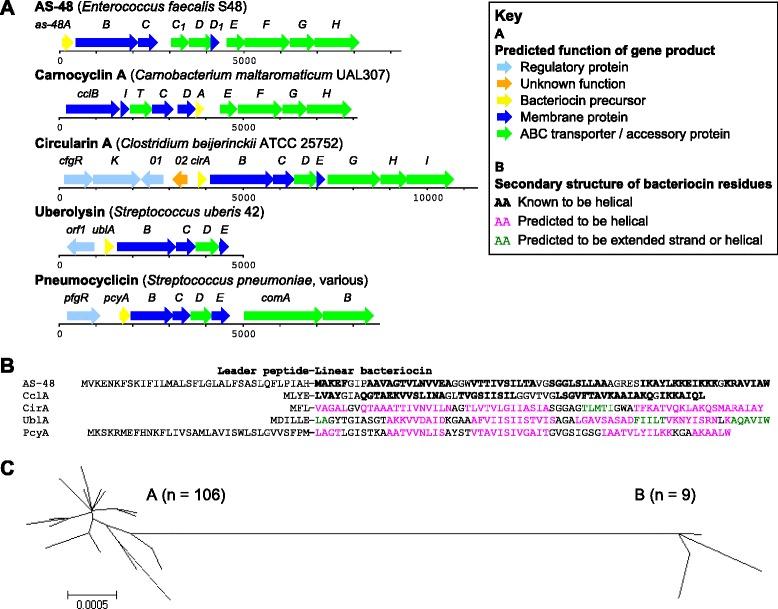
The pneumocyclicin cassette putatively encoding a newly-identified circular bacteriocin within the pneumococcal population. **a**. Comparison of the gene organisation of the pneumocyclicin cassette with those of class IIc(i) circular bacteriocins AS-48, carnocyclin A, circularin A and uberolysin. The genes *comA* and *comB*, adjacent to but not considered to be part of the pneumocyclicin cassette, were included to demonstrate that the synteny with other circular bacteriocin cassettes extends beyond the minimal cassettes and includes the downstream ABC transporter genes. **b**. Comparison of the pneumocyclicin precursor peptide sequence and predicted secondary structure with that of other class IIc(i) circular bacteriocins. Figure adapted from Martin-Visscher et al. [35]. **c**. Neighbour-joining tree demonstrating two distinct clusters of alleles representing the 115 pneumocyclicin cassettes in the study dataset

The pneumocyclicin genes were designated *pcyA-E* (*p*neumo*cy*clicin A-E) and *pfgR* (*pcyA*-*f*lanking *g*ene response *r*egulator). Predicted functions and physicochemical properties of their putative products are presented in Table 5. The gene organisation of the pneumocyclicin cassette was similar to that of previously identified circular bacteriocin cassettes [30–32], particularly that of uberolysin from *Streptococcus uberis* [33] and circularin A from *Clostridium beijerinckii* [34], as shown in Fig. 3a.

**Table 5:** Properties of pneumocyclicin genes and their deduced products

Gene		Deduced product		
Name	Length (bp)	Predicted function	Size (kDa)	pI^a^
*pfgR*	894	XRE family transcriptional regulator	34.5	4.9
*pcyA*	297	Circular bacteriocin (uberolysin/ circularin A family) precursor	10.1	10.4
*pcyB*	1137	Membrane protein (putative function in maturation/immunity)	44.3	9.6
*pcyC*	483	Membrane protein (putative function in maturation/immunity)	18.3	9.3
*pcyD*	597	ABC transporter ATP-binding protein	22.5	5
*pcyE*	492	Membrane protein (putative function in immunity)	18.9	9

^a^pI = predicted isoelectric point of the deduced protein.

The first gene in the pneumocyclicin cassette, *pfgR*, encodes a transcriptional regulator with an N-terminal helix-turn-helix XRE-family like domain. *pcyA* encodes the pneumocyclicin precursor, a 98-amino acid polypeptide from which pneumocyclicin could be derived by removal of an N-terminal leader sequence and circularisation of the remaining peptide [30–32]. Sequence analyses, a high isoelectric point (pI) and positively-charged residues indicated that pneumocyclicin belongs to the class IIc(i) circular bacteriocins, which have limited sequence similarity but share a common protein architecture consisting of four or five α-helices that form a saposin fold (Fig. 3b) [35].

The structural bacteriocin gene was followed by three genes encoding two putative membrane proteins (*pcyB* and *pcyC*) and one soluble ABC transporter ATP-binding protein (*pcyD*; Fig. 3a). The specific functions of PcyB and PcyC equivalents in other known cassettes are still unclear, but evidence points to roles in bacteriocin maturation and/or transport and immunity. PcyC, like CclC, As-48C, CirC and UblC, is a member of a protein family containing the domain of unknown function 95 (DUF95) [36]. The genes *as-48B* and *cirBCD* have been shown to be essential for production of AS-48 and circularin A, respectively, and *as-48C* and *cirBD* are required for full immunity to these bacteriocins [34, 37]. Finally, *pcyE* encodes another putative membrane protein. In the AS-48 and circularin A cassettes, the equivalents *as-48D*_*1*_ and *cirE* encode small hydrophobic peptides confirmed to be involved in immunity, but not sufficient for full resistance to these bacteriocins [34, 37]. However, the predicted PcyE protein was larger and contained more putative membrane spanning helices than either As-48D or CirE.

The cassettes for AS-48, carnocyclin A and circularin A each contain genes for a multicomponent ABC transporter downstream of the genes minimally required for bacteriocin production [34, 36, 38]. Although not essential, these genes were shown to enhance both production of and immunity to AS-48 [38]. Interestingly, the *comAB* operon located downstream of the *pcy* genes also encodes an ABC transporter, which is known to process and transport the competence-stimulating peptide CSP (Fig. 3a) [39].

### Prevalence and sequence diversity of the pneumocyclicin cassette

The pneumocyclicin cassette was present in 115 (34 %) of the 336 pneumococcal genomes in our dataset. Forty distinct nucleotide alleles described the 115 pneumocyclicin cassettes and they formed two sequence clusters, as shown in Fig. 3c. The overall nucleotide diversity was low, with a mean p-distance of 0.002 among all alleles and 0.001 within each sequence cluster. Sequence differences within and between clusters were concentrated within *pfgR* and the non-coding sequence between *pfgR* and *pcyA*. Four adjacent ATTT repeats were identified within *pcyB* (encoding one of the membrane proteins); several alleles from both clusters had ±1 copy of this repeat, which resulted in a frameshifted coding sequence. This is reminiscent of the four nucleotide repeat seen within *blpA* of the *blp* bacteriocin cassette and associated with the ‘cheater phenotype’ by Son *et al*. [22].

### Molecular epidemiology of the pneumocyclicin cassettes

Pneumocyclicin cassettes of both phylogenetic clusters were found in genomes dating from 1939 onwards. Cluster A alleles were by far the more prevalent of the two alleles, detected in 106 pneumococci recovered from 1939–2008 in 12 different countries. The pneumococci with cluster A alleles were members of 21 different CCs and were of 20 different serotypes. The nine cluster B alleles were found in pneumococci of seven different CCs and serotypes, and were recovered from 1939–2007.

Among the 115 genomes with a pneumocyclicin cassette, 80 % (n = 92) were members of one of seven CCs, six of which are major CCs circulating globally and CC1094 is a major South African CC (Table 6; [27]). Apart from one exception in a CC124 genome, all major CCs possessed cluster A pneumocyclicin alleles. Seven of these alleles were the most prevalent and together they represented 71 % (n = 75) of all 106 cluster A alleles. The sequences of these seven alleles were very similar, differing at only 12 nucleotides in total across the ~4.4 Kb pneumocyclicin cassette.

**Table 6:** Distribution of pneumocyclicin cassette alleles among pneumococcal clonal complexes (CCs)

	Clonal complex:	1094	124	199	66	113	156/162	439	Other CC^a^	Total (n)
	No. of genomes:	22	19	12	10	10	10	9	23	115
	Years of isolation:	1978-1988	1952-2005	1999-2005	1952-2007	1939-1999	1962-2008	1945-1996	1916-2007	1916-2008
**Cluster A alleles**	11		16						1	17
	26	14							1	15
	4			7				6	1	14
	2				10				0	10
	25	7							0	7
	1					7			0	7
	19						5		0	5
	Other^b^	1	2	5	0	3	5	3	12	31
	**Total A**	**22**	**18**	**12**	**10**	**10**	**10**	**9**	**15**	**106**
**Cluster B alleles**	36		1						2	3
	37								2	2
	40								1	1
	39								1	1
	35								1	1
	38								1	1
	**Total B**		**1**						**8**	**9**

^a^Each of the ‘Other’ CCs contributed ≤3 isolates to the Other CCs total

^b^Each of the ‘Other’ alleles was detected 1 or 2 times

## Discussion

Bacteriocins have generated renewed interest because of the major problems associated with antibiotic-resistant bacteria and the possible role of bacteriocins as alternatives to conventional antibiotics. There are many publications that describe the array of bacteriocins produced by many Gram-positive and Gram-negative bacteria. Comparatively less is known about pneumococcal bacteriocins, although several studies have delineated the genetic structure, function of some genes, and diversity of pneumococcal *blp* bacteriocin cassettes on a small number of pneumococcal strains, at a time when large scale sequencing was a challenge and limited genome data were available [17, 20–22]. In this study we found that *blp* bacteriocin cassettes were ubiquitous among a diverse set of pneumococcal genomes that dated back to 1916, in a variety of permutations with respect to both the genetic background of the host pneumococcus and the genetic composition of the *blp* bacteriocin cassette. Several novel genes and *blp* bacteriocin cassette types were also revealed.

Most surprisingly, we discovered that in addition to a *blp* bacteriocin cassette, a third of the pneumococcal genomes also possessed a cassette encoding a putative circular bacteriocin. To date, circular bacteriocins have predominantly been identified among the phylum Firmicutes (which include pneumococci) and are believed to be involved in niche competition. Circular bacteriocins in other Gram-positive bacterial species have been shown to permeabilise the bacterial cell membrane and cause cell death. They are ribosomally-synthesised and post-translationally modified to form a circular structure: as compared to a linear structure, the circular form is more stable, less susceptible to protease degradation and therefore more active. Circular bacteriocins potentially have a role in drug design, delivery and therapeutics, although many questions related to their structure, function and mechanisms of action remain to be determined [14, 31, 32].

The pneumocyclicin cassettes we discovered were similar in genetic structure and predicted proteins to circular bacteriocin cassettes characterised in other Gram-positive species. Nucleotide sequence similarity among the pneumocyclicin cassettes was high and the majority of cassettes were found in just seven pneumococcal CCs. It is curious that this cassette is just upstream of *comAB* – genes that have been shown to be essential to the development of competence, which is a specific point in the growth cycle during which a pneumococcus can take up and incorporate exogenous DNA into its genome [39, 40]. Recombination and transformation play a major role in the evolution of the pneumococcus; therefore, it will be crucial to understand not just the structure and function of pneumocyclicin but the potential impact that expression of pneumocyclicin genes may or may not have on competence induction.

Among *blp* bacteriocin cassettes, there was a wide repertoire of putative bacteriocin and immunity genes in the BIR region. Many individual cassettes possessed multiple putative bacteriocin genes and immunity genes: for example, Category 19 possessed six bacteriocin genes, six immunity genes and two CAAX protease genes, and genomes in six of the Category 19 Groups also possessed a cluster A pneumocyclicin cassette. Further work will be required to confirm the function of the genes in the BIR region and understand the biology that underpins the diversity of bacteriocin and immunity proteins. Does the possession of multiple bacteriocin and immunity genes simply mean the pneumococcus has an increased repertoire of bacteriocin arsenal and broadened immunity? A recent paper provided theoretical support for high bacteriocin diversity within a bacterial population and demonstrated that the maintenance of multiple bacteriocin and immunity types relied upon two key factors: circulating strains that were immune to the toxic effect of bacteriocins; and individual strains/lineages that were able to produce multiple bacteriocins and/or immunity proteins [41]. Is there a fitness cost associated with possession of multiple bacteriocin and immunity genes? Do some of these genes have alternative functions? Interestingly, a recent study demonstrated upregulation of *blp* bacteriocin genes in a pneumococcal infection animal model, possibly suggesting a role for some *blp* genes in pathogenesis and/or virulence [42].

We also evaluated the *blp* cassette diversity in the context of the pneumococcal population structure and found that some *blp* bacteriocins were found in many CCs whilst others seem to be restricted to one predominant CC. There was no obvious association between *blp* cassette type and serotype. Interestingly, some *blp* bacteriocin cassettes were genetically stable over many decades; although not surprisingly, patterns of putative large-fragment recombination similar to that previously reported in other recent pneumococcal studies were also identified [8, 10, 43].

Pneumococcal competition appears to be more complicated than just producing a bacteriocin peptide to kill competitors and an immunity protein to protect itself. Is the observed diversity of pneumococcal bacteriocins and/or the possession of multiple bacteriocin and immunity genes a reflection of a wider target specificity designed for nasopharyngeal competition? The paediatric nasopharynx is colonised by a variety of different microorganisms so it may be that the targets of the *blp* bacteriocins and pneumocyclicin are not solely pneumococci, but also viridans streptococci, *Haemophilus*, *Moraxella*, and others [13]. In previous work, Lux and colleagues investigated the *in vitro* inhibitory activity of pneumococci that produced bacteriocins using *Micrococcus luteus* and *Lactococcus lactis* as indicator strains, and they tested three *blp* bacteriocin-producing pneumococci against other oral streptococci and observed some inhibition [21]. Therefore, it is possible that the bacteriocins described here are predominantly mediating pneumococcal population-level interactions, but that they are also central to the interactions between pneumococci and other bacterial species residing in the nasopharynx.

Moreover, there is evidence that bacteria can engage in cooperative efforts within an ecological niche, often by means of quorum sensing whereby an individual detects and responds to an extracellular signal. However, this can result in ‘social cheaters’ – individuals who benefit from the cooperative efforts of the population without the fitness cost of exhibiting the specific traits themselves [15]. Son and colleagues demonstrated potential cheating behaviour among pneumococcal strains that produced immunity proteins but not the signalling pheromone or bacteriocins (due to frameshifted sequences), thereby avoiding costly bacteriocin production [22]. Intriguingly, we noticed that some alleles of *pcyB* in the pneumocyclicin cassette were frameshifted in a similar manner. Future studies will need to be designed to determine the intra- and interspecies activity of pneumococcal bacteriocins and the potential for a cheater phenotype among pneumococci with pneumocyclicin cassettes.

## Conclusions

Vast quantities of bacterial genome data have been generated in recent years and we used bioinformatics and computational biology tools to decipher the sequence-based evidence for bacteriocins, after which experimental studies can be designed to investigate specific hypotheses using carefully-selected candidate strains. One can look both forward and backward using these data: existing experimental evidence guides the interpretation of the genomic data, the design of new studies and the selection of test strains, but existing experimental data can also be reinterpreted based on genome sequence data. The sequence data are only predictive of function, but access to such comprehensive data is an efficient and cost-effective starting point to the challenging experimental work and should increase the likelihood of successful laboratory experiments.

## Methods

### Genome collection

The study dataset consisted of whole genome sequence data for 336 pneumococcal isolates recovered from 1916 to 2008 in 32 different countries (Additional file 1: Table S1): 206 published genomes [10, 43–46] and 130 GenBank genomes [47]. Ethical approval was not required to use any of the isolates in this study. Where not previously published or available online, serotype/group, multilocus sequence type and clonal complex were assigned as previously described [43].

### Classification of *blp* cassettes and prototype selection

Genomes were initially divided into groups based on approximate allelic profiles, as determined by a combination of two BIGSdb Genome Comparator [48] analyses, using as references the genes *blpS* through to *pncP* from the TIGR4 genome, and *blpQ, pncT* and *pncW* from the 2306 genome [21]. After sequence assembly, cassettes were divided into ‘Categories’ on the basis of gene presence and synteny, and into ‘Groups’ on the basis of sequence similarity, ignoring the presence of IS elements. When Categories were assigned, predictions of functionality were not taken into account.

Based on sequence alignments, separate Groups were created for sequences that differed by >15 nucleotide substitutions. Where the presence of an indel changed gene organisation separate Categories were assigned. A Group prototype was chosen based upon two criteria: i) fully sequenced, assembled and gap-free cassette (although gaps within IS elements were allowed); and ii) the cassette sequence from the oldest pneumococcus.

### Identification and assembly of *blp* cassette sequences

The *blp* cassette was defined as the genes between and including *blpT* and *pncP*. Completeness of the assemblies was checked by comparing automated gene predictions to expected gene presence based on the allelic profile, and by interrogating each genome for all known *blp* genes and those newly identified in this study. We also performed a second round of assemblies, this time using high throughput sequencing reads and their quality scores as coded in fastq files. Selected cassettes were re-assembled by mapping of the Illumina reads to the original Velvet contigs with SMALT, followed by inspection, correction, and manual joining of relevant contigs in Gap5 [49, 50]. IS elements within *blp* cassettes were not assembled but identified by their end sequences and left as gaps in the sequence database. After extraction of the cassette sequences into fasta files, the 5’ and 3’ ends of each IS element were trimmed to 25 bp and joined by Ns, so as to match the length listed for the specific IS element by ISfinder [51].

### Gene prediction and annotation

Genes were predicted with Prokka [52], Artemis was used for sequence visualisation and manual editing of annotations [53], and RATT was used to transfer annotations between genomes with similar cassettes [54]. The coding sequence (CDS) features predicted by Prokka were modified in some cases: i) *blpO* and *pncM*, inconsistently predicted, were manually annotated; ii) *blpP*, not predicted in any genomes, was manually annotated where its sequence was present because of prior experimental evidence for a role in immunity [20]; iii) bacteriocin CDSs with multiple putative start codons close together were adapted to start at M(D/N)T; iv) CDSs related to IS elements were replaced by mobile element features; and v) frameshifted genes were labelled, except within the transport region where CDSs were ambiguously organised and thus left as originally predicted by Prokka. Functional annotation was based on previously published literature, BLAST searches of non-redundant databases, and interrogation of the BAGEL3 [55] and BACTIBASE databases [56].Sequence and annotation files for *blp *cassette prototypes are found in Additional file 9.

### Identification and annotation of pneumocyclicin cassettes

The pneumocyclicin (*pcy*) cassette was defined as the genes between and including *pfgR* and *pcyE* and identified as described above. The cassettes were separated into nucleotide alleles using the NRDB tool [57] and assigned to phylogenetic clusters with MEGA5 [58]. Gene prediction and annotation with Prokka was complemented with manual BLAST searches and analysis through BAGEL3 to confirm its nature as a bacteriocin cassette.Sequence and annotation files for all *pcy *alleles are found in Additional file 10.

### Software used for sequence analyses and visualisation

BIGSdb [48] was used to store and query assembled *blp* cassette prototype sequences and associated metadata. BLAST searches were performed in BIGSdb or using BioEdit [59]. Sequence alignments and phylogenetic analyses were performed with MEGA5 and progressiveMauve [60]. Bacteriocin cassettes were visualised with DNAPlotter [61] and Inkscape [62].

### Availability of supporting data

The data sets supporting the results of this article are included within the article and its additional files.

## Additional files

Additional file 1: Table S1. Metadata for the 336 genomes included in this study. Additional file 2:
**Alignment S1.** Allelic variants of *blp* cassette gene products, by predicted function. Additional file 3:
**Alignment S2.** BlpR, BlpH and BlpC allele combinations (variable residues only), ordered by pherogroup. Additional file 4:
**Alignment S3.** Nucleotide sequence differences between *blp* Groups within each Category. Additional file 5: Table S2.
*blp* bacteriocin Categories stratified by serotype, and clonal complex (CC) within each serotype. Additional file 6: Table S3.
*blp* Groups stratified by pneumococcal year of isolation. Additional file 7: Table S4.
*blp* bacteriocin Groups stratified by clonal complex (CC). Additional file 8: Table S5. Clonal complexes (CCs) with multiple *blp* Groups, with regions with significant sequence differences highlighted. Additional file 9:
**Sequence and annotation files for** ***blp*** **cassette prototypes.**
Additional file 10:
**Sequence and annotation files for all** ***pcy*** **alleles.**

## Abbreviations

BIR: Bacteriocin/immunity region
CCs: Clonal complexes
CDS: Coding sequence
PCVs: Pneumococcal conjugate vaccines
*Pcy*: Pneumocyclicin
