# Supplementary material for: Genomic analyses of pneumococci reveal a wide diversity of bacteriocins – including pneumocyclicin, a novel circular bacteriocin
Source: BMC Genomics. 2015 Jul 28;16(1):554. doi: 10.1186/s12864-015-1729-4 (PMC4517551; doi:10.1186/s12864-015-1729-4)
Supplement: Additional file 2: — Alignment S1. Allelic variants of blp cassette gene products, by predicted function. [file 12864_2015_1729_MOESM2_ESM.docx]

**Alignment S1. Allelic variants of *blp* cassette gene products, by predicted function.**

The alignments do not include the products of ABC transporter genes *blpA* and *blpB*, or the thioredoxin domain containing protein gene *tdpA*: in many prototypes, these were split up into multiple open reading frames in various coding frames (see Additional file 3), with inconsistent starts and ends, complicating alignment of their products.

Within the alignments, alleles are ordered first by their length and secondly by their frequency (Freq.) among 79 prototypes.

Identical residues are represented by a dot (.), absent residues by a dash (-), and stop codons by an asterisk (*). Numbers at the end of each row represent residue positions.

Residues derived from insertion sequence (IS) element insertions are indicated in pink text.

For the peptide pheromone BlpC and the bacteriocin precursor peptides, leader sequences are indicated in blue text, with the double-glycine cleavage motif underlined.

**A) Regulatory proteins**

**BlpS**

**Freq. Peptide sequence**

17 MKYMIIQTQK TVYKVNIDDI YYIQTHPTKA HTVQIVTEEA SFNMLQNLSN LENQCGETLM RCHRNCLVNL DKLKSIDFQE RILFLGEEGQ YAVKYARRRY REIRQKWLKE GE* [113]

11 ..H....... .......... .......... ........K. .......... .......... .......... .......... .......... .......... .......... ... [113]

8 ..H....... ......V..V .......... ...K...... ....V..... ....Y..... .......... ..V....... .......... .......... .......... ... [113]

6 ..H....... .......... .......... ........K. .......... .......... .......... .......... .......... ...R...... .......... ... [113]

5 ..H....... ......V... .......... .......... .......... ....Y..... .......... .......... .......... .......... .......... ... [113]

4 ..H....... .......... .......... ........K. ....V..... .......... .......... .......... .......... .......... .......... ... [113]

3 .......... .......... .......... .......... .......... .......... .......... .......... .......... .......... .........Q ... [113]

3 ..H....... .......... .......... .......... .......... .......... .......... .......... .......... .......... .......... ... [113]

3 ..H....... ......V... .......... .......... .......... .......... .......... .......... .......... .......... .........Q ... [113]

3 ..H....... ......V... .......... ........K. .......... .......... .......... .......... .......... .......... .......... ... [113]

2 ..H....... ......V... .......... .......... .......... .......... .......... .......... .......... .......... .......... ... [113]

2 .......... .......... .......... .......... .......... .......... .......... .......... .......... .......... ........E. ... [113]

1 .......... ......V... .......... .......... .......... ....Y..... .......... .......... .......... .......... .......... ... [113]

1 ..H....... .......... .......... ........K. .......... ....Y..... .......... ..V....... .......... .......... .......... ... [113]

1 ..H....... .......... .......... ......I.K. .......... .......... .......... .......... .......... .......... .......... ... [113]

1 .......... .......... .......... .......... .....K.... .......... .......... .......... .......... .......... .........Q ... [113]

1 .......... ......V... .......... ........K. .......... .......... .......... .......... .......... .......... .......... ... [113]

1 .......... ......V... .......... ........K. .......... .......... .......... ..V....... .......... .......... .......... ... [113]

1 ..H....... .......... .......... ........K. .......... .......... .......... ..V....... .......... .......... .......... ... [113]

1 .......... .......... .......... .......... ...I...... .......... .......... .......... .......... .......... .......... ... [113]

1 ..H....... A......... .......... ........K. .......... .......... .......... .......... .......... .......... .........Q ... [113]

1 ..H....... ......V... .......... .......... .......... ....Y..... .......... .......... .......... .......... .........Q ... [113]

1 ..H....... ......V... .........S KHI.LKPIPY RLLQKKLVLI CFKI*----- ---------- ---------- ---------- ---------- ---------- --- [113]

1 ..H....... ......V... .......... IPYRLLQKKL VLICFKI*-- ---------- ---------- ---------- ---------- ---------- ---------- --- [113]

**BlpR**

**Freq. Peptide sequence**

14 MRIFVLEDDF SQQTRIETTI EKLLKAHHII PSSFEVFGKP DQLLAEVHEK GAHQLFFLDI EIRNEEMKGL EVARKIRDRD PYALIVFVTT HSEFMPLSFR YQVSALDYID KALSAEEFES RIETALLYAN [130]

10 .......... .......... .......... .......... .......... .......... .......... .......EQ. .......... .......... .......... .......... .......... [130]

7 .......... .......... .....E...T .......... .......... .......... .......... .......... .......... .......... .......... .......... .......... [130]

6 .......... .......... .......... .......... .......... .......... .......... .......... .......... .......... .......... .......... .......... [130]

6 .......... .......... .......... .......... .......... .......... .......... .......... .......... .......... .......... .......... .......... [130]

5 .......... .......... .....E...T .......... .......... .......... .......... .......EQ. .......... .......... .......... .......... .......... [130]

4 .......... .......... .....E...T L......... .......... .......... .......... .......... .......... .......... .......... .......... .......... [130]

4 .......... .......... .....E...T L......... .......... .......... .......... .......... .......... .......... .......... .......... .......... [130]

3 .......... .......... .....E...T .......... .......... .......... .......... .......EQ. .......... .......... .......... .......... .......... [130]

2 .......... .......... .....E...T .......... .......... .......... .......... .......... .......... .......... .......... .......... .......... [130]

2 .......... .......... .......... .......... .......... .......... .......... .......EQ. .......... .......... .......... .......... .......... [130]

2 .......... .......... .....E...T .......... .......... .......... .......... .......EQ. .......... .......... .......... ....T..... .......... [130]

1 .......... .......... .......... .......... .......... .D........ .......... .......... .......... .......... .......... .......... .......... [130]

1 .......... .......... .......... .......... .......... .D........ .......... .......... .......... .......... .......... .......... .......... [130]

1 .......... .......... .......... .......... ........K. .......... D......... .......... .......... .......... .......... .......... .......... [130]

1 .......... .......... .......... .......... .......... .......... .......... .......... .......... .......... .......... .......... .......... [130]

1 .......... .......... .....E...T L......... .......... .......... .......... .......EQ. .......... .......... .......... .......... .......... [130]

1 .......... .......... .......... .......... .......... .......... .......... .......EQ. .......... .......... .......... .......... .......... [130]

1 .......... .......... .......... .......... .......... .......... .......... .......... .......... .......... .......... .......... .......... [130]

1 .......... .......... .....E...T L......... .......... .......... .......... .......... .......... .......... .......... .......... .......... [130]

1 .......... .......... .......... .......... ........K. .......... .......... .......... .......... .......... .......... .......... .......... [130]

1 .......... .......... .......... .......... .......... ...R...... .......... .......... .......... .......... .......... .......... .......... [130]

1 .......... .......... .......... .......... .......... .......... .......... .......... .......... .......... .......... .......... .......... [130]

1 .......... .......... .......... .......... .......... .......... .......... .......... .......... .......... .......... .......... .......... [130]

1 .......... .......... .......... .......... .......... .......... .......... .......... .......... .......... .......... .......... ....D..... [130]

1 .......... .......... .....E...T .......... .......... ..ISYS.WIL RFEMRR*--- ---------- ---------- ---------- ---------- ---------- ---------- [130]

14 SQDSKSLAED CFYFKSKFAQ FQYPFKEVYY LETSPRAHRV ILYTKTDRLE FTASLEEVFK QEPRLLQCHR SFLINPANVV HLDKKEKLLF FPNGGSCLIA RYKVREVSEA INKLH* [246]

10 .......... .......... .......... ......P... .......... .......... .......... .......... .......... .......... .......... ..N... [246]

7 .......... .......... .......... ......P... .......... .......... .......... .......... .......... .......... .......... ..N... [246]

6 .......... .......... .......... .......... .......... .......... .......... .......... .......... .......... .......... ..N... [246]

6 .......... .......... .......... ......P... .......... .......... .......... .......... .......... .......... .......... ..N... [246]

5 .......... .......... .......... ......P... .......... .......... .......... .......... .......... .......... .......... ..N... [246]

4 G......... .......... .......... ....T.P... .......... ........L. ....F..... .......... .......... .......... .......... ..N... [246]

4 G......... .......... .......... ....T.P... .......... ........L. .......... .......... .......... .......... .......... ..N... [246]

3 .......... .......... .......... ....T.P... .......... ........L. .......... .......... .......... .......... .......... ..N... [246]

2 G......... .......... .......... ....T.P... .......... ........L. .......... .......... .......... .......... .......... ..N... [246]

2 G......... .......... .......... ....T.P... .......... ........L. .......... .......... .......... .......... .......... ..N... [246]

2 .......... .......... .......... ......P... .......... .......... .......... .......... .......... .......... .......... ..N... [246]

1 .......... .......... .......... .......... .......... .......... .......... .......... .......... .......... .......... ..N... [246]

1 .......... .......... .......... .......... .......... .......... .......... .......... .......... .......... .......... ...... [246]

1 .......... .......... .......... .......... .......... .......... .......... .......... .......... .......... .......... ...... [246]

1 .......... .......... .......... .......... .......... ........L. .......... .......... .......... .......... .......... ..N... [246]

1 .......... .......... .......... ......P... .......... .......... .......... .......... .......... .......... .......... ..N... [246]

1 .......... .......... .......... ......P... .......... .......... ...S...... .......... .......... .......... .......... ..N... [246]

1 G......... .......... .......... ....T.P... .......... ........L. .......... .......... .......... .......... .......... ..N... [246]

1 .......... .......... .......... .......... .......... .......... .......... .......... .......... .......... .......... ...... [246]

1 .......... .......... .......... .......... .......... .......... .......... .......... .......... .......... .......... ...... [246]

1 .......... .......... .......... .......... .......... .......... .......... ......V... .......... .......... .......... ...... [246]

1 .......... .......... .......... ....T.P... .......... ........L. .......... .......... .......... .......... .......... ..N... [246]

1 .......... .......... .......... ......T... .......... .......... .......... .......... .......... .......... .......... ...... [246]

1 .......... .......... .......... .......... .......... .......... .......... .......... .......... .......... .......... ..N... [246]

1 ---------- ---------- ---------- ---------- ---------- ---------- ---------- ---------- ---------- ---------- ---------- ------ [246]

**BlpH**

**Freq. Peptide sequence**

13 MNIAWILLYA LVINGLEIVI FFKVDGIGLT FDRIFKAFLL KFLLGIIFTT FQFLAVSKYL SYFIEPLFGI GLSFLLLRGL PKKILIFYGL FPMILVELFY RGVSYFVLPF LGQGIVDGDG NPIFLLIMIF [130]

8 .........T ..T....... .......D.. .E.......F .I..AFV.VM ISYIVGNV.. ...M...Y.. .......... ...L.F.... ......N... .......... ....Q.YDGY SFTG.C.I.. [130]

6 .Y.F..I..T .IT....... .......... .E........ .I..AFV.VM IGYMIGNV.. ...M...Y.. .......... ...L.F.... ......N... .......... ...EQ.YDGY SFTG.C.I.. [130]

5 ..V......T ..TH..K... .......S.. .E.......F .I..AVV.GM LGYMVGNV.. ...M...Y.. ........E. ...L.L.... ......N... .......... ....Q.YD.Y SF.W.C.I.. [130]

4 .........T ..T....... .......D.. .E........ .I..AFV.VM ISYIVGNV.. ...M...Y.. .......... ...L.F.... ......N... .......... ....Q.YDGY SFTG.C.I.. [130]

3 ...V....HT .IT....... .......N.. .E........ .I..VFV.VM ISYIVGNV.. ...M...Y.. .......... ...L.F.... ......N... .......... ....Q.YDGY SFTG.C.I.. [130]

3 ..V......T ..TH..K... .......S.. .E.......F .I..AVV.VM ISYIVGNV.. ...M...Y.. .......... ...L.F.... ......N... .......... ....Q.YDGY SFTG.C.I.. [130]

3 ...V....HT .IT....... .......N.. .E........ .I..VFV.VM ISYIVGNV.. ...M...Y.. .......... ...L.F..A. ......N... .......... ....Q.YDGY SFTG.C.I.. [130]

3 .........T ..T....... .......D.. .E.......F .I..AFV.VM ISYIVGNV.. ...M...Y.. .......... ...L.F.... ......N... .......... ....Q.YDGY SFTG.C.I.. [130]

2 ..V......T ..TH..K... .......S.. .E.......F .I..AVV.GM LGYMVGNV.. ...M...Y.. ........E. ...L.L.... ......N... .......... ....Q.YD.Y SF.W.C.I.. [130]

2 ...V....HT .IT....... .......N.. .E........ .I..VFV.VM ISYIVGNV.. ...M...Y.. .......... ...L.F.... ......N... .......... ....Q.YDGY SFTG.C.I.. [130]

2 .Y.F..I..T .IT....... .......... .E........ .I..AFV.VM IGYMVGNV.. ...M...Y.. .......... ...L.F.... ......N... .......... ...EQ.YDGY SFTG.C.I.. [130]

2 ..V......T ..TH..K... .......S.. .E.......F .I..AVV.GM LGYMVGNV.. ...M...Y.. ........E. ...L.L.... ......N... .......... ....Q.YD.Y SF.W.C.I.. [130]

1 .......... .......... .......... .......... .......... .......... .......... .......... .......... .......... .......... .......... .......... [130]

1 ..V......T ..TH..K... .......S.. .E.......F .I..AVV.VM ISYIVGNV.. .......Y.. .......... ...L.F.... ......N... .......... ....Q.YDGY SFTG.C.I.. [130]

1 .........T ..T....... .......D.. .E........ .I..AFV.VM ISYIVGNV.. ...M...Y.. .......... ...L.F.... ......N... .......... ....Q.YDGY SFTG.C.I.. [130]

1 ..V......T ..TH..K... .......S.. .E.......F .I..AVV.VM ISYIVGNV.. ...M...Y.. .......... ...L.F.... ......N... .......... ....Q.YDGY SFTG.C.I.. [130]

1 ..V......T ..TH..K... .......S.. .E.......F .I..AVV.GM LGYMVGNV.. ...M...Y.. ........E. ...L.L.... ......N... .......... ....Q.YD.Y SFTG.C.I.. [130]

1 ...V....HT .IT....... .......N.. .E........ .I..VFV.VM ISYIVGNV.. ...M...Y.. .......... ...L.F.... ......N... .......... ....Q.YDGY SFTG.C.I.. [130]

1 .......... .......... .......... .......... .......... .......... .......... .......... .......... .......... .......... ...E...... .......... [130]

1 .......... .......... .......... .......... .......... .......... .......... .......... .......... .......... .......... .......... .......... [130]

1 .........T ..T....... .......D.A .E.......F .I..AFV.VM ISYIVGNV.. ...M...Y.. .......... ...L.F.... ......N... .......... ....Q.YDGY SFTG.C.I.. [130]

1 .........T ..T....... .......D.. .E........ .......... .......... .......... .......... .......... .......... .......... .......... .......... [130]

1 ...V....HT .IT....... .......N.. .E........ .I..VFV.VM ISYIVGNV.. ...M...Y.. .......... ...L.F..A. ......N... .......... ....Q.YDGY SFTG.C.I.. [130]

1 ...T...... .......... .......... .......... .......... .......... .......... .......... .......... .......... .......... .......... .......... [130]

1 ...T...... .......... .......... .......... .......... .......... .......... .......... .......... .......... .......... .......... .......... [130]

1 .......... .......... .......... .......... .......... .......... .......... .......... .......... .......... .......... .......... .......... [130]

1 .......... .......... .......... .......... .......... .......... .......... .......... .......... .......... .......... .......... .......... [130]

1 T........T ..T....... .......D.. .E........ .I..AFV.VM ISYIVGNV.. ...M...Y.. .......... ...L.F.... ......N... .......... ....Q.YDGY SFTG.C.I.. [130]

1 ..V......T ..TH..K... .......S.. .E.......F .I..AVV.GM LGYMVGNT.. ...M...Y.. ........E. ...L.L.... ......N... .......... ....Q.YD.Y SF.W.C.I.. [130]

1 .........T ..T....... .......D.. .E........ .......... .......... .......... .......... .......... .......... .......... .......... .......... [130]

1 ..V......T ..TH..K... .......S.. .E.......F .I..AVV.GM LGYMVGNT.. ...M...Y.. ........E. ...L.L.... ......N... .......... ....Q.YD.Y SF.W.C.I.. [130]

1 .......... .......... .......... .......... .......... .......... .......... .......... .......... .......... .......... .......... .......... [130]

1 .........T ..T....... .......D.. .E.......F .I..AFV.VM ISYIVGNV.. ...MD..Y.. .......... ...L.F.... ......N... .......... ....Q.YDGY SFTG.C.I.. [130]

1 .........T ..T....... .......D.. .E........ .I..AFV.VM ISYIVGNV.. ...M...Y.. .......... ...L.F.... ......N... .......... ....Q.YDGY SFTG.C.I.. [130]

1 ..V......T ..TH..K... .......S.. .E.......F .I..AVV.GM LGYMVGNV.. ...M...Y.. ..FKRTS*-- ---------- ---------- ---------- ---------- ---------- [130]

13 VCFIVLVFLK WLDYDFTRLR REFLDTGFQK SLTKINWAMG AYYLVMQSLS YLEYEQGIQS TTVRHLILVF YLLFFMGGIK KLDTYLKEKL QEELNQEQTL RYRDMERYSR HIEELYKEIR SFRHDYTNLL [260]

8 NF..S.A... .......S.. K.I..KA... ...Q...I.. G.....E... FF....S... K......... .......V.. .......... Y.R.E...A. .......... ........V. .......... [260]

6 NF..S.A... .......S.. ..I....... .......I.. .....I.... .F........ .......... .......V.. .......D.. HDR.D...V. .......... ........V. .......... [260]

5 NF..S.A... .......S.. KGI..KD... ...Q...I.. .....I.N.. .F........ .......... .......I.. .......D.. H.R.....D. ...E...... ........V. .......... [260]

4 NF..S.A... .......S.. K.I..KA... ...Q...I.. G.....E... FF....S... K......... .......V.. .......... Y.R.E...A. .......... ........V. .......... [260]

3 NF..S.A... .......S.. ..I....... .......I.. .....I.... .F........ .......... .......V.. .......D.. HDR.D...V. ....I..... ........V. .......... [260]

3 NF..S.A... .......S.. ..I....... .......I.. .....I.... .F........ .......... .......V.. .......D.. HDR.D...V. ....I..... ........V. .......... [260]

3 NF..S.A... .......S.. ..I....... .......I.. .....I.... .F........ .......... .......V.. .......D.. HDR.D...V. .......... ........V. .......... [260]

3 NF..S.A... .......S.. K.I..KA... ..AQ...I.. G.....E... FF....S... K......... .......V.. .......... Y.R.E...A. .......... ........V. .......... [260]

2 NF..S.A... .......S.. KGI..KD... ...Q...I.. .....I.N.. .F........ .......... .......I.. .......D.. H.R.....D. ...E...... ........V. .......... [260]

2 NF..S.A... .......S.. ..I....... ..I....I.. .....I.... .F........ .......... .......V.. .......D.. HDR.D...V. ....I..... ........V. .......... [260]

2 NF..S.A... .......S.. ..I....... .......I.. .....I.... .F........ .......... .......V.. .......D.. HDR.D...V. .......... ........V. .......... [260]

2 NF..S.A... .......S.. KGI..KD... ...Q...I.. .....I.N.. .F........ .......... .......... .......D.. H.R.....D. ...E...... ........V. .......... [260]

1 .......... .......S.. K.I..KA... ...Q...I.. G.....E... FF....S... K......... .......V.. .......... Y.R.E...A. .......... ........V. .......... [260]

1 NF..S.A... .......S.. ..I....... .......I.. .....I.... .F........ .......... .......V.. .......D.. HDR.D...V. ....I..... ........V. .......... [260]

1 NF..S.A... .......S.. K.I..KA... ...Q...I.. G.....E... FF....S... K......... .......V.. .......... Y.R.E...A. .......... ........V. .......... [260]

1 NF..S.A... .......S.. ..I....... .......I.. .....I.... .F........ .......... .......V.. .......D.. HDR.D...V. ....I..... ........V. .......... [260]

1 NF..S.A... .......S.. ..I....... ...Q...I.. .....I.N.. .F........ .......... .......I.. .......D.. H.R.....D. ...E...... ........V. .......... [260]

1 NF..S.A... .......S.. ..I....... .......I.. .....I.... .F........ .......... .......V.. .......D.. HDR.D...V. .......... ........V. .......... [260]

1 .......... .......... .......... .......... .......... .......... .......... .......... .......... .......... .......... .......... .......... [260]

1 .......... .......... .......... .......... .......... .......... .......... .......... .......... .......... .......... .......... .......... [260]

1 NF..S.A... .......S.. K.I..KA... ...Q...I.. G.....E... FF....S... K......... .......V.. .......... Y.R.E...A. .......... ........V. .......... [260]

1 .......... .......... .......... .......... .......... .......... .......... .......... .......... .......... .......... .......... .......... [260]

1 NF..S.A... .......S.. ..I....... .......I.. .....I.... .F........ .......... .......V.. .......D.. HDR.D...V. .......... ........V. .......... [260]

1 .......... .......... .......... .......... .......... .......... .......... .......... .......... .......... .......... .......... .......... [260]

1 .......... .......... .......... .......... .......... .......... .......... .......... .......... .......... ...A...... .......... .......... [260]

1 .......... .......... .......... .......... .......... .......... .......... .......... .......... .......... .......... .......... .......... [260]

1 .......... .......... .......... .......... .......... .......... .......... .......... .......... .......... .......... .......... .......... [260]

1 NF..S.A... .......S.. K.I..KA... ...Q...I.. G.....E... FF....S... K......... .......V.. .......... Y.R.E...A. .......... ........V. .......... [260]

1 NF..S.A... .......S.. KGI..KD... ...Q...I.. .....I.N.. .F........ .......... .......I.. .......D.. H.R.....D. ...E...... ........V. .......... [260]

1 .......... .......... .......... .......... .......... .......... .......... .......... .......... .......... .......... .......... .......... [260]

1 NF..S.A... .......S.. KGI..KD... ...Q...I.. .....I.N.. .F........ .......... .......I.. .......D.. H.R.....D. ...E...... ........V. .......... [260]

1 .......... .......... .......... .......... .......... .......... .......... .......... .......... .......... .......... ........V. .......... [260]

1 NF..S.A... .......S.. K.I..KA... ...Q...I.. G.....E... FF....S... K......... .......V.. .......... Y.R.E...A. .......... ........V. .......... [260]

1 NF..S.A... .......S.. K.I..KA... ...Q...I.. G.....E... F.NMNKVFNQ RLFAISS*-- ---------- ---------- ---------- ---------- ---------- ---------- [260]

1 ---------- ---------- ---------- ---------- ---------- ---------- ---------- ---------- ---------- ---------- ---------- ---------- ---------- [260]

13 TSLRLGIEEE DMEQIKEIYD SVLRDSSQKL QDNKYDLGRL VNIRDRALKS LLAGKFIKAR EKNIVFNVEV PEEIQVEGMS LLDFLTIVSI LCDNAIEASA EASQPHVSIA FLKNGAQETF IIENSIKEEG [390]

8 .......... .......V.G ...K...... .N........ .....K.... ......L... D......... .......... .......... .........V .......... .......... .......... [390]

6 .......... .......V.. L..K...K.. .......... ..V....... .......... ..D....... .........R .......... .......... ..G....... ...S...... .......... [390]

5 .......... .......... ...K...E.. .......... ..V....... .......... D......... ........V. ......V... .........V ..C....... .F........ .......... [390]

4 .......... .......V.G ...K...... .N........ .....K.... ......L... D......... .......... .......... .........V .......... .......... .......... [390]

3 .......... .......V.. L..K...K.. .......... ..V....... .......... D......... .........R .......... .......... ..G....... ...S...... .......... [390]

3 .......... .......V.. L..K...K.. .......... ..V....... .......... ..D....... .........R .......... .......... ..G....... ...S...... .......... [390]

3 .......... .......V.. L..K...K.. .......... ..V....... .......... ..D....... .........R .......... .......... ..G....... ...S...... .......... [390]

3 .......... .......V.G ...K...... .N........ .....K.F.. ......L... D......... .......... .......... .........V .......... .......... .......... [390]

2 .......... .......... ...K...E.. .......... ..V....... .......... D......... .......... ......V... .........V ..C....... .F........ .......... [390]

2 .......... .......V.. L..K...K.. .......... ..V....... .......... D......... .........R .......... .......... ..G....... ...S...... .......... [390]

2 .......... .......V.. L..K...K.. .......... ..V....... .......... ..D....... .........R .......... .......... ..G....... ...S...... .......... [390]

2 .......... .......... ...K...E.. .......... ..V....... .......... D......... ........V. ......V... .........V ..C....... .F........ .......... [390]

1 .......... .......V.G ...K...... .N........ .....K.F.. ......L... D......... .......... .......... .........V .......... .......... .......... [390]

1 .......... .......V.. L..K...K.. .......... ..V....... .......... ..D....... .........R .......... .......... ..G....... ...S...... .......... [390]

1 .......... .......V.G ...K...... .N........ .....K.... ......L... D......... .......... .......... .........V .G........ .......... .......... [390]

1 .......... .......V.. L..K...K.. .......... ..V....... .......... ..D....... .........R .......... .......... ..D....... ...S...... .......... [390]

1 .......... .......... ...K...E.. .......... .....K.... .......... .......... .......... ......V... .........V ..C....... .......... .......... [390]

1 .......... .......V.. L..K...K.. .......... ..V....... .......... ..D....... .........R .......... .......... ..G....... ...S...... .......... [390]

1 .......... .......... .......... .......... .......... .......... .......... .......... .......... .......... .......... .......... .......... [390]

1 .......... .......... .......... .......... .......... .......... .......... .......... .......... .......... .......... .......... .......... [390]

1 .......... .......V.G ...K...... .N........ .....K.... ......L... D......... .......... .......... .........V .......... .......... .......... [390]

1 .......... .......... .......... .......... ..V....... .......... D......... .......... ......V... .........V ..C....... .F........ .......... [390]

1 .......... .......V.. L..K...K.. .......... ..V....... .......... ..D....... .........R .......... .......... ..G....... ...S...... .......... [390]

1 .......... .......... .......... .......... .......... .......... .......... .......... .......... .......... .......... .......... .......... [390]

1 .......... .......... .......... .......... .......... ......L... D......... .......... .......... .........V .......... .......... .......... [390]

1 .......... E......... .......... .......... .......... .......... .......... .......... .......... .......... .......... .......... .......... [390]

1 .......... .......... ....A..... .......... .......... .......... .......... .......... .......... .......... .......... .......... .......... [390]

1 .......... .......V.G ...K...... .N........ .....K.... ......L... D......... .......... .......... .........V .......... .......... .......... [390]

1 .......... .......... ...K...E.. .......... .....K.... ......L... D......... .......... ......V... .........V ..C....... .......... .......... [390]

1 .......... .......... .......... .......... .......... .......... .......... .......... .......... .......... .......... .......... .......... [390]

1 .......... .......... ...K...E.. .......... .....K.... ......L... D......... .......... .......... .........V .G........ .......... .......... [390]

1 .......... .......V.. L..K...K.. .......... ..V....... .......... ..D....... .........R .......... .......... .......... .......... .......... [390]

1 .......... .......V.G ...K...... .N........ .....K.... ......L... D......... .......... .......... .........V .......... .......... .......... [390]

1 ---------- ---------- ---------- ---------- ---------- ---------- ---------- ---------- ---------- ---------- ---------- ---------- ---------- [390]

1 ---------- ---------- ---------- ---------- ---------- ---------- ---------- ---------- ---------- ---------- ---------- ---------- ---------- [390]

13 IDISEIFSFG ASSKGEERGV GLYTVMKIVE SHPNTNLNTT CQNQVFRQVL TVIHAE* [447]

8 .......... .......... .......... .....S.... .......... ....T.. [447]

6 .......... V......... .......... .Y..AS.... ..D....... .M.PT.. [447]

5 .......... .......... .......... .....S.... ..DH...... ....I.. [447]

4 .......... .......... .......... .....S.... .......... ....T.. [447]

3 .......... V......... .......... .Y..AS.... ..D....... .M.PT.. [447]

3 .......... V......... .......... .Y..AS.... ..D....... .M.PT.. [447]

3 .......... V......... .......... .Y..AS.... ..D....... .M.PT.. [447]

3 .......... .......... .......... .....S.... .......... ....T.. [447]

2 .......... .......... .......... .....S.... ..DH...... ....I.. [447]

2 .......... V......... .......... .Y..AS.... ..D....... .M.PT.. [447]

2 .......... V......... .......... .Y..AS.... ..D....... .M.PT.. [447]

2 .......... .......... .......... .....S.... ..DH...... ....I.. [447]

1 .......... .......... .......... .....S.... .......... ....T.. [447]

1 .......... V......... .......... .Y..AS.... ..D....... .M.PT.. [447]

1 .......... .......... .......... .....S.... .......... ....T.. [447]

1 .......... .......... .......... .....S.... .......... ....T.. [447]

1 .......... .......... .......... .....S.... .......... ....... [447]

1 .......... V......... .........G .Y..AS.... ..D....... .M.PT.. [447]

1 .......... .......... .......... .......... .......... ....... [447]

1 .......F.. .......... .......... .......... .......... ....... [447]

1 .......... .......... .......... .....S.... .......... ....T.. [447]

1 .......... .......... .......... .....S.... ..DH...... ....I.. [447]

1 .......... V......... .......... .Y..AS.... ..D....... ....... [447]

1 .......... .......... .......... .......... .......... ....... [447]

1 .......... .......... .......... .....S.... .......... ....T.. [447]

1 .......... .......... .......... .......... .......... ....... [447]

1 .......... .......... .......... .......... .......... ....... [447]

1 .......... .......... .......... .....S.... .......... ....T.. [447]

1 .......... .......... .......... .....S.... ..DH...... ....I.. [447]

1 .......... .......... .......... .......... .......... ....... [447]

1 .......... .......... .......... .....S.... .......... ....T.. [447]

1 .......... .......... .......... .....S.... .......... ....... [447]

1 .......... .......... .......... .....S.... .......... ....T.. [447]

1 ---------- ---------- ---------- ---------- ---------- ------- [447]

1 ---------- ---------- ---------- ---------- ---------- ------- [447]

**BlpC**

The leader sequence is indicated in blue text, with the double-glycine cleavage motif underlined.

**Freq. Peptide sequence**

22 MDKKQNLTSF QELTTTELNQ ITGGGWWEEL LHETILSKFK ITKALELPIQ L* [52]

16 .......... .......... .....L..DI .YSLNII.HN N..G.HH... .. [52]

7 .......... .......... .I......DF .YRFNIIEQ. N..GFYQ... .. [52]

5 .......... .......... .I......DF .YRFNIIEQ. N..GFHQ... .. [52]

4 .......A.. .......... .....L..DI .YSLNII.HN N..G.HH... .. [52]

1 .......... .......... ....E..... .......... .......... .. [52]

1 .......A.. .......... .....L..DI .......... .......... .. [52]

1 .......... .......... .....L.... .......... .......... .. [52]

1 .......... .......... ........DF .YRFNIIEQ. N..GFHQ... .. [52]

1 .......... .......... .....L..D. .YNINRYAHY ..QE.HH... .. [52]

18 .......... .......... .....L..D. .YNINRYAHY ..*------- -- [52]

1 .......... .......... ........D. .YNINRYAHY ..*------- -- [52]

1 .......A.. *--------- ---------- ---------- ---------- -- [52]

**B) Bacteriocin precursor peptides**

Leader sequences are indicated in blue text, with the double-glycine cleavage motif underlined.

**BlpI**

**Freq. Peptide sequence**

29 MNTKMMEQFS VMDNEELEIV SGGRGNLGSA IGGCIGAVLL AAATGPITGG AATLICVGSG IMSSL* [66]

4 ......S... .......... .......... .......... .......... .......... ...... [66]

4 .........E I......... .......... .......... .......... .......... ...... [66]

1 .......... .......... .......... .......... .......... ....T..... ...... [66]

1 .......... .......... .......... .......... .......... .......... ..P... [66]

**BlpJ**

**Freq. Peptide sequence**

33 MNTKMLSQLE VMDTEMLAKV EGGYSSTDCQ NALITGVTTG IITGGTGAGL ATLGVAGLAG AFVGAHIGAI GGGLTCLGGM VGDKLGLSW* [90]

2 .......... .......... .....F.... .......... .......... .......... .......... .......... .......... [90]

1 .....M.... .......... .......... .......... .......... .......... .......... .......... .......... [90]

1 .......... .......... .......N.. .......... .......... .....T.... .......... .......... .......... [90]

1 .......... .......... .......... .......... .......... .......... .......... .EI.PKQVLE EQQQEVFN*- [90]

**BlpK**

**Freq. Peptide sequence**

17 MDTKMMSQFS VMDTEMLACV EGGGCNWGDF AKAGVGGAAA RGLQLGIKTG TWQGAATGAA GGAILGGVAY AATCWW* [77]

7 .......... .......... .......... .......G.. .......... .......... .......... ....... [77]

2 .N........ ...N....R. .......... .......G.. .......... .......... .......... ....... [77]

2 .N........ ...N...... .......... .......G.. .........R .......... .......... ....... [77]

1 .N........ .......... .......... .......... .......... .......... .......... ....... [77]

1 .......... ...N....R. .......... .......G.. .......... .......... .......... ....... [77]

**BlpKN**

**Freq. Peptide sequence**

10 MDTKMMSQFS VMDTEMLACV EGGGCNWGDF AKAGVGGAAV VAALGCAAGG VKYGKILGPW GAAIGGIGGA VVCGYLAYTA TS* [83]

**BlpM**

**Freq. Peptide sequence**

30 MDTKIMEQFH EMDITMLSSI EGGKNNWQTN VLEGGGAAFG GWGLGTAICA ASGVGAPFMG ACGYIGAKFG VDLWAGVTGA TGGF* [85]

10 .N..M..... ....A..... .......... .F...S.... .......... .......... .......... .A........ ..... [85]

3 .......... .......... .......... .......... .......... .......... .......... .A........ ..... [85]

2 .N..M..... .......... .......... .......... .......... .......... .......... .......... ..... [85]

2 .N..M..... .......... .......... .......... .......... .......... .......... .A........ ..... [85]

1 .......... .......... .......... .......... ......V... .......... .......... .......... ..... [85]

1 .......... .......... .......... .......... .......... .......... .......... .A........ .S... [85]

1 .......... .......... .......... .......... ......RDSY LCCEWCWSTI YGSMWIHRS* ---------- ----- [85]

**BlpN**

**Freq. Peptide sequence**

33 MNTYCNINET MLSEVYGGNS GGAAVVAALG CAAGGVKYGR LLGPWGAAIG GIGGAVVCGY LAYTATS* [68]

14 .......... .......... .......... .........K I......... .......... ........ [68]

2 .......... .......... .......... ........R. .......... .......... ........ [68]

1 .......... .......... .......... .........K I......... .......... .S...... [68]

1 .......... .......... .......... .......... ...L...... .......... ........ [68]

**BlpMN1**

**Freq. Peptide sequence**

1 MDTKIMEQFH EMDITMLSSI EGGKNNWQTN VLEGGGAAFG GWGLGTAICA ASGVGAPFMG ACGYIGAKFG VDLWAGVTGA TGGVKYGRLL GPWGAAIGGI GGAVVCGYLA YTATS* [116]

**BlpMN2**

**Freq. Peptide sequence**

1 MDTKIMEQFH EMDITMLSSI EGGKNNWQTN VLEGGGAAFG CAAGGVKYGR LLGPWGAAIG GIGGAVVCGY LAYTATS* [78]

**BlpO**

**Freq. Peptide sequence**

24 MNTKMMSQFS VMDNEMLACV EGGDIDWGRK ISCAAGVAYG AIDGCATTV* [50]

20 .D.......A .......... .......... .......... .......... [50]

4 .D.......A .......... .......... ...T...... .......... [50]

3 .D........ .......... .......... .......... .......... [50]

2 .......... .I........ .......... .......... .......... [50]

1 .D........ .I........ .......... .......... .......... [50]

1 .D........ ...T...... .......... .......... .......... [50]

1 .......... .......... ...Y...... .......... .......... [50]

1 .......... .......... .........E .......... .......... [50]

**BlpQ**

**Freq. Peptide sequence**

1 MNTKTMSQFA IMDTEMLDRI EGGIFGVDDV VFWTGVGSYV AGRVVDAAID DFTNQCRKNP HQWFCVRV* [69]

6 ....M....S V..N...... .........A L..A.L.-.. ..SI..T... .......... ......... [69]

4 ....M.E..S V..N...... .........A L..A.L.-.. ..SI..T... .......... ......... [69]

1 ....M....S V..N...... .........A L..A.L.-.. ..SI..T... ......Q... ......... [69]

**BlpQM**

**Freq. Peptide sequence**

1 MNTKMMSQFS VMDNEMLDRI EGGIFGVDDA LFWAGLGTAI CAASGVGAPF MGACGYIGAK FGVALWAGVT GATGGF* [77]

**PncT**

**Freq. Peptide sequence**

11 MEKIDYITLN EVELETISGG DDCFIGDIGC IGWGLLKSIG GMIKPAPYVP PVCIPKSSWN PAPPVPC* [68]

1 .N.N...A.. .......... .....A.V.. .......... .......... .......... ........ [68]

**BlpD**

**Freq. Peptide sequence**

4 MNTKMMSQFS VMDNEMLASI EGGTDWGTVG KGAVYGAGIG VAMCAVGGLL TGGSTWAMTA GCAWAGAKLG GSFTAIADNL WP* [82]

**BlpE**

**Freq. Peptide sequence**

4 MFNYKIVDNQ ELSNISGGGL GGDVVVGALS GAFQAGQSCI AGGPQAYLIC ATGGAIVGGI LAYGLRPPK* [70]

**BlpW**

**Freq. Peptide sequence**

1 MNTKMMEQFE IMDTEMLACV EGGGWIKCGL GVVGGALTGG VAGGAVGTVT LPFFGTVSGA AAGFWGGGAT GAATFC* [77]

**PncW**

**Freq. Peptide sequence**

7 MDTKMMSQFA VMDNEMLARI EGGDVSDIYR GYANQVSPFG SYPPILKNSG PFPVSGYCPR GYHDRGYIGA GFHLCGI* [78]

5 .........S .......V.T .D........ .....R...A ...S...... ........L. .......... ........ [78]

4 .N.......S .......V.T .D........ .....R...A ...S...... ........L. .......... ........ [78]

**C) Membrane proteins with a putative function in immunity**

**PncG**

**Freq. Peptide sequence**

19 MKKKILIIFV LYLIMSIFLY PLRESAWYQL FYTIAYVIAV MIYFAINKKK GEKK* [55]

11 .........I .......... .....I.... .......... .....LT... .A... [55]

2 .......... .......... .......... .......... .......... A.... [55]

1 .........I .......... .....I.... .......... ....VLT... .A... [55]

1 .......... .......... .......... .......... .....LT... .A... [55]

1 .........A .......... .....I..N. .......... .....LT... .A... [55]

1 .........A .......... .....I.... .......... .....LT... .A... [55]

1 .........A .......... .....I.... .......... ...L.LT... .A... [55]

20 .......... .......... .....I..N. ......M... ....SLI... -.... [55]

7 .......... .......... .......... ....V.M... ....SLI... -.... [55]

1 .....F..LI .......... .....I..N. ......M... I....LI... -.... [55]

1 ....Y.SFSS CI*------- ---------- ---------- ---------- ----- [55]

1 ....Y*---- ---------- ---------- ---------- ---------- ----- [55]

1 ....Y*---- ---------- ---------- ---------- ---------- ----- [55]

**BlpL**

Residues derived from IS element insertions are indicated in pink text. The pink question mark (?) represents an unknown end to this BlpL allele: no in-frame stop codon was detected within the first 25bp of the IS element (see Methods section in main text).

**Freq. Peptide sequence**

7 MKTFLAKKRN IFLARLFLGQ LPLLVSTYLF LSRQFLNFSV VFQFLLVVIN LASILVTVYL TREMRIREFE DDDLVSPRTN QLMFIGLTGF MSIICLYRGI TAGESYQQLI AYIGAVLCLI IMLLLMWGLK [130]

7 .......... .......... .......... .......... .......... .......... .......... .......... .......... .......... ........I. .......... .......... [130]

4 ..KIF.T... V..V..L... I..V...... .........L .......I.. .....A.... .......K.. .......... .......... .......... ..A.....I. .........L .....I.... [130]

3 ....F..... .......... .......... .......... .......... .......... .......... .......... .......... .......... ........I. .......... .......... [130]

1 ....F..... .......... .......... .......... .......... .......... .......... .......... .......... .......... ........I. .......... .......... [130]

1 .......... .......... .......... .......... .......... .......... .......... .......... .......AD. .FV....... SET.F..... ....VFSACL SRFCSCGA*- [130]

1 ---------- -LIKKNHILC EIVPG.VAFA CFYLSISI.S .......... .T........ .....L.... .......... .......... .......... .......... .....I.... .....I.... [130]

7 ---------- -LIKKNHILC EIVPG.VAFA CFYLSISI.S .......... .T........ .....L.... .......... .......... .......... .......... .....I.... .....I.... [130]

4 ---------- -LIKKNHILC EIVPG.VAFA CFYLSISI.S .......... .......... ......K... .......... .......... .......... ........I. .....I...T .....I.... [130]

3 ---------- -LIKKNHILC EIVPG.VAFA CFYLSISI.S .......... .T........ .....L.... .......... .......... .......... ........I. .....I...T .....I.... [130]

3 ---------- -LIKKNHILC EIVPG.VAFA CFYLSISI.S .......... .......... ......K... .......... .I........ .......... ........I. .....I.... .I.F.I.... [130]

2 ---------- -LIKKNHILC EIVPG.VAFA CFYLSISI.S .......... .T........ .....L.... .......... .....D.... .......... .......... .....I.... .....I.... [130]

2 ---------- -LIKKNHILC EIVPGAVAFA CFYLSISI.S .......... .......... ......K... .......... .......... .......... .......... .....I.... .....I.... [130]

1 ---------- -LIKKNHILC EIVPG.VAFA CFYLSISI.S .......... .T........ .....L.... .......... .......... .......... ........I. .....I...F .T........ [130]

1 ---------- -LIKKNHILC EIVPG.VAFA CFYLSISI.S .......... .T........ .....L.... .......... .......... .......... ........I. .....I...F .T........ [130]

4 ---------- -LIKKNHILC EIVPG.VAFA CFYLSISI.S .......... .T........ .....L.... .......... .......... .FV....... SET.F..... V...VFSACL SRFCSCGA*- [130]

1 ---------- -LIKKNHILC EIVPG.VAFA CFYLSISI.S .......... .T........ .....L.... .......... .......AD. .FV....... SET.F..... V...VFSACL SRFCSCGA*- [130]

1 ---------- -LIKKNHILC EIVPG.VA.A CFYLSISI.S .......... .T........ .....L.... .......... .......AD. .FV....... SET.F..... V...VFSACL SRFCSCGA*- [130]

1 ---------- -LIKKNHILC EIVPG.VAFA CFYLSISIFY FYFVSFPISF SSY*------ ---------- ---------- ---------- ---------- ---------- ---------- ---------- [130]

2 .......NGT SS.RDCS*-- ---------- ---------- ---------- ---------- ---------- ---------- ---------- ---------- ---------- ---------- ---------- [130]

7 YYKK*----- -- [142]

7 .....----- -- [142]

4 .....----- -- [142]

3 .....----- -- [142]

1 .*-------- -- [142]

1 ---------- -- [142]

1 ..NPNHSYLS Q? [142]

7 .....----- -- [142]

4 .....----- -- [142]

3 .....----- -- [142]

3 .....----- -- [142]

2 .....----- -- [142]

2 .....----- -- [142]

1 H....----- -- [142]

1 .....----- -- [142]

4 ---------- -- [142]

1 ---------- -- [142]

1 ---------- -- [142]

1 ---------- -- [142]

2 ---------- -- [142]

**BlpP**

**Freq. Peptide sequence**

58 MKAIFFIILF AFQTYLIYLS ISISDKKQKT IELTSLNCFV ILFLIYDKLI FLFIAYVFLI IFILNLFRN* [70]

2 .......... .......... .......... .......... .......... .......... .......C.. [70]

1 .......... .......... .......... .......... ......N... .......... .......... [70]

1 .......... .......... .......... .......... .......... ..SYCLC.FD N.YIK.VP*- [70]

1 .......... .......... ......NKRL LN*------- ---------- ---------- ---------- [70]

**PncM**

**Freq. Peptide sequence**

38 MDKKKIVSTI ICIVFLVVSV DNFFRDLTPL LFILNIIGLS CFSVLTYINI KEILLNISK* [60]

7 .......... .......... .......... .........T ..L....T.. .......... [60]

3 .......... .......... .......... .......... ........K. .......... [60]

2 .......... .......... .......... .......... .......T.. .......... [60]

1 K......... .......... .......... .........T ..L....T.. .......... [60]

1 .......... VLL...I... A......N.. .......... .......... ..M....R.. [60]

1 .......... .......... .......... .........T ..L....... .......... [60]

1 .........N MYSISCS..R *--------- ---------- ---------- ---------- [60]

**BlpF**

**Freq. Peptide sequence**

3 MLNETMIQLV LIAICCVYII FNTKADKNQK RGYRTALYLF VMAGIISYIM NYLNWLDFFL LITPIMCLFK FEDKWS* [77]

1 .......... .......... .......... .......... .......... ........SY *--------- ------- [77]

**BlpX**

**Freq. Peptide sequence**

3 MKYRLFFVIF LSSVLDILLG TFLQISIVSI GWLVLYSGLF EVGVFLLANK GVAVKIKEVD IRNRFKFIFG KTLWFQILLL IFLIIKLYLG LDARLILFYG HIFIVFNALM YLLSSSQVSL KKTNCLLNLT [130]

3 .......... .......... .......... .......... .A........ .......... .......... .......... .......... .......... .......... .......... .......... [130]

1 .......... .......... .......... ....F..... .A........ .......... .......... .......... .......... .......... .......... .......... .......... [130]

2 .......... .......... .......... .......... .......... .......... .......... .......... .......... .......... .......... .......... ..KQTVFLIL [130]

1 .......... .......... .......... .......... .A........ .......... .......... .......... .......... .......... .......... .......... ..KQTVFLIL [130]

37 .......... .......... .......... .......... .A........ .......... .......... .......... .......... .......... .......... .......... ..NKLSS*-- [130]

5 .......... .......... .......... .......... .A........ .......... .......... .......... .......... .......... .......V.. .......... ..NKLSS*-- [130]

4 .......... .......... .......... ....F..... .A........ .......... .......... .......... .......... .......... .......... .......... ..NKLSS*-- [130]

3 .......... .......... .......... .......... .......... .......... .......... .......... .......... .......... .......... .......... ..NKLSS*-- [130]

1 .......... .......... .......... .......... .A........ .......... .......... .......... .......... .......... .......... .......... ..NKLSS*-- [130]

1 .......... .......... .......... .......... .A........ .......... ...L...... .......... .......... .......... .......... .......... ..NKLSS*-- [130]

1 ......L... ...M.N.... ......N... ..I....... .A........ .......... .......... .......F.. ...MV..... .......... .......... .......... ..NKLSS*-- [130]

3 AVESDKVRS* [140]

3 ...P...... [140]

1 ...P...... [140]

2 QQ*------- [140]

1 QQ*------- [140]

37 ---------- [140]

5 ---------- [140]

4 ---------- [140]

3 ---------- [140]

1 ---------- [140]

1 ---------- [140]

1 ---------- [140]

**BlpZ**

**Freq. Peptide sequence**

12 MYKHLFFLDS KTLDRLTPYI LVLASDTIAF NVFVLTFVSA VVFNFLNSML ALMAIFIGAG YVVGFWLLKW FVLERLDLKN DV* [83]

3 .......... .......... .......... .......... .......... .......... .......... ......E... ... [83]

2 .......... .......... .......... .......... .......... .......... .......... ......E..D .A. [83]

1 .......... .......... ...V...... .......... .......... .......... .......... .......... ... [83]

1 .......... .......... .......... .......... .......... ......L... .......... ......E..D .A. [83]

1 .......... ....W..... .......... .......... .I.....P.. S.....L... .......... ......E..D ... [83]

1 ...R...... ....W..... .......... .......... .......P.. S.....L.G. .......... ......E... NA. [83]

43 .......... .......... .......... .......... .......... .......... ........IL NENQ.AN*-- --- [83]

2 .......... .......... ...S...... .......... .......... .......... ........IL NENQ.AN*-- --- [83]

2 .......... ....W..... .......... .......... .I........ .......... ........IL NENQ.AN*-- --- [83]

1 .......... .......... .......... .......... ....S..... .......... ........IL NENQ.AN*-- --- [83]

1 .......... ....W..... .......... .......... .......... .......... ........IL NENQ.AN*-- --- [83]

1 .......... .......S.. .......... .......... .......... .......... ........IL NENQ.AN*-- --- [83]

1 .......... .......... .......... .......... .......... .......RGW LCGRILVTNT Q*-------- --- [83]

2 .......... .......... .......... .......... .......... ......L*-- ---------- ---------- --- [83]

4 .......*-- ---------- ---------- ---------- ---------- ---------- ---------- ---------- --- [83]

**D) CAAX amino terminal proteases**

**BlpG**

The predicted coding sequence for BlpG starts with an alternative start codon (GTG), which in other positions would encode V.

**Freq. Peptide sequence**

4 MFVGFLVYVI VATVGYTINQ GDYFQNEHLF IIAKTFLISL SVAYAKWFDM ISLRRLTKKE VLLFIASFLL CVLVNIGYHS LFTVSSGAGY QHLEAASTGI SLSFIASATV FGPILEEFVF RGILQGVVFE [130]

4 NSWLGLVLTA SLFSFLHAPY DFPSFIYYLF GGFMLGFAYK KSQKLSVAIL VYICYNCLSF L* [192]

**BlpY**

**Freq. Peptide sequence**

16 MKKYQLLFKI SAVFSYLFFV FGLSQLTLIV QNYWQFSSQI GNFVWIQNIL SLLFIGVMIV VLVKTGHGYL FRIPRKKWLW YSILTVLVVV FQISFNVQTA KHVQSTAEGW AVLIGYSGTN FAELGIYIAL [130]

12 .......... .......... .....M...I .......... ........F. ....S....W I......... .H........ .......... L......... .......... .......... ........T. [130]

8 .......... .......... .......... .......... .......... ....S....W I......... .......... .......... LH........ .......... N......... .......VT. [130]

5 .........T ..IL...... .......... .......... ..LF...... .......... .......... .......... .......... .......... .......... .......... .......... [130]

5 .......... .......... .S........ .......... ..LF...... .......... .......... .......... ........L. .......... .......... .......... .......... [130]

4 .......... .......... .......... .......... .......... ....S....W I......... .......... ........L. L.......I. .......... .......... .......VT. [130]

4 .......... .......... .....M...I .......... ........F. ....S....W I......... .H........ .......... L......... .......... .......... .......VT. [130]

3 .......... .......... .......... .......... ..LF...... .......... .......... .......... .......... .......... .......... .......... .......... [130]

2 .......... .......... .......... .......... .......... .......... .......... .......... .......... .......... .......... .......... .......... [130]

2 .......... .......... .......... .......... .......... ....S....W I.A....... .......... ........L. LH......I. .......... .......... .......VT. [130]

2 .......... .......... .......... .......... .......... ....S....W I.A....... .......... ........L. LH......I. .......... .......... .......VT. [130]

1 .......... .......... .......... .......... .......... ....S....W I.A....... .......... ........L. LH......I. .......... .......... .......VT. [130]

1 .......... .......... .......... .......... ..LF...... .......... .......... .......... .......... L......... .......... .......... .......... [130]

1 .......... .......... .......... .......... ..LF...... .......... .......... .......... .......... .......... .......... .......... .......... [130]

1 .......... .......... .......... .......... .......... ....S....W I......... .......... .......... IH.F...... .......... T......... .......VT. [130]

1 .......... .......... .......... .......... ..LF...... .......... .......... .......... .......... L......... .......... .......... .......... [130]

1 .......... .......... .......... .......... .......... N......... .......... .......... .......... .......... .......... .......... .......... [130]

1 .......... .......... .....M...I .......... ........F. ....S....W I......... .H........ .......... L......... .......... .......... .......VT. [130]

1 .......... .......... .S........ .......... ..LF...... .......... .......... .......... ........L. .......... .......... .......... .......... [130]

1 .......... .......... .......... .......... .......... ....S..T.W I......... .H........ ........L. L.......I. .......... .......... .......VT. [130]

1 .......... .......... .......... .......... ..LF...... .......... .......... .......... .......... .......... .......... .......... .......... [130]

1 .......... .......... .......... .......... .......... ....S....W I......... .......... ........L. L......... .......... .......... ........T. [130]

1 .......... .......... .....M...I .......... ........F. ....S....W I......... .H........ .......... L......... .......... .......... ........T. [130]

1 ..RHAI...T ..IL....LF ....WT.QFW S...E...WV ..II..R..I .....CL.VW I..RS..A.. .........S ..V....AA. VL.C..YL.. ....G.N... NLF.A..E.. ...F.V.LT. [130]

1 .......... .......... .......... .......... .......... ....S....W I......... .......... .......... LH........ .......... N......... .......VT. [130]

1 .......... .......... .......... .......... .......... .......... .......... ......NGFG IRF*------ ---------- ---------- ---------- ---------- [130]

16 FFLVPLMEEL IYRGLLQHAF FKHSRFGLDL LLPSILFALP HFSSLPSLLD IFVFATVGII FAGLTRYTKS IYPSYAVHVI NNIVATFPFL LTFLHRVLG* [230]

12 ...T...... .......... .......... .......... .......... ..I...S... ..S....... .......... ...F..L... .......... [230]

8 ...T...... .......... .......... .......... ..L....... ......F... .......... .......... .......... .......... [230]

5 .......... .......... .......... .......... .......... .......... .......... .......... .......... .......... [230]

5 .......... .......... .......... .......... .......... .......... .......... .......... .......... .......... [230]

4 ...T...... .......... .......... F......... .......... ......F... .......... .......... .......... .......... [230]

4 ...T...... .......... .......... .......... ..L....... ......F... .......... .......... .......... .......... [230]

3 .......... .......... .......... .......... .......... .......... .......... .......... .......... .......... [230]

2 .......... ...E...... .......... .......... .......... .......... .......... .......... .......... .......... [230]

2 ...T...... .......... .......... F......... ...R...... ......F... .......... .......... .......... .......... [230]

2 ...T...... .......... .......... F......... .......... ......F... .......... .......... .......... .......... [230]

1 ...T...... .......... .......... .......... ..L....... ......F... .......... .......... .......... .......... [230]

1 .......... .......... .......... .......... .......... .......... .......... .......... .......... .......... [230]

1 .......... .......... .......... .......... .......... .......... .......... .......... .....I.... .......... [230]

1 ...T...... .......... .......... .......... ..L....... ......F... .......... .......... .......... .......... [230]

1 .......... .......... .......... .......... ..L....... ......F... .......... .......... .......... .......... [230]

1 .......... .......... .......... .......... .......... .......... .......... .......... .......... .......... [230]

1 ...T...... .......... .......... .......... .......... ......F... .......... .......... .......... .......... [230]

1 .......... .......... .......... .......... .......... ....T..... .......... .......... .......... .......... [230]

1 ...T...... .......... .......... .......... ...N...... ......F... .......... .......... .......... .......... [230]

1 .......... .......... .......... .......... ..L....... .......... .......... .......... .......... .......... [230]

1 ...T...... .......... .......... .......... .......... ..I...S... ..S....... .......... ...F..L... .......... [230]

1 ...T...... .......... .......... .......... .......... ..I...S... .......... .......... ...F..L... .......... [230]

1 IV.G...... VC........ ..D..W.Q.. .F..F..... ........A. ...YTA..CL ..C....... .....SI.IV ...I.NL... .......... [230]

1 ...T...... .......... .......... .......... ..L....... .......... .......... .......... .......... .......... [230]

1 ---------- ---------- ---------- ---------- ---------- ---------- ---------- ---------- ---------- ---------- [230]

**PncP**

Residues derived from IS element insertions are indicated in pink text.

**Freq. Peptide sequence**

16 MEFFDKFHAL CFGFLVLIIV ITVPYTINHG DFFQNESALI IVSLLVTSLS VAYARKFEMI SFGMLSKKQL LLFIAIFLLS VLETLVYIHF FAVSSGSGVQ HLAEVSRGIS LSLILTTSVF GPIQEELIFR [130]

13 .......... .......... .......... G......... L......... .......... .......... .......... .......... .......... .......... .......... .......... [130]

9 .......... .......... .......... G......... L......... .......... .......... .......... .......... .......... .......... .......... .......... [130]

7 .......... .......... .I........ .......... L......... .......... .......... .......... .......... .......... .......... .......... .......... [130]

4 .......... .......... .I........ .......... L......... .......... .......... .......... .......... .......... .......... .......... .......... [130]

4 .......... .......... .......... .......... .......... .......... .......... .......... .......... .......... .......... .......... .......... [130]

3 .......... .......... .......... G......... L......... .......... .......... .......... .......... .......... .......... .......... .......... [130]

3 .......... .......... .I........ .......... L......... .......... .......... .......... .......... .......... .......... .......... .......... [130]

2 .......... .......... .......... .......... L......... .......... .......... .......... .......... .......... .......... .......... .......... [130]

2 .......... .......L.. .......... .......E.. .......... .V...Q.... .......... ....E..... .......... ..I...A... .......... .......... .......... [130]

2 .......... .......... .......... .......... .......... .......... .......... ....V..... .......... .......... .......... .......... .......... [130]

1 .......... .......... .......... .......... L......... .......... .......... .......... .......... .......... .......... .......... .......... [130]

1 .......... .......... .......... .......... ...F...... .......... ........E. .......... .......... ..I...A... .......... .......... .......... [130]

1 .......... .......... .......... GV........ L......... .......... .......... .......... .......... .......... .......... .......... .......... [130]

1 .......... .......... .......... V......... L......... .......... .......... .......... .......... .......... .......... .......... .......... [130]

1 .......... .......... .......... .......... .......... .......... .......... .......... .......... .......... .......... .......... .......... [130]

1 .......... .......... .......... .......... T......... .......... .......... .......... .......... .......... .......... .......... .......... [130]

1 .IWV...... .......... .......... G......... L......... .......... .......... .......... .......... .......... .......... .......... .......... [130]

1 .......... .......... .......... ......F... L......... .......... ........DI ....T..... L......... ..I...A... .....R.... ...V..S... .......... [130]

1 .......... ......I... .......... .......... .......... .......... .......... .......... .......... ......A... .......... .......... .......... [130]

1 .......... .......... .......... .......... L......... .......... .......... .......... .......... .......... .......... .......... .......... [130]

1 .......... .......... .........E V......... L......... .......... .......... .......... .......... .......... .......... .......... .......... [130]

1 .......... .......... .I........ .......... L......... .......... .......... .......... .......... .......... .......... .......... .......... [130]

1 .......... .......L.. .......... .......E.. .......... .......... ........E. .......... .......... ......A... .......... ......S... .......... [130]

1 .......... .......DLF GI*------- ---------- ---------- ---------- ---------- ---------- ---------- ---------- ---------- ---------- ---------- [130]

16 GLLQGAVFDN SWLGLVLTSS LFSFMHGPSN VPSFIFYLLG GLLLGLAYKK SQNLWVSTLV HMFYNSWPLL YYL* [204]

13 .......... .......... .......... .......... .....F.... .......... ..L....... .... [204]

9 .......... .......... .......... .......... .....F.... .......... .......... .... [204]

7 .......... .......... .......... .......... .......... .......... ..L....... .... [204]

4 .......... .......... .......... .......... .......... .......... .......... .... [204]

4 .......... .......... .......... .......... .....F.... .......... ..L....... T... [204]

3 .......... .......... .......... .......... .......... .......... .......... .... [204]

3 .......... .......... .......... .......... .......... .......... Q.L....... .... [204]

2 .......... .......... .......... .......... .......... .......... .......... .... [204]

2 .......... .......... .......... .......... .......... .......... .......... .... [204]

2 .......... .......... .......... .......... .......... .......... .......... .... [204]

1 .......... .......... .......... .......... .....F.... .......... ..L....... .... [204]

1 .......... .......... .......... .......... .....F.... .......... .......... T... [204]

1 .......... .......... .......... .......... .....F.... .......... ..L....... .... [204]

1 .......... .......... .......... .......... .....F.... .......... ..L....... .... [204]

1 .......... .......... .......... .......... .....F.... .......... ..L....... .... [204]

1 .......... .......... .......... .......... .......... .......... .......... .... [204]

1 .......... .......... .......... .......... .....F.... .......... .......... .... [204]

1 .......... .......... .......... .......... .....F.... .......... .....A.... .... [204]

1 .......... .......... .......... .......... .....F.... .......... ..L....... .... [204]

1 .......... .......... .......... .......... S....F.... .......... ..L....... T... [204]

1 .......... .......... .......... .......... .....F.... .......... ..L....... .... [204]

1 .......... .......... .......... .......... .....F.... .......... ..L....... .... [204]

1 .......... .......... .......... .......... .....F.... .......... .......... .... [204]

1 ---------- ---------- ---------- ---------- ---------- ---------- ---------- ---- [204]

**E) Hypothetical proteins**

**BlpT**

**Freq. Peptide sequence**

48 MTDTDPIKRA HTLITDLNKA YQACKQASAD DVRFQEQLNS ILGFLAKAET VDNRFLIELE KFYQTSSLLM GLSALDPDAP TRAAWRAYDR FHFDQVKTKL ILNENQRAN* [110]

12 .......... .......... .......... .......... .......T.. .......... .......... .......... .......... .......... .......... [110]

6 .......... Q......... .......T.. .......... .......... .......... .......... .......... .......... .......... .......... [110]

3 .......... .......... .......... .......... .......... .......... ....I..... .......... .......... .......... .......... [110]

2 .......... .......... .......... .L........ .......... .......... .......... .......... .......... .......... .......... [110]

2 .......... Q......... .......T.. .......... .......... .......... .......... .......... .......... .......... S.YGPTIIL. [110]

1 .......... .......... .......... ........D. .......... .......... .......... .......... .......... .......... S.YGPTIIL. [110]

1 .......... .......... .......... .......... .......... .......... .......... .......... .......... .......... S.YGPTIIL. [110]

1 .......... .......... .......T.. ........D. .......... .......... .......... .......... .......... .......... S.YGPTIIL. [110]

1 .......... .......... .......... .......... .......... .......... .......... .......... .......... ....P..... .......... [110]

1 .......... .......... .......... ..C....... .......... .......... .......... .......... .......... .......... .......... [110]

1 .......... Q......... .......... .......... .......... .......... .......... .......... .......... .......... .......... [110]

**BlpV**

The predicted coding sequence for BlpV starts with an alternative start codon (TTG), which in other positions would encode L.

**Freq. Peptide sequence**

4 MGRGSFSKNY MVWLDNHVRI LMAALNTSLT TNIKFGLVGT VGGRFAGYGM YVLGYPGDYL DITNHRVVSA KTSHKSYQIL END* [84]
