# Supplementary material for: Genomic analyses of pneumococci reveal a wide diversity of bacteriocins – including pneumocyclicin, a novel circular bacteriocin
Source: BMC Genomics. 2015 Jul 28;16(1):554. doi: 10.1186/s12864-015-1729-4 (PMC4517551; doi:10.1186/s12864-015-1729-4)
Supplement: Additional file 3: — Alignment S2. BlpR, BlpH and BlpC allele combinations (variable residues only), ordered by pherogroup. [file 12864_2015_1729_MOESM3_ESM.docx]

**Alignment S2. BlpR, BlpH and BlpC allele combinations (variable residues only), ordered by pherogroup.**

This alignment contains the various combinations of BlpR, BlpH and BlpC alleles (represented by their variable residues) present among the 79 prototypes. The various combinations have been divided into pherogroups, depending on BlpC sequence, and labelled with a letter in square brackets.

The pherogroups are equivalent to the pherotypes previously described by Reichmann & Hakenbeck [[1](#_ENREF_1)] and Son *et al*. [[2](#_ENREF_2)], and minor variants of these, in the following manner:

A = SpiP class IIc [[1](#_ENREF_1)] or BlpC type 6A [[2](#_ENREF_2)];

B = SpiP class IIa [[1](#_ENREF_1)] or BlpC type R6 [[2](#_ENREF_2)];

C = SpiP class IIb [[1](#_ENREF_1)] or BlpC type P164 [[2](#_ENREF_2)];

D = SpiP class I [[1](#_ENREF_1)] or BlpC type TIGR4 [[2](#_ENREF_2)].

A fifth group, labelled Hybrid/mosaic, contains prototypes for which BlpC and BlpH are mismatched, or are composed of a mosaic of sequences from different pherogroups.

For each separate row in the alignment, the number indicates the frequency of this BlpR/H/C combination among 79 prototypes, and in brackets the specific Group prototype(s) in which this was found.

The given residue positions are relative to the start of BlpR.

Identical residues are represented by a dot (.), absent residues by a dash (-), and stop codons by an asterisk (*).

1. Reichmann P, Hakenbeck R: **Allelic variation in a peptide-inducible two-component system of *Streptococcus pneumoniae*.** *FEMS Microbiol Lett* 2000, **190:**231-236.

2. Son MR, Shchepetov M, Adrian PV, Madhi SA, de Gouveia L, von Gottberg A, Klugman KP, Weiser JN, Dawid S: **Conserved Mutations in the Pneumococcal Bacteriocin Transporter Gene, blpA, Result in a Complex Population Consisting of Producers and Cheaters.** *MBio* 2011, **2**.

**BlpR BlpH BlpC**

[ 1111111122 22222222222222222222222233333333333333333333333333333333333333444444444444444444444444444444444444555555555555555556666666666666666 777777777777777777777]

[ 233455555556666666771236689904 44455555566777889999999900000111122222333466666666677777889999000111222222333333333344445666677889122233334556668890001244667778899 011122222223333333334]

[ 601923456890123456895515794573 78903568903468681235678901234014901235025301345678902478134789239047234789012345678901234478915015746701489243795636898047681290801 158923567890123467890]

**Pherogroup**

**[A]**

n=6(1b,26,30,31,32,33) AIPEAHQLFLDIEIRNEEEQAASPPFRLAN MYIFIYTITNEGTELIAFVVMIGYMIGNVMEYSFLLLGLFGNEQYDGYSFTGCINFSASREITGTKIAIQSYFEYEQGIQSTTVRHLIVDHDRDVDMVDVDLKDKDVRLIEDMRIAAGLSSVEYASDQMPT TTGLDIYSLNIIKHNNKGLHH

n=2(9,15a) ..................DR.......... .........................V......................................................................................................... .....................

n=3(6,19k,23b) ET................DR.......... .N.VLH.....N....V.....S.IV..............A.G........................................................................................ .....................

n=1(2) ..................DR....A..... .N.VLH.....N....V.....S.IV................G...............................................................................G........ .....................

n=2(17ab) ..................DR....A..... .N.VLH.....N....V.....S.IV................G.....................I...............................I.............DN................... A....................

n=1(5a) ..................DR....A..... .N.VLH.....N....V.....S.IV................G.....................................................I.............DN................... A....................

n=1(16a) ..................DR.D..A..... .N.VLH.....N....V.....S.IV................G.....................................................I.............DN................... A....................

n=1(13a) ..................DR....A..... .N.VLH.....N....V.....S.IV................G.....................................................I.............DN................... A--------------------

**[B]**

n=4(4,7a,25ab) .............................. .N.AL.AVI....D.FGIITTFQFLAVSKI.F......II.EGIDGDGNPIFLMVCVVR..F....A.M...L...............GEQEENT..I.I.SR.Q.I....N.S...S.N.A.HTNN.VHA ...WELHETILS.FKI.A.EL

n=3(13b,14ef) ..................DR.......... .N.AL.AVI....D.FGIITTFQFLAVSKI.F......II.EGIDGDGNPIFLMVCVVR..F....A.M...L...............GEQEENT..I.I.SR.Q.I....N.S...S.N.A.HTNN.VHA ...WELHETILS.FKI.A.EL

n=2(12,14h) ET................DR.......... .N.AL.AVI....D.FGIITTFQFLAVSKI.F......II.EGIDGDGNPIFLMVCVVR..F....A.M...L...............GEQEENT..I.I.SR.Q.I....N.S...S.N.A.HTNN.VHA ...WELHETILS.FKI.A.EL

n=1(19c) ET..................T......... .N.AL.AVI....D.FGIITTFQFLAVSKI.F......II.EGIDGDGNPIFLMVCVVR..F....A.M...L...............GEQEENT..I.I.SR.Q.I....N.S...S.N.A.HTNN.VHA ...WELHETILS.FKI.A.EL

n=1(1a) ETL........................... .N.AL.AVI....D.FGIITTFQFLAVSKI.F......II.EGIDGDGNPIFLMVCVVR..F....A.M...L...............GEQEENT..I.I.SR.Q.I....N.S...S.N.A.HTNN.VHA ...WELHETILS.FKI.A.EL

n=1(19l) ET............................ .N.AL.AVI....D.FGIITTFQFLAVSKI.F......II.EGIDGDGNPIFLMVCVVR..F....A.M...L...............GEQEENT..I.I.SR.Q.I....N.S...S.N.A.HTNN.VHA ...WELHETILS.FKI.A.EL

n=1(14g) ET................DR.......... .N.AL.AVI....D.FGIITTFQFLAVSKI.F......II.EGIDGDGNPIFLMVCVVR..F....A.M...L...............GEQEENT..IEI.SR.Q.I....N.S...S.N.A.HTNN.VHA ...WELHETILS.FKI.A.EL

n=1(24a) ET............................ .N.AL.AVI....D.FGIITTFQFLAVSKI.F......II.E.IDGDGNPIFLMVCVVR..F....A.M...L...............GEQEENT..I.I.SR.Q.I....N.S...S.N.A.HTNN.VHA ...WELHETILS.FKI.A.EL

n=1(17e) ET..................T......... .N.TL.AVI....D.FGIITTFQFLAVSKI.F......II.EGIDGDGNPIFLMVCVVR..F....A.M...L...............GEQEENT..I.I.SR.Q.I....N.S...S.N.A.HTNN.VHA ...WELHETILS.FKI.A.EL

n=1(16b) ET............................ .N.AL.AVI....D.FGIITTFQFLAVSKI.F......II.EGIDGDGNPIFLMVCVVR..F....A.M...L...............GEQEENT..I.I.SR.Q.I....N.S...S.NFA.HTNN.VHA ...WELHETILS.FKI.A.EL

n=1(8h) ET............................ .N.AL.AVI....D.FGIITTFQFLAVSKI.F......II.EGIDGDGNPIFLMVCVVR..F....A.M...L...............GEQEENT..I.I.SRAQ.I....N.S...S.N.A.HTNN.VHA ....ELHETILS.FKI.A.EL

n=1(11) ..........................S... .N.AL.AVI....D.FGIITTFQFLAVSKI.F......II.EGIDGDGNPIFLMVCVVR..F....A.M...L...............GEQEENT..I.I.SR.Q.I....N.S...S.N.A.HTNN.VHA ..EWELHETILS.FKI.A.EL

**[C]**

n=1(20) ......................GT.L.... .NVAL..V.HKS..F..V.G.L...V...........E.L..G...D...IW.......KG.KD.Q....N.................I..E.NDE...I.S..E.....DN.SVV.CFN.A.HT..HVHI .I.W.F.RF...EQK...F.Q

n=1(8a) ..................DR...T.L.... .NVAL..V.HKS..F..V.G.L...V...........E.L..G...D...IW.......KG.KD.Q....N.................I..E.NDE...I.S..E.....DN.SVV.CFN.A.HT..HVHI ...W.F.RF...EQK...F.Q

n=3(8d-f) ETL...............DR..GT.L.... .NVAL..V.HKS..F..V.G.L...V...........E.L..G...D...IW.......KG.KD.Q....N.................I..E.NDE...I.S..E.....DNVSVV.CFN.A.HT..HVHI .I.W.F.RF...EQK...FYQ

n=2(25d,27) ETL...............DR..GT.L.F.. .NVAL..V.HKS..F..V.G.L...V...........E.L..G...D...IW.......KG.KD.Q....N.................I..E.NDE...I.S..E.....DNVSVV.CFN.A.HT..HVHI .I.W.F.RF...EQK...FYQ

n=2(19e,29) ETL...............DR..GT.L.F.. .NVAL..V.HKS..F..V.G.L...V...........E.L..G...D...IW.......KG.KD.Q....N.................G..E.NDE...I.S..E.....DNVSVV.CFN.A.HT..HVHI .I.W.F.RF...EQK...FYQ

n=1(24e) ..................DR..GT.L.... .NVAL..V.HKS..F..V.G.L...V......FKRTS---------------------------------------------------------------------------------------------- .I.W.F.RF...EQK...F.Q

**[D]**

n=3(7b,8g,14a) ..................DR....A....K .N.AL..V...D..F.......S.IV................G................K..KA.Q.GME.F.....S...K.......EYE.EA.....GS..QNIK.LDN.S.V.S.N.A.HT.N.VH. .....L.NI.RYA.YI*----

n=3(18,19a,28) ..................DR....A....K .N.AL..V...D..........S.IV................G................K..KA.Q.GME.F.....S...K.......EYE.EA.....GS..QNIK.LDN.S.V.S.N.A.HT.N.VH. .....L.NI.RYA.YI*----

n=1(14b) ...K........D.....DR....A....K .N.AL..V...D..F.......S.IV................G................K..KA.Q.GME.F.....S...K.......EYE.EA.....GS..QNIK.LDN.S.V.S.N.A.HT.N.VH. .....L.NI.RYA.YI*----

n=1(19m) ....D.............DR....A..... .N.AL..V...D..F.......S.IV................G................K..KA.Q.GME.F.....S...K.......EYE.EA.....GS..QNIK.LDN.S.V.S.N.A.HT.N.VH. .....L.NI.RYA.YI*----

n=1(3) ..................DR....A..... .N.AL..V...D..F.......S.IV................G................K..KA.Q.GME.F.....S...K.......EYE.EA.....GS..QNIK.LDN.S.V.S.N.A.HT.N.VH. .....L.NI.RYA.YI*----

n=1(15b) ...K..............DR....A....K .N.AL..V...D..F.......S.IV................G................K..KA.Q.GME.F.....S...K.......EYE.EA.....GS..QNIK.LDN.S.V.S.N.A.HT.N.VH. .....L.NI.RYA.YI*----

n=1(19d) ......R...........DR....A...VK .N.AL..V...D..F.......S.IV................G................K..KA.Q.GME.F.....S...K.......EYE.EA.....GS..QNIK.LDN.S.V.S.N.A.HT.N.VH. .....L.NI.RYA.YI*----

n=1(10b) ....D.............DR....A....K .N.AL..V...D..........S.IV................G................K..KA.Q.GME.F.....S...K.......EYE.EA.....GS..QNIK.LDN.S.V.S.N.A.HT.N.VH. .....L.NI.RYA.YI*----

n=1(24d) ..................DR....A....K .N.AL..V...D..........S.IV................G................K..KA.Q.GME.F.....S...K.......EYE.EA.....GS..QNIK.LDN.S.VGS.N.A.HT.N.VH. .....L.NI.RYA.YI*----

n=1(24c) ..................DR....A....K .N.AL..V...DA.F.......S.IV................G................K..KA.Q.GME.F.....S...K.......EYE.EA.....GS..QNIK.LDN.S.V.S.N.A.HT.N.VH. .....L.NI.RYA.YI*----

n=1(22) ETL...............DR....A....K .N.AL..V...D..........S.IV................G................K..KA.Q.GME.FLNMNKVFNQRLFAISS------------------------------------------- .....L.NI.RYA.YI*----

n=1(19b) ..................DR....A....K .N.AL..V...D..F.......S.IV....D...........G................K..KA.Q.GME.F.....S...K.......EYE.EA.....GS..QNIK.LDN.S.V.S.N.A.HT.N.VH. .....L.NI.RYA.YIQE...

n=1(10a) ..................DR....T....K TN.AL..V...D..........S.IV................G................K..KA.Q.GME.F.....S...K.......EYE.EA.....GS..QNIK.LDN.S.V.S.N.A.HT.N.VH. ...W.L.NI.RYA.YI*----

**Hybrid/mosaic**

n=2(23a,24f) ET.....................T.L.... .NVAL..V.HKS..F..V....S.IV................G.....................................................I.................................. .....................

n=1(25c) ETL...............DR..GT.L.... .NVAL..V.HKS..F..V....S.IV................G.....................................................I.................................. .....................

n=1(5b) ......................GT.L.... .NVAL..V.HKS..F..V....S.IV...I............G.....................................................I.................................. .....................

n=1(14c) ET.....................T.L.... .NVAL..V.HKS..F..V....S.IV................G.....................................................I....................D...A.HT.N.VH. .....L.NI.RYA.YI*----

n=1(19f) ET................DR..GT.L.... .NVAL..V.HKS..F..V.G.L...V..T........E.L..G...D...IW.......KG.KD.Q....N.................I..E.NDE...I.S..E.IK.LDN.SVV.C.N.A.HT..HVHI .I.W.F.RF...EQK...F.Q

n=1(19g) ET................DR..GT.L.... .NVAL..V.HKS..F..V.G.L...V..T........E.L..G...D...IW.......KG.KD.Q....N.................I..E.NDE...I.S..E.IK.LDN.S.VGS.N.A.HT.N.VH. .....L.NI.RYA.YI*----

n=1(17c) ..................DR....AL.... .NVAL..V.HKS..F..V.G.L...V...........E.L..G...D..................Q....N.................I..E.NDE...I.S..E.IK...N.SVV.C.N.A.HT.N.VHA .I.W.F.RF...EQK...F.Q

n=2(19hj) ..................DR....A....K .N.AL..V...D..F.......S.IV................G................K..KAAQ.GME.F.....S...K.......EYE.EA.....GS..QNIKFLDN.S.V.S.N.A.HT.N.VH. ...WELHETILS.FKI.A.EL

n=1(17d) ..................DR....A....K .N.AL..V...D..F.......S.IV................G................K..KAAQ.GME.F.....S...K.......EYE.EA.....GS..QNIKFLDN.S.V.S.N.A.HT.N.VH. A.....HETILS.FKI.A.EL

n=1(24b) ET................DR.......... .N.VLH.....N....V.....S.IV..............A.G.....................................................................................VHA ...WELHETILS.FKI.A.EL

n=1(8b) ..................DR....A....K .N.AL..V...D...FGIITTFQFLAVSKI.F......II.EGIDGDGNPIFLMVCVVR..F....A.M...L...............GEQEENT..I.I.SR.Q.I....N.S...S.N.A.HTNN.VHA ...WELHETILS.FKI.A.EL

n=1(8c) ..................DR....A....K .N.AL..V...D...FGIITTFQFLAVSKI.F......II.EGIDGDGNPIFLMVCVVR..F....A.M...L...............GEQEENT..I.I.SR.Q.....DN.SVV.CFN.A.HT..HVHI .I.W.F.RF...EQK...F.Q

n=1(21) ET............................ .N.AL.AVI....D.FGIITTFQFLAVSKI.F......II.EGIDGDGNPIFLMVCVVR..F....A.M...L...............GEQEENT......................S.N.A.HT.N.VHA ...WELHETILS.FKI.A.EL

n=1(19i) ..................DR.......... .N.AL.AVI....D.FGIITTFQFLAVSKI.F......II.EGIDGDGNPIFLMVCVV.K..KA.Q.GME.F.....S...K.......EYE.EA.....GS..QNIKFLDN.S.V.S.N.A.HT.N.VH. ...WELHETILS.FKI.A.EL

n=1(14d) ET...ISYSWILRFEMRR------------ .N.TL.AVI....D.FGIITTFQFLAVSKI.F......II.EGIDGDGNPIFLMVCVVR..F....A.M...L...............GEQEENTA.I.I.SR.Q.I..LDN.S.V.S.N.A.HT.N.VH. .....L.NI.RYA.YI*----
