# Supplementary material for: Genomic analyses of pneumococci reveal a wide diversity of bacteriocins – including pneumocyclicin, a novel circular bacteriocin
Source: BMC Genomics. 2015 Jul 28;16(1):554. doi: 10.1186/s12864-015-1729-4 (PMC4517551; doi:10.1186/s12864-015-1729-4)
Supplement: Additional file 4: — Alignment S3. Nucleotide sequence differences between blp Groups within each Category. [file 12864_2015_1729_MOESM4_ESM.docx]

**Alignment S3. Nucleotide sequence differences between *blp* Groups within each Category.**

These alignments consist of two parts: firstly, nucleotides variable among the Groups, and secondly, nucleotides with coverage of <100% among Groups (i.e. showing insertions and deletions). Neighbour-joining trees calculated in MEGA5 are included for any Category with at least 3 Groups.

Identical nucleotides are represented by a dot (.) and absent nucleotides by a dash (-).

Insertion sequence (IS) nucleotide sequences are represented by Ns, except for their first and last 25 nucleotides for which the original sequence was kept (see Methods section in main text). Any alignment rows consisting solely of continuing IS dummy sequence have been removed and replaced by [...IS...].

**Category 1**

**Variable nucleotides**

[ 111111 1111111111 1111111111 1111111111 1111111111 1111111111 1111111111 1111111111 1111111222 2222222222 2222222222 2222222222 ]

[ 155577888 8899000000 0000000116 6777777777 7777778888 8888888888 8888888888 8888888888 8889999999 9999999000 0000000000 0000000000 0000000000 ]

[ 8305767026 7813122344 4556677458 9000111223 3469991222 2233333344 4445555556 6666777777 8990011145 5788899003 4444445555 5566666666 7777888999 ]

[ 5797099920 8645735701 4362815357 6359078073 7482587235 8913457845 6890345792 3469123679 2172623595 7247809585 0123482356 7801234789 0167036014 ]

#1a GAGGCACCGT CATACGACTG TATTATTGTG GACGCATGGG TAACTACATT TCAGGACAAC GCTTCAATTG CTTGAAATTG CATTTTCAAA TCAAGGCTTG GAATTGGGAT GGAATCCTCT TTTGTGAGTG

#1b AGATTGTTTC TGAGTACTCT CGACGCCAAA ATTTTTAAAA CGGTGGTGAA GTGCCTTGGT ATGAGGCAAA TAGAGTTCCA TGCACCTGCT CTGGATTACA AGCAATATGC TATCATTCTG GGGTAATAAT

[ 2222222222 2222222222 2222222222 2222222222 2222222222 2222222222 2222222222 2222222222 2333333333 3333333333 3333333333 3333333333 3333333333 ]

[ 1111111111 1222222222 3333333333 3334444444 4455555555 5666666677 7778888888 9999999999 9000000000 0111111111 1111111111 1111111145 5567777888 ]

[ 0000023458 8001122345 0025566667 8894447788 9933455669 9011268912 5780233457 0022567779 9123357899 9000011112 2223333444 4444556640 0823448002 ]

[ 1235957362 5125917634 5823925672 4554670925 2716107130 4614663592 8366069512 1269032682 6630200014 7013505891 5671579012 3457344574 5130140472 ]

#1a AGTTTACATT TGCTCAGACA ACGATGGGAA ACCATCTACC TCACCGCCGG AATCGATGCA CTACATTTCC CAATACAACC AAGAGCTGCA ATATCGTGAT AATCAATGTT TCAGGTCAGA ACTTTCGAAT

#1b TTCCCGTTAC GATCTGAGTG CATCCTTAGG GTTGAACGAT CTGTTATAAT CGGTAGCAAG TGGTGCCCTA TTTCGTGGTT GGACATAATT TATAGCCATG TGCATTAAGG CTGATAAGAG GACCCTATGC

[ 3334444444 4444444444 4444444455 5556667777 7777777777 7777777777 7777777777 7777777777 88889]

[ 8990000011 2233334445 5556789900 1122565555 5555556667 7777777777 7777888888 8888889999 13560]

[ 4790123757 8823570196 7899033334 0823410344 5566892550 0111345667 7888000122 5778990358 08614]

[ 8231497913 3554117399 8736513695 1708763945 8916436161 4123768253 6018039847 1287892960 62065]

#1a CCTCCTTGCC CTAATCACCC CGTGCTAGTT AGTCAACAGC TGAGCTTCTT GAACCTCGAT CACAGTGTCC CCGTTGTTCT ACCAC

#1b TAATTCCAAT TGGGGTCTTT AACTTCGTCC GACTGCTTTT GTGTTCGTGG AGCTTATAGC TGTGACAGTT TTACCACGTC GTTCT

**Nucleotides with coverage <100%**

[ 1333]

[ 46000]

[ 19448]

[ 24231]

#1a TG---

#1b --GAT

**Category 5**

**Variable nucleotides**

[ 1 1111111111 1111111111 1111111111 1111111111 1111111111 1112222233 3333344444 4444444444 4444444444 5555555555 5566666666 6677777]

[ 1566778990 0111111222 2223333344 4445555556 6666777777 7777777778 8882266725 8889900000 0001111122 2333444567 0000013588 8912224466 6700111]

[ 8712277168 8088889003 6671345904 4440122680 0389012233 4444578891 3399966518 0028901235 5672355935 6057368997 2346900788 9353672402 2738122]

[ 9029098493 6424581036 0421979540 1371347942 8274600439 1458912629 3516926891 4728314970 6408118064 5511971061 3952213516 6212244182 9599517]

#5a CCTGGCCTGT ACTGGTTGTC TCTAATGCAT CCGTTACAGC TGAGATACAA TGCGTGATCG TTGGCTATCA AATCTCCTTT GTCAACGTGT CAGTACCTGG ATTAGAACGG GCAGAGAGAA CCTGTTA

#5b TTCAATTAAA GAAAACAACT ATCTGCATGC AACCCGTCAG CAGTGCGTGC CATAAAGCTT CGACTAGCTC TGCTATTCCA TCTGGTAAAC TGTCGTAGAA GCCGAGGTAT ATGAGTCTCG TTAAAGG

**Nucleotides with coverage <100%**

[ 6666666666 6666666666 6666666666 6666667]

[ 3333999999 9999999999 9999999999 9999991]

[ 5555666666 6666777777 7777888888 8888991]

[ 5678012345 6789012345 6789012345 6789014]

#5a ----TAGAGG TGGCTTGAAA TAGAGGTGGC TTGAAA-

#5b GCTT------ ---------- ---------- ------A

**Category 7**

**Variable nucleotides**

[ 1111 1111111111 1111111111 1111111111 1111111111 1111111111 1111111111 1111111111 1111111111 1111111122 2222222222 2222222222 ]

[ 125667778 9999990011 1122222222 3444444455 6666666777 7777778888 8888888888 8888888888 8888888888 8889999999 9999999900 0000000000 0000000000 ]

[ 1857122477 1233468888 8800346889 1001344424 0336779235 6788991122 2223333334 4445555556 6666777777 8990011234 5578889900 3444445555 5566666666 ]

[ 9990290198 4935593624 5803620570 1143703745 2288176779 8126587923 5891345784 5890345791 3469123679 2172623709 5724780958 5123482356 7801234789 ]

#7a CTATCAACTT AGGGGAAGAA ACACTCACTG TCACACACAT CGGTTCGGTT AAGCTACGAT TTCAGGACAA CCTTCAATTG CTTGAAATTG CATTTTCGAA ATCAAGGCTT GAATTGGGAT GGAATCCTCT

#7b TCGCTGGACC TACAAGTATG GTGTCTTTGA GTGTGGCGGC GACCCAAACC GGATGGTTGA AGTGCCTTGG TTGAAGCAAA TAGAGTTCCA TGCACCTAGC TCTGGATTAC AGCAATATGC TATCATTCTG

[ 2222222222 2222222222 2222222222 2222222222 2222222222 2222222222 2222222222 2222222222 2222222222 2222222222 2233333333 3333333333 3333333333 ]

[ 0000000000 1111111111 1111111112 2222222222 2222333333 3333334444 4444455555 5666666666 6666677777 7788999999 9900000000 0111111111 1111111111 ]

[ 7777888999 0000012334 5566678880 0011122344 4579256666 6778893446 6778933345 7011112233 4456801777 8834045667 8934467999 9000012222 2333334555 ]

[ 0167036014 1235905173 1616902591 2912548612 3816290267 9244751464 7092216820 3245670628 1862379389 2565240286 6222818036 9024930578 9236798245 ]

#7a TTTGTGAGTG AGTTTCATCA GTACGTTTAG CGCTTGCAAC CGCGGTCGGA CACATCAATC CTACTACAGG GGCCGTTGCT AATATTCTCA GCTTATATCA TCGACTGCAA TATCGTTAAT TCTAATGGGT

#7b GGGTAATAAT TTCCCTGCTC AACACCCGCA TTGCCCGGTT TAACTCTTAG TGAGCTGGAT TCGTCGTGCA AATAAACATA GTCTCCTCTG ATCCCCGCTG CTAGTAATTT ATAGTAGGGC ATCTTAATAA

[ 1111111111 ]

[ 3333333333 3333333333 3334444444 4444444444 4444455555 5555555666 6666666666 6666666677 7777899999 9999999999 9999999999 9999999999 0000000000 ]

[ 1334455567 7777778888 9990000000 0144555677 7889900011 3589999344 4556677788 9999999900 0008602455 5777777777 8888888999 9999999999 0000000011 ]

[ 6150400821 1334890012 2890133556 7268001902 7350412639 5732333114 6050877867 0456778803 3479225925 5122334467 3348899123 4455666789 0355677813 ]

[ 5049856242 4365105843 5942508175 1582079764 2209954321 2674023102 8339417653 9799120882 3216790161 4945891643 1611413768 2536018798 7128789296 ]

#7a CGCAGAACTA CTGCGGAATT ACTCCTTTGT CGCCTCATTA ATCCTAAGTA CCGTGTCGGG GCTATAGACA GCAGTTGCAT TCGGCCTATG GTTTGTGTTC TGCGAGTTAT AGCTGTGAAG TTTACCACGT

#7b ATTGTGGACG ACAGAATGCC CTATTCCATC TATACTGACG GCTACGGAGG TTACTCTATT ATCCCGACTC ATCTGCATGA ATATTTCGCA AAGCTGAGCT ATTTGACCTC GATCACAGGT CCCGTTGTTC

[ 111111]

[ 000000]

[ 113467]

[ 683926]

[ 074287]

#7a CGCGTG

#7b TATAAT

**Nucleotides with coverage <100%**

[ 11111 1111111111 1111111111 1111111111 1111111111 1111111111 1111111111 1111111111 1111111111 1111111111 1111111111 1]

[ 333333666 6666700000 0000000000 0000000000 0000000000 0000000000 0000000000 0000000000 0000000000 0000000000 0000000000 0000000000 0]

[ 4001111333 3999044444 4555555555 5555555555 5555555555 5555555555 5555555555 5555555555 5555555555 5555555555 5555555555 5555555555 5]

[ 1782244555 5557399999 9000000000 0111111111 1222222222 2333333333 3444444444 4555555555 5666666666 6777777777 7888888888 8999999999 9]

[ 2902345678 9459645678 9012345678 9012345678 9012345678 9012345678 9012345678 9012345678 9012345678 9012345678 9012345678 9012345678 9]

#7a T----TC--- -CA-A----- ---------- ---------- ---------- ---------- ---------- ---------- ---------- ---------- ---------- ---------- -

#7b -TTAA--GCT T--T-TAATA CTCAATGAAA ATCAAAGAGC AAACTAGGAA ACTAGCCGCA GGCTGTACTT GAGTACGGCA AGGCGACGTT GACGTGGTTT GAATTTGATT TTCGAAGAGT A

**Category 8**

**Variable nucleotides**

[ 11111 1111111111 1111111111 1111111111 1111111111 1111111111 1111111111 1111111111 ]

[ 11222233 3333333444 4444444555 5555556666 7777778889 9999900000 0000000000 1111111122 2222222223 3333344444 4455555556 6666667777 7777777777 ]

[ 1855346913 3466799000 1112228002 3566771356 0022371263 3347912234 4455667788 2458888900 3466778891 2446900344 4401224780 3367790023 4444667888 ]

[ 9246488832 7445026289 2890147490 1846123054 2613900310 4660484681 2547392647 6463569214 7315036812 4080625812 4824586053 3992876788 0269092237 ]

#8a TGTGGCGAGG GTACGTTCAT TTTTACTGGC TTGCCCCAAA CCAACGTGCG GGGAATACTC TCGACGCCAG CGGTGGCTAC TCATCCCTGT CATGCCAGCA ACCCGTTCAG AGTTCGTGAC CCTAAGAAGC

#8b .......... .......... ........A. ......TG.. ..G....T.. ...G...... ........TA TAA...T.GT CTT...TGAG .....TG.GC CGTT.CCAG. .CCCAACA.. ATCGC.GGAT

#8c .......... .......... ..-.....A. ......TG.. ..G....T.. ...G...... ........TA TAA...T.GT CTT...TGAG .....TG.GC CGTT.CCAG. .CCCAACA.. ATCGC.GGAT

#8d C.CA.GACAA TCGAACC.TC CCGCCTGAAT C.TTTT..GG TAGGGAC.T. .A...CGACT GTATTATT.. T.T...TGGT C.TCTT.... .GCAT.G... .......... .......... ..........

#8e C.CA.GACAA TCGAACC.TC CCGCCTGAAT C.TTTT..GG TAGGGAC.T. .A...CGACT GTATTATT.. T.T...TGGT C.TCTT.... .GCAT.G... .......... .......... ..........

#8f C.CA.GACAA TCGAACC.TC CCGCCTGAAT C.TTTT..GG TAGGGAC.T. .A...CGACT GTATTATT.. T.T...TGGT C.TCTT.... .GCAT.G... .......... .......... ..........

#8g .......... .......... ........A. ......TG.. ..G....T.. ...G...... ........TA TAA...T.GT CTT...TGAG .....TG.GC CGTT.CCAG. .CCCAACA.. ATCGC.GGAT

#8h .A..A..... .......T.. ........A. CG.....GG. ..G....T.A CAAGGCGAC. C....A.... T..AAA.CGT C.TC.T...A T....TGA.C ..TTAC.AGC G.....CAGT ATCGTA.G..

[ 1111111111 1111111111 1111111111 1111111111 1111111111 1111111112 2222222222 2222222222 2222222222 2222222222 2222222222 2222222222 2222222222 ]

[ 7778888888 8888888888 8888888888 8888888888 8999999999 9999999990 0000000000 0000000000 0000000000 0011111111 1111111111 1122222222 2222222222 ]

[ 9991222223 3333334444 5555556666 6777777889 9001123335 5557888990 0034444455 5555666666 6777778889 9900001123 3455566778 8900111122 2223344455 ]

[ 3698034690 2456895679 0145680245 7023478032 8373481670 2683589140 6962345934 6789123458 9012781471 2523460162 8425727013 6023023602 5893723489 ]

#8a TGGTTGAAGT GCCGTGGGAT GCGGCAAGTA GAGTTCCATG CACCTGAAAC TCCTAGATAT TTAGCAATAT ACTATCATTT TTGGGTAATA ATTTCCCTGT CCAGACAATC GCATGCTCTG GACAGACTGG

#8b C..CGATTTC AGGACAACGC TTCAATT.CT TGAAATTGCA TTTTC..GGA CATC.AGGCC ..GAATTGGG .TGGAATCC. C.TTTGTGAG TGAGTTTCA. .AGATACG.T TAGC...TCA .G.GA..CA.

#8c C..CGATTTC AGGACAACGC TTCAATT.CT TGAAATTGCA TTTTC..GGA CATC.AGGCC ..GAATTGGG .TGGAATCC. C.TTTGTGAG TGAGTTTCA. .AGATACG.T TAGC...TCA .G.GA..CA.

#8d .......... .......... .......... .......... .......... C.......C. .......... .......... .......... .......... .......... .......... ..........

#8e .......... .......... .......... .......... .......... C.......C. .......... .......... .......... .......... .......... .......... ..........

#8f .......... .......... .......... .......... .......... C.......C. .......... .......... .......... .......... .......... .......... ..........

#8g C......... ...T...TG. .AA....A.. .......... .....AGGG. CT..G...C. AC........ G........C .G........ .........C T..A...CC. ....TGC.CA CGGG.TT.AA

#8h CTACGATTTC AGGACAACGC TTCAATT.CT TGAAATTGCA TTTTC..GGA CATC.AGGCC ..GAATTGGG .TGGAATCC. C.TTTGTGAG TGAGTTTCA. .AGATACG.T TAGC...TCA .G.GA..CA.

[ 2222222222 2222222222 2222222222 2222222222 2222222222 2222222222 2222222222 2222222222 2222222223 3333333333 3333333333 3333333333 3333333333 ]

[ 2223333333 3333333334 4444444444 4444555555 5555556666 6666666666 6677777777 7778888889 9999999990 0000000000 0000011111 1111111111 1111111111 ]

[ 6790225666 6677788890 0133445566 7889333455 5667990011 1112334456 8901236778 8880134570 4566788990 0233445666 7899900000 1112222222 2333333444 ]

[ 1276234013 7803556862 8128573958 1033279314 8244153725 6787392973 4385092490 3677676203 5139717372 5634340359 3025812469 1590245678 9023568012 ]

#8a GCCGATCCCT AGCACGATCG GGGTGATCCC CGTCACAGAA TGAGTCGGGT CGTACTAATT CACATGCTTA ACTTTCTTAA TATCGCCCGC TAATGATGGC TGTTTCTAGA CTATTTTTGT TCATATTAAA

#8b T.GAGGAT.G GA...AC..T ACACAT.... TACT....G. CCG.GA.ATC ...G.....A TTT.CAT.C. G.ACAT.CC. ....AAT.AA CGGCT.CTTT C.CAATATCG G.GA..AAA. .TC..AATGT

#8c T.GAGGAT.G GA...AC..T ACACAT.... TACT....G. CCG....... .......... ..A....... .......... .......... .......... .......... .......... ..........

#8d .......... .......... .......... .......... .......... .......... ..AG...... .......... .......... .......... ........A. .......... ..........

#8e .......... .......... .......... .......... .......... .......... ..AG...... .......... .......... .......... ........A. .......... ..........

#8f .......... .......... .......... .......... .......... .......... ..AG...... .......... .......... .......... ........A. .......... ..........

#8g TA.AG.A.T. ..TGA.CCTT AC.C..CTTT ....GTGC.. CCGAGAAAT. AAA.TAGTC. .T...ATC.G .TACA.CCCC CGCT.A.TAA CG.CTG.ATT CA...A...G TAGAGAA..A G.TAC....T

#8h T.GAGGAT.G GA...AC..T ACACAT.... TACT....GC CCG.GA.ATC ...G.....A TTT.CAT.C. G.ACAT.CC. ....AAT.AA CGGCT.CTTT C.CAATATCG G.GA..AAA. .TC..AATGT

[ 3333333333 3333333333 3333333333 3333333333 3333333444 4444444444 4444444444 4444444444 4444444444 4444444444 4555555555 5555555555 5566666666 ]

[ 1111111111 3444455555 5556777777 7777888888 8999999000 0000001111 1112223333 3334444444 5555555566 7788889999 9000000111 1234578889 9901112222 ]

[ 4444555667 5048800666 8892112333 4489001245 8023789013 3556782235 7793560023 3570146788 0017789939 2723580334 7234689039 9096773892 3321253467 ]

[ 3458245567 4981256458 0224247136 2510584399 6050394250 8175105922 4717563665 6228478702 0790981467 4292099479 5456373221 8797697774 0300223535 ]

#8a TCTAGAACAG CAGTGAAGAT ACATACGTTG TCGGAATTCA ATACCCTCCT TTGTCGGAAC CATGTCCAAA TTCACACATC CCACCGTTGG AGGTCAAAGC CGCTAGGAGG GCACTCGGGT GCCGGAGTAG

#8b .TCG.GT... ..A..GG... .A........ ..A.TG.CT. GGC..TATTC CATC...GGT T.AACT.GGG C......... .T..AA.C.. .A.C...G.. .......... ...T.T.TAC T...-G....

#8c .........A TGT..GG... .A.C..A... ..A.TG.CT. ..C..TATTC CATC...GGT T.A...G.GG .GTCT.T..A .TGTAA.C.A G..CT..... .......... .......... ....-G....

#8d .........A TG.ACGGAGG TA.C..A... ..A.TG.CT. ...T..ATTC C....A.GGT T.A.CT.GGG .GT....... TT.TAAGCA. .A....C..T G.ACG.AG.A ....C..... .......C..

#8e .........A TG.ACGGAGG TA.C..A... ..A.TG.CT. ...T..ATTC C....A.GGT T.A.CT.GGG .GT....... TT.TAAGCA. .A....C..T ..ACG.AG.A ....C..... .......C..

#8f .........A TG.ACGGAGG TA.C..A... ..A.TG.CT. ...T..ATTC C....A.GGT T.A.CT.GGG .GT....... .T.TAAGCA. .A....C..T ..ACG.AG.A ....C..... .......C..

#8g GT..T..A.. TGT..GG... .A.CGA..CA .GAATGCCTG ....A....C C....A...A TG......GG .GTCTTTGG. .T.TAAGC.A ..AC.GCGTT .AACGA.GT. ATG.C..TA. .TTAAGACGT

#8h .TCG.GTAG. ..A..GG... .AGC...C.. CTA.T..CT. ..C..TATTC CATCT.A... ........GG .GTCT.T..A .TGTAA.C.A G..CT..... .......... ......A..C TT.....C..

[ 6666666666 6666666666 6666666666 6666666667 7777777777 7777777777 7777778888 8888888888 8888888888 8888888888 8888888888 8888889999 9999999999 ]

[ 4445566677 7888888888 8899999999 9999999990 0000000000 0111222223 3457890011 3355555566 6666666666 6666777777 8888889999 9999990000 0000000000 ]

[ 1460503877 8455677788 8901345555 7777788891 2334444668 9157001472 6266044599 2535778801 3333667788 8999011235 0155570023 5677890001 1122467899 ]

[ 0283390417 6957534713 4093472349 0256906830 0282347095 2039366085 6847479946 8964342464 0357565724 6048914322 7107903949 4525292385 6779132814 ]

#8a GGGCTATTAG CGGCCATGAA TGGCGCTTCA GTTGAGGCCA TTCTAGCGGT TTGCTCATGC CCTCCTGTCC TCGTATATAG TTATGAATAA TATTCGTTTT CGCAGTTCCT GGCCACTACA CCCGAACATC

#8b ..ATCC.C.. .A.A...... .T..A...T. ..CAT..T.T C.ACGAA.A. .CA.CAGCAA TTG..C..TT C......... .......... ......G... .....CCT.. T.TTCT..T. T..ATGAGGT

#8c ..ATCC.C.. .A.A...... .T..A...T. ..CAT..T.T C.ACGAA.A. .CA.CAGCAA TTG..C..TT C......... .......... ......G... .....CCT.. T.TTCT..T. T..ATGAGGT

#8d ..ATC.CC.. A.C.A.CTGG CA.T..AA-. A..ATA.TT. .C........ .......... ..G..CACTT .......... .......... .......... .......... .......... ..........

#8e ..ATC.CC.. ..C.A.CTGG CA.T..AA-. A..ATA.TT. .C........ .......... ..G..CACTT .......... .......... .......... .......... .......... ..........

#8f ..ATC.CC.. A.C.A.CTGG CA.T..AA-. A..ATA.TT. .C........ .......... ..G..C.CTT .......... .......... .......... .......... .......... ..........

#8g TTATCC.CGA ....TC.... ..A..T..-C .CGATACT.. C.ACGAAAAA GCATCAG... ..GTTC.CTT .GAAGCCGGA GACAACCACG ACCCTA.CCC TAAGA.CTTA TC.T..GG.C TTT.TGAGGT

#8h TTATCC.CGA ....TC.... ..A..T..-C .CGATA.T.. C.ACGAAAAA GCATCAG... ..GTTC.CTT .GAAGCCGGA GACAACCACG ACCCTA.CCC TAAGA.CTTA TC.T..GG.C TTT.TGAGGT

[ 1111111111 1111111111 1111111111 1111111111 1111111111 1111111111 1111111111 1111111111 1111111111 1111111111 1111111111 ]

[ 9999999999 9999999999 0000000000 0000000000 0000000000 0000000000 0000000000 0000000000 0000000000 0000000000 0000111111 1111111111 1111111111 ]

[ 0111111112 3333333456 1111222222 2222222223 3333333344 4444555555 5555555555 5555566666 6666666667 7777777777 7788000112 2222223333 3345555666 ]

[ 9223334451 3556678692 0268011123 4455788890 0012349924 5689001111 2222344445 6778901134 4455678880 0235667777 8955255890 0237990366 7883338001 ]

[ 6591570700 0672722749 7449123519 5845014903 4613800616 8721370238 1256323584 9258664723 4707824591 2545570369 8513568941 8574086323 6540586470 ]

#8a TCGCTGCGAT TGTCCCTCTG CCCACCGCCT ACATAATTTG TGTCCGCTCG TTTATAGCCC TCGTGATGTA TTCCTCTTCC ACGCCGGTAT GCATGAGCCG CCGCATACAA TTATCTCGCT TGATGCACCC

#8b CTAACAAAC. .AATTTC... .T.G...... .......... ......G... .......... .......... .........T GT........ ...C...... ....TC.TGG CG.CAC.AA. ATGGATGGAT

#8c CTAACAAAC. .AATTTC... T..G...... .......... ......G... .......... .......... .......... .......... ...C...... .......... .......... ......G...

#8d .........C .AATTTCTCA ...G....TC TTCG...... ......G... .......... .....G.AC. .......... .......... ...C...... .......... ......TA.. ......G...

#8e .........C .AATTTCTCA ...G....TC TTCG...... ......G... .......... .....G.AC. .......... .......... ...C...... ......G.T. ......TA.. ......G...

#8f .......... C......... ..TGATATTC T.CGTGAGCT GAGTTAGC.T .CCTC.ATT. ..ACTGCAC. CCTAC.GAT. ...T.ATCC. A.TCTCAAAA GTAT...... CGG....... ......G...

#8g CTAACAAACC C......... ...G....TC TTCG..AGCT GAG.T..CTT CCCTCGA..T CTACAG...G CC..CTGCT. ..ATT.T..C .T.C...... ......G... .......AAC ..G...G...

#8h CTAACAAACC C......... ...G....TC TTCG..AGCT GAG.T..CTT CCCTCGA..T CTACAG...G CC..CTGCT. ..ATT.T..C .T.C...... ......G... .......AA. ..G...G...

[ 1111111]

[ 1111111]

[ 7777888]

[ 5689113]

[ 4741489]

#8a CCCTCTC

#8b TT.CTCT

#8c TTTCTCT

#8d ....TCT

#8e ....TCT

#8f TTTC...

#8g .......

#8h .......

**Nucleotides with coverage <100%**

[ 11 1111111111 1111111111 1111111111 1111111111 1111111111 1111111111 ]

[ 33336666 6666666666 6666666666 6666666666 6667788888 8888888888 8888888801 1111111111 1111111111 1111111111 1111111111 1111111111 1111111111 ]

[ 4500001111 1111111111 1111111111 1111333399 9990036667 7777777777 7777777780 0000000000 0000000000 0000000000 0000000000 1111111111 1111111111 ]

[ 1146881111 1112222222 2223333333 3334555535 5689964445 5666666666 6777777712 6666666666 7777777777 8888888888 9999999999 0000000000 1111111111 ]

[ 9117123456 7890123456 7890123456 7890678924 5911907898 9012345678 9012345647 0123456789 0123456789 0123456789 0123456789 0123456789 0123456789 ]

#8a T-A-TTTAAT CTCTGGCTCA GAACGGTTAG GAGA----GC A-A-GT---- ---------- --------A- TAATACTCAA TGAAAATCAA AGAGCAAACT AGGAAACTAG CCGCAGGCTG TACTTGAGTA

#8b .--G------ ---------- ---------- ---------T .T---.---- ---------- --------.- ---------- ---------- ---------- ---------- ---------- ----------

#8c --.-..---- ---------- ---------- ---------T .T---.---- ---------- ---------- .......... .......... .......... .......... .......... ..........

#8d GA.-...... .......... .......... ....----.- .T.-.----- ---------- --------.- .......... .......... .......... .......... .......... ..........

#8e GA.-...... .......... .......... ....----.- .T.-.----- ---------- --------.- ---------- ---------- ---------- ---------- ---------- ----------

#8f GA.-...... .......... .......... ....----.- .T.-.----- ---------- --------.A .......... .......... .......... .......... .......... ..........

#8g .--G...... .....A.... .......... ....GCTT.- -T.A..AGCA GTTTTTAAAT TTTTCCTT.- ---------- ---------- ---------- ---------- ---------- ----------

#8h .A-G--.... .......... .......... ....GCTT.- -T.A..AGCA GTTTTTAAAT TTTTCCTT.- ---------- ---------- ---------- ---------- ---------- ----------

[ 1111111111 1111111111 1111111111 1111111111 111111]

[ 1111111111 1111111111 1111111111 1111111111 111111]

[ 1111111111 1111111111 1111111111 1111111111 111111]

[ 2222222222 3333333333 4444444444 5555555555 666666]

[ 0123456789 0123456789 0123456789 0123456789 012345]

#8a CGGCAAGGCG ACGTTGACGT GGTTTGAATT TGATTTTCGA AGAGTA

#8b ---------- ---------- ---------- ---------- ------

#8c .......... .......... .......... .......... ......

#8d .......... .......... .......... .......... ......

#8e ---------- ---------- ---------- ---------- ------

#8f .......... .......... .......... .......... ......

#8g ---------- ---------- ---------- ---------- ------

#8h ---------- ---------- ---------- ---------- ------

**Category 10**

**Variable nucleotides**

[ 111111 ]

[ 111113 3333333333 3333333333 3334444444 4444444444 4444444444 4555555555 6666666666 6666667777 7788888888 8888000000 ]

[ 1122233333 3333444444 4466124671 1334455567 7777778888 8890001112 3333444444 5555567888 9000023589 0144668899 9999990000 0044455555 5555000000 ]

[ 5546913346 6799001112 2215054806 8050400821 1334880012 4572375778 2357014677 0678997238 4233623733 8604025645 5567791456 8917711222 5677136788 ]

[ 4688832744 5026892890 1435428824 4938745131 3254094732 8829791365 5411736769 6987361818 8349204562 4291896462 3780318187 4083536356 2712792914 ]

#10a TGCGAGGGTA CGTTATTTTT ACTACTATCC GGCAGAACTA CTGCGGAATT CACTTGCCAG AATCACACAT CCCGTGAGTG TAATGTGCAC ATGGACACCA AAATTTTAGG TTGTGGTGTC AGGCCCGACA

#10b CAGACAATCG AACCTCCCGC CTCGACGGTA ATTGTGGACG ACAGAATGCC TGACCAATGT GGGTCTTTGG TTAACAGACA CGCCACATGT GCTTCTCTTT TCGCGCCGAA AGAGATCACT TAATTTAGTG

[ 1111111111 1111111111 1111111111 1111111111 1111111111 1111111111 1111111111 1111111111 111]

[ 0000000000 0000000000 0000000000 0000000000 0000000000 0000000000 0001111111 1111111111 222]

[ 0011111112 2222233334 4444555566 6677777777 7788888888 8889999999 9990000000 0011144566 000]

[ 8923558890 3345900883 7888359904 7812223788 8901112345 8891112455 7790333444 5622779003 235]

[ 8463792844 5689759583 0124902890 0285676612 7022473247 3622366148 0143469258 7402763510 252]

#10a AATATTTCAC AGCTGTTGGC CCGCAACCGT TCGAACTGCC TCATGTGTCC TCGTGTCGTA CGTAGAGCCG CCGCTTTAAA CCC

#10b GGCGGACTGA GTGCTCCAAT ATATTGTTAG GTAGCTCATT CTTCACAGTA GAACACTACC TACTTCAAAA GTATCCGGGC TTT

**Nucleotides with coverage <100%**

[ 11 111111]

[ 666667800 000000]

[ 5333390311 111111]

[ 1555558500 000002]

[ 1567813823 456781]

#10a -----A---- ------

#10b AGCTT-ATAT TAAACA

**Category 13**

**Variable nucleotides**

[ 1111111111 1111111111 1111111111 1111111111 1111111111 1111111111 1111111111 1111111111 1111122222 2222222222 2222222222 2222222222 2222222222 ]

[ 2222223444 4444456667 7777777777 7777888888 8888888888 8888888888 8888888888 8999999999 9999900000 0000000000 0000000000 0000000011 1111111112 ]

[ 4678891134 4478910280 1222334678 8899122222 3333334444 4555555666 6677777789 9001114557 8889900344 4445555556 6666666677 7788899900 0002345880 ]

[ 2425701370 3765458675 0047374811 2658723589 1345784568 9034579134 6912367921 7262359572 4780958512 3482356780 1234578901 6703601412 3595736251 ]

#13a CCTCTGATGT CGAGACTTAT TACAACGGGA ATGGTGAAGT GTCTTGGTAT GAAGCAAATA GAGTTCCATG CACCTGCTCT GGATTACAGC AATATGCTAT CATTCCTGGG GTAATAATTT CCCGTTACGA

#13b TTCTGATCAC ACGAGTCCGC CGTGGTAAAG GCTACATTTC AGGACAACGC TTCAATTGCT TGAAATTGCA TTTTCAAATC AAGGCTTGAA TTGGGATGGA ATCCTTCTTT TGTGAGTGAG TTTACATTTG

[ 2222222222 2222222222 2222222222 2222222222 2222222222 2222222222 2222222222 3333333333 3333333333 3333333333 3333333333 3333333333 4444444444 ]

[ 2222222223 3333333333 3344444444 4455555555 5666666677 7778888889 9999999999 0000000000 1111111111 1111111111 1111111224 5556777789 1133334555 ]

[ 0112234590 0255666678 8904447788 9933455669 9011268912 5780233450 0225677799 1233578999 0000111122 2233334444 4445566114 0082344842 5723573078 ]

[ 2591763495 8239256724 5544670925 2716107130 4614623592 8366069511 2690326826 6302000147 0135058915 6715790123 4573445097 4513014084 8354119687 ]

#13a TCTGAGTGCC ATCCTTAGGG TTAGAACGAT CTGTTATAAT CGGTATCAAG TGGTGCCCTT TTCGTGGTTG GACATAATTT ATAGCCATGT GCATTAAGGC TGATAAGACA GGACCCTATC GTGGGTATAA

#13b CTCAGACATA CGATGGGAAA CCGATCTACC TCACCGCCGG AATCGATGCA CTACATTTCC AATACAACCA AGAGCTGCAA TATCGTGATA ATCAATGTTT CAGGTCAGTG AACTTTCGCA ACAATCGCCG

[ 1111 1111111111 1111111111 1111111111 1111111111 1111111111 1111111111 1111111111 1111111111 1111]

[ 4444555566 6666777788 8889999999 9999991111 1111111111 1111111112 2222222222 2222222222 2222222222 2222222222 2222222222 2222233333 3333]

[ 5789026912 2229356711 3770222222 2222230000 0000011111 1233333351 1111222222 2222233344 4444444455 5555555666 6666777777 7778801234 7778]

[ 9780325353 3678954045 4075133445 5567803455 6699900011 7922334503 6888134555 7777900613 5577889901 3445688024 5557033344 4561226728 5880]

[ 3188902212 8243458250 1268425270 3986911069 2437935858 8845050042 9013689278 1249168445 0918013411 7036425050 2370235814 7639151397 8156]

#13a CGACCCATAG TAGGGCTTGC TATGGCCATC CCCCGAACAT CTCGCTGCGA CTAATTTCTC CCGCTAATTT GTGTCCGTGT TATGCCGTGT TTCCTTTCCG GTAGAGAGCC GCCGCGTTCT TTCT

#13b TAGATTGCGA CGTCCTCAAA CGAATTTCCT TTTTATGAGG TCTAACAAAC TCGTCCCTCT ATATCTGAGC TGAGTTACTC CTCATTACTC CCTACGATTA TCCATTCAAA AGTATAACAC CCTC

**Nucleotides with coverage <100%**

[ 3338999999 9999999999 9999999999 9999999999 9999999999 9999999999 9999999999 9999999999 9999999999 9999999999 9999999999 9999999999 9999999999 ]

[ 0006333333 3333333333 3333333333 3333333333 3333333333 3333333333 3333333333 3333333333 3333333333 3334444444 4444444444 4444444444 4444444444 ]

[ 4484111111 1112222222 2223333333 3334444444 4445555555 5556666666 6667777777 7778888888 8889999999 9990000000 0001111111 1112222222 2223333333 ]

[ 2312123456 7890123456 7890123456 7890123456 7890123456 7890123456 7890123456 7890123456 7890123456 7890123456 7890123456 7890123456 7890123456 ]

#13a GATT------ ---------- ---------- ---------- ---------- ---------- ---------- ---------- ---------- ---------- ---------- ---------- ----------

#13b ----TATAAC CCAAATCATT CATACCTCTC TCAANNNNNN NNNNNNNNNN NNNNNNNNNN NNNNNNNNNN NNNNNNNNNN NNNNNNNNNN NNNNNNNNNN NNNNNNNNNN NNNNNNNNNN NNNNNNNNNN

[...IS...]

[ 1111111111 1111111111 1111111111 1111111111 1111111111 1111111111 1111111111 1111111111 1111111111 1111111111 1111111111 1111111111 1111111111 ]

[ 0000000000 0000000000 0000000000 0000000000 0000000000 0000000000 0000000000 0000000000 0000000000 0000000000 0000000000 0000000000 0000000000 ]

[ 8888888888 8888888888 8888888888 8889999999 9999999999 9999999999 9999999999 9999999999 9999999999 9999999999 9999999999 9999999999 9999999999 ]

[ 6667777777 7778888888 8889999999 9990000000 0001111111 1112222222 2223333333 3334444444 4445555555 5556666666 6667777777 7778888888 8889999999 ]

[ 7890123456 7890123456 7890123456 7890123456 7890123456 7890123456 7890123456 7890123456 7890123456 7890123456 7890123456 7890123456 7890123456 ]

#13a ---------- ---------- ---------- ---------- ---------- ---------- ---------- ---------- ---------- ---------- ---------- ---------- ----------

#13b NNNNNNNNNN NNNNNNNNNN NNNNNNNNNN NNNNNNNNNN NNNNNNNNNN NNNNNNNNNN NNNNNNNNNN NNNNNNNNNN NNNNNNNNNN NNNNNNNNNN NNNNNNNNNN NNNNNNNNNN NNNNNNNTTA

[ 1111111111 1111111111 1111111111 1111111111 1111111111 1111111111 1111111111 1111111111 1111111111 1111111111 1111111111 1111111111 11111111]

[ 0001111111 1111111111 1133333333 3333333333 3333333333 3333333333 3333333333 3333333333 3333333333 3333333333 3333333333 3333333333 33333333]

[ 9990000000 0000000000 0000000000 0000000000 0000000000 0000000000 0000000000 0000000000 0000000000 0000011111 1111111111 1111111111 11111111]

[ 9990000000 0001111111 1122233333 3333344444 4444455555 5555566666 6666677777 7777788888 8888899999 9999900000 0000011111 1111122222 22222333]

[ 7890123456 7890123456 7878901234 5678901234 5678901234 5678901234 5678901234 5678901234 5678901234 5678901234 5678901234 5678901234 56789012]

#13a ---------- ---------- ---------- ---------- ---------- ---------- ---------- ---------- ---------- ---------- ---------- ---------- --------

#13b GATTTGGGGT GAATAATTGG GGTAATACTC AATGAAAATC AAAGAGCAAA CTAGGAAACT AGCCGCAGGC TGTACTTGAG TACGGCAAGG CGACGTTGAC GTGGTTTGAA TTTGATTTTC GAAGAGTA

**Category 14**

**Variable nucleotides**

[ 1 1111111111 1111111111 1111111111 1111111111 1111111111 1111111111 1111111111 1111111111 1111111111 1111111111 1111111111 ]

[ 111455566 6799999990 0000000011 1111112222 2222222334 4444444444 5555555566 6666667777 7777777777 7777888888 8888888888 8888888888 8888888888 ]

[ 1556003513 5233334791 2234688934 5888890034 6678899120 0134444789 0112247802 3367790012 3444466788 8999122222 3333333444 4555555666 6677777788 ]

[ 9469291830 5101466048 4682947424 6356921473 1536812242 5481248765 2465860537 3992876708 8026909223 7369803469 0245689567 9014568024 5702347803 ]

#14a TTGTCATTTG AGGAGGGGAT ACTTGTAGGA ATGGTTGTCT TTCTGAGGCT GTGGCCGAGA TTCGCCAGGT ACCCAACAGA CATCGCGGGA TCGGTTGAAG TGCCTTGGTG TGAAGCAAAT AGAGTTCCAT

#14b C......... ...G...... .......AT. .........C .......... .......... .......... .......... .......... .......... .......... .......... ..........

#14c ...AT.CGC. G.A.CAA.GC GACCAAG..G GAAACC...C .CTCTG.AT. ...CAAC... CC..TTCA.. .GTTCGTG.. .CCTAA.AAG CT........ ....G....A .......... ..........

#14d .......... ..A.CAA.GC GACCAAG..G GAAACC...C .CTCTGAA.. ..AC.AC... ...A.T..C. GGTTCG..AG T....TAA.G C.TACGATTT CAGGACAAC. CTTCAATTGC TTGAAATTGC

#14e .......... .......... .......... .......... .......T.C ACAC.ACGAG ..TA.T..CC GGTTCG...G T....TAA.G C.TACGATTT CAGGACAAC. CTTCAATTGC TTGAAATTGC

#14f .......... .......... .......... .......... .......T.C ACAC.ACGAG ..TA.T..CC GGTTCG...G T....TAA.G C.TACGATTT CAGGACAAC. CTTCAATTGC TTGAAATTGC

#14g .CA..G..CA .A...A.AGC GACCAAG..G G..ACCACTC A..CTG.T.C ..AC.AC... ...A.T..C. GGTTCG...G T....TAA.G C.TACGATTT CAGGACAAC. CTTCAATTGC TTGAAATTGC

#14h .CA..G..CA .A...A.AGC GACCAAG..G G..ACCACTC A..CTG.T.C ..AC.AC... ...A.T..C. GGTTCG...G T....TAA.G C.TACGATTT CAGGACAAC. CTTCAATTGC TTGAAATTGC

[ 1111111111 1111111222 2222222222 2222222222 2222222222 2222222222 2222222222 2222222222 2222222222 2222222222 2222222222 2222222222 2222222222 ]

[ 8899999999 9999999000 0000000000 0000000000 0000000000 0111111111 1111111111 2222222222 2222222223 3333333333 3333333444 4444444444 4445555555 ]

[ 9900111235 5578889000 3444445555 5566666666 7777788899 9000011233 4556677889 0011112222 2344455790 0025666666 7778889003 4445566788 8991333445 ]

[ 2837346810 6835891069 6234593467 8912345689 0127814712 5234601628 4272701360 2302360258 9723459270 6934013678 0355686152 5783958103 6383279231 ]

#14a GCACCTAAGC TCTGGATTAC AGCAATATGC TATCATTTCT GGGGTAATAA TTTCCCTGCT CAACACCCGC ATTGCCCACG GGTTTAAACT ACTACTTGAG TGAGCCTAGG GACCTTTCGT CCCTGTGCCA

#14b .......... .......... .......... .......... .......... .......... .......... .......... .......... .......... .......... .......... ..........

#14c ......GGA. .......... .......... .......C.. .......... ......C.T. TG.ACGT..A ..GCT.TGGA C.AC.GGCGC CA.C.C.T.. C.C.TT..AA ..ATCCC..A T.T...ATG.

#14d ATTTTC.GAA ATCAAGGCTT GAATTGGGAT GGAATCC.TC TTTTGTGAGT GAGTTTCATC AGTACGTTTA GCGCTT..G. CAACC.GCG. ..G.TCG.GA CACA.TCC.A AT.TCCCTAC .T..ACA.GG

#14e ATTTTC.GAA ATCAAGGCTT GAATTGGGAT GGAATCC.TC TTTTGTGAGT GAGTTTCATC AGTACGTTTA GCGCTT..G. CAACC.GCG. ..G.TCG.GA CACA.TC..A AT.TCCCTAC .T..ACA.GG

#14f ATTTTC.GAA ATCAAGGCTT GAATTGGGAT GGAATCC.TC TTTTGTGAGT GAGTTTCATC AGTACGTTTA GCGCTT..G. CAACC.GCG. ..G.TCG.GA CACA.TC..A AT.TCCCTAC .T..ACA.GG

#14g ATTTTC.GAA ATCAAGGCTT GAATTGGGAT GGAATCC.TC TTTTGTGAGT GAGTTTCATC AGTACGTTTA GCGCTT..G. CAACC.GCG. ..G.TCG.GA CACA.TC..A AT.TCCCTAC .T.AACA.GG

#14h ATTTTC.GAA ATCAAGGCTT GAATTGGGAT GGAATCC.TC TTTTGTGAGT GAGTTTCATC AGTACGTTTA GCGCTT..G. CAACC.GCG. ..G.TCG.GA CACA.TC..A AT.TCCCTAC .T..ACA.GG

[ 2222222222 2222222222 2222222222 2222222222 2222222333 3333333333 3333333333 3333333333 3333333333 3333333333 3333333344 4444444444 4444444444 ]

[ 5555556666 6666666666 6667777777 7778888889 9999999000 0000011111 1111111111 1111111111 1134455567 7777778888 8888999900 0000001111 1122223333 ]

[ 5667990011 1112334456 6890227788 8880234450 4566789344 6799900001 1222222233 3333444455 5650400821 1334880012 4589278901 2355672357 7935690233 ]

[ 8244153725 6787392973 7468034903 6787170623 5139773339 2914701350 4135678912 4579024713 4438745131 3254094732 8859428314 9706498113 6064535545 ]

#14a CCGAGAAATT AAAATAGTCT ACGCTACTGA TAGCACTCCC CGCTGCTAGT AATTTATAGT AGATGAGCTA CTTAAGTATA AATGTGGACG ACAGAATGCC TGATAACTCC CCTGTAAAAT GTGTCGAGGT

#14b .......... .......... .......... .......... .......... .......... .......... .......... .......... .......... .......... ......GGC. ...C.A....

#14c TAAGTCGGG. CGT.CTAATA G.ATAGGCAG CGATG.C.T. .......... .......... .......... .......... .......... .......... .......... .......... ..........

#14d ...G..G..C CGTGCT.... .......... .......... .......... .......... .......... .......... .......... .......... .......... .......... ..........

#14e ...G..G..C CGTGCTAATA .T.TC.TCAG C....T.T.A TATCATCGAC TGCAATATCG TTTAATTTCT AAATGTCGGG TCCAGAACTA CTGCGGAATT CA...C.... TT...G..CC A......AA.

#14f ...G..G..C CGTGCTAATA .T.TC.TCAG C....T.T.A TATCATCGAC TGCAATATCG TTTAATTTCT AAATGTCGGG TCCA...... .........T .......... .......... ..........

#14g ...G..G..C CGTGCTAATA .T.TC.TCAG C....T.T.A TATCATCGAC TGCAATATCG TTTAATTTCT AAATGTCGGG TCCAA...TA CTGC.G..T. .AGGCCTATT ..ATCGGGT. AAACT.G..C

#14h ...G..G..C CGTGCTAATA .T.TC.TCAG C....T.T.A TATCATCGAC TGCAATATCG TTTAATTTCT AAATGTCGGG TCCAA...TA CTGC.G..T. .AGGCCTATT ..ATCGGGT. AAACT.G..C

[ 1111 1111111111 1111111111 1111111111 1111111111 ]

[ 4444444444 4444444444 4444444444 4555555555 5555555555 5555566666 6666666666 6667777777 8899990000 0000000000 0000000111 1111111111 1111111111 ]

[ 3334444444 5555555677 7778888999 9000000011 1223335888 8899901124 4677788888 8991111378 1611670111 1113667888 9999999000 0000000000 0011111122 ]

[ 5770146778 0167899900 2472348033 4233468803 9020397378 8922310230 0877745667 8083333904 5323719222 3340457288 2567788011 2344556677 8900356611 ]

[ 1147367691 6898703645 3918198836 8349525611 7603485671 6639299182 9305686342 9835678428 1752995127 2771708114 9467806409 0569458916 8435651614 ]

#14a GTTCTTTGGC TATAAGCACC AGGACCGCGT TAACCGAAGT ATCAGGCGGG TATGTTAATG TCGCAGCGTC GACTCTGCAC TGCGGTCGTC CCTCCTCAGG TGATATTCTC GTGAGCTGAG TTTAGGCGTA

#14b .CAA.A.AT. ..CCGTTT.T ..AGT.A.AG ....T..GAG GC...AT... .......... .......... .......... .......... .......... .......... .......... ..........

#14c .......... .......... .......... .......... .......... .......... .......T.. .......... .......... .......... .......... .......... ..........

#14d .......... .......... .A........ .......... .......... .........A .......... .......... .....GTAAT TTCTACTGAA C.CCGC.... .AA....... CC.GT..T.G

#14e TC.ACACAT. C.CCGTTG.. ..AGT..AAG ...TT.GGAG GCT..A.A.. GGCTCCGGC. .......... .......... .A........ .......... .......... .......... ..........

#14f .......... .....T.... ......AAAG CGC.TA.GAG GC..AAT.A. ......GGC. .......... .......... .A........ .......... .......... .......... ..........

#14g TC.ACACAT. ..C..T.G.. ..AG..AA.G CGC.TA.GAG GC.G.ATA.A .....CGG.. G.A.GAA.CA TGGGAGAGTT C.AAA.T... .......... CACCGCCTAA TAATTTGTGT C.CG.CT.G.

#14h TC.ACA.ATA .G...T..T. G..G.TAAAG CGC.TA.G.G GC.G.ATA.A .....CGG.. GA.T...... ..GGAGAGTT C.AAA.T... .......... CACCGCCTAA TAATTTGTGT C.CG.CT.G.

[ 1111111111 1111111111 1111111111 1111111111 1111111111 1111111111 1111111111 1]

[ 1111111111 1111111111 1111111111 1111111111 1111111111 1111111122 2222222222 2]

[ 2222222222 2222223333 3333333333 3444444444 4444555555 5556689900 1233555555 6]

[ 2223456777 7889990011 1233456788 9001234455 6699233334 4561157809 2856134578 0]

[ 1232768257 8360187803 9847031928 7892273904 6709002581 4306872917 6408818582 3]

#14a GCTCCTCAAT TCTACTGCAC AGCCTACGAA TCACTAGTCC TACTCTCAAA AGTATACGGG CTGCTTTCCT C

#14b .......... .......... .......... .......... .......... .......... .......... .

#14c .......... .......... ........C. .......... .......... .......... ..AT....TC T

#14d AACT...G.C CTC..A.TGT GT..CC.TCG .TGTCG..TA CGTA.GAGCC GCCGCGTTAA A...CCC... .

#14e .......... .......... .......... .......... .......... ......TTAA AC..CCC... .

#14f .......... .......... .......... .......... .......... ......TTAA AC..CCC... .

#14g ...TTAT.GC C..GTGATGT ..TTCCTTT. C...CGAGTA .G.ATGAGCC GCCGC.TTA. ..A.CCCT.. .

#14h ...TTAT.GC C..GTGATGT ..TTCCTTT. C...CG.GTA .G.ATGAGCC GCCGC.TTA. ..A.CCCT.. .

**Nucleotides with coverage <100%**

[ 133579999 9999999999 ]

[ 5100601555 5555555555 ]

[ 1088993111 1111122222 ]

[ 1401004234 5678901234 ]

#14a -CTTAA---- ----------

#14b -...-.---- ----------

#14c A.....---- ----------

#14d --....-TCT ATTTCTATTT

#14e -.--..---- ----------

#14f -.--..---- ----------

#14g -.--.-T--- ----------

#14h -.--.-T--- ----------

**Category 15**

**Variable nucleotides**

[ 1111111111 1111111111 1111111111 1111111111 1111111111 1111111222 2222222222 2222222222 2222222222 2222222222 2222222222 2222222222 ]

[ 115566799 0001111222 2222344444 4444455566 6666667777 7777777777 8888999011 1111111222 2222222222 2333333333 4444444445 5555555566 6666666666 ]

[ 1383515737 8892458003 6889100134 4478912402 3367780011 1123467889 2456123413 4566779111 2222244557 9005667788 3455668893 4456679900 1111334456 ]

[ 9791835010 4746469147 1681225481 4876565637 3992884601 8914502373 0742681112 4227010023 0258923592 7694160568 2839583689 2382441537 2678392973 ]

#15a CGTCGCGAAA AGGCGGCACT ACTGTCACAC ACGAGTATCC GGTTCATTTT TAAAGTAGCT GAGGGGAACT TGACGTAGCT TGGACACGGC GCACCTCCTT AATCCCATTA TGTAAGTCGG GCGTCTAATA

#15b TACTTTAGGG TAATAATGTC TTGAGTGTGG CGAGACGCGT ACCCAGACGC ATGGACGATC TGAAAAGGTC CACACCCTGC CACGGTTAAA CACATGTACC GCCTTTTCCG CCCCGAGAAA TAAATAGTCT

[ 1111111111 1111111111 1111111111 ]

[ 2222222222 2222222222 2222222223 3333333333 3333333333 3333555555 5555555555 5555555555 5666666666 6666677777 0000000011 1111111222 2222222222 ]

[ 6677777777 7788888999 9999999990 0000011111 1111111111 1112011234 4444444556 6777777888 9001112222 3477855788 4455666647 7788889466 6666777777 ]

[ 6902257788 8802457002 3466677891 2346711112 2223333444 5562615932 2344559268 9001258689 0082570122 4147449213 9912022377 9900127804 5558112223 ]

[ 7680394903 6771023237 0534939777 4143015690 4890235246 2463276020 2914038271 7260398759 6232568514 4431050880 0346606020 6727226741 2358071490 ]

#15a GATAGTGCAG CGTGCTATTT CTTTCGTTGG ACATACTCAT TCTTATAGCG GTGACAACTA CCTGCTGTAC TAATTAGGAA GGTCTCTCCT ATCATAACGG GAGGTATGTT AATTTCTTCC CGCTAAATTT

#15b AGCTACCTGA TACATCCCCA TCCCTACCAA GAGATTATGA AAGCTACTTA TAAGTGTACG ATCATGACGA CTGCCTAAGT CAATATCTGA GCAGGGGTAA ATAACTCACC GTCCCTCCTA TATCTGGAGC

[ 1111111111 1111111111 1111111111 1111111111 1111111111 111]

[ 2222222222 2222222222 3333333333 3333333333 3333333333 444]

[ 7777777889 9999999999 0000000001 1111122222 2222266677 122]

[ 4445678380 2345556678 0112355791 2224700111 1239946747 902]

[ 3461380667 2130235633 9258647272 4592457036 9851374652 363]

#15a GTGTCCGTGT TATGCCGTGT TTCCTTTCCG GTAGAGAGCC GCCGCTTATA CCC

#15b TGAGTTACTC CTCATTACTC CCTACGATTA TCCATTCAAA AGTATCGGCG TTT

**Nucleotides with coverage <100%**

[ 13333333 3333333333 3333333333 3333333333 3333333333 3333333333 3333333333 3333333333 3333333333 3333333333 3333333333 3333333333 3333333333 ]

[ 4560022222 2222222222 2222222222 2222233333 3333333333 3333333333 3333333333 3333333333 3333333333 3333333333 3333333333 3333333333 3333333333 ]

[ 1194877777 7777788888 8888899999 9999900000 0000011111 1111122222 2222233333 3333344444 4444455555 5555566666 6666677777 7777788888 8888899999 ]

[ 2153101234 5678901234 5678901234 5678901234 5678901234 5678901234 5678901234 5678901234 5678901234 5678901234 5678901234 5678901234 5678901234 ]

#15a -A-A-TAAAT CCCCAATTAT TCACCCCAAA TCTAANNNNN NNNNNNNNNN NNNNNNNNNN NNNNNNNNNN NNNNNNNNNN NNNNNNNNNN NNNNNNNNNN NNNNNNNNNN NNNNNNNNNN NNNNNNNNNN

#15b T-G-T----- ---------- ---------- ---------- ---------- ---------- ---------- ---------- ---------- ---------- ---------- ---------- ----------

[...IS...]

[ 4444444444 4444444444 4444444444 4444444444 4444444444 4444444444 4444444444 4444444444 4444444444 4444444444 4444444444 4444444444 4444444444 ]

[ 8888888888 8888888888 8888888888 8888888888 8888888888 8888888888 8888888888 8888899999 9999999999 9999999999 9999999999 9999999999 9999999999 ]

[ 2222233333 3333344444 4444455555 5555566666 6666677777 7777788888 8888899999 9999900000 0000011111 1111122222 2222233333 3333344444 4444455555 ]

[ 5678901234 5678901234 5678901234 5678901234 5678901234 5678901234 5678901234 5678901234 5678901234 5678901234 5678901234 5678901234 5678901234 ]

#15a NNNNNNNNNN NNNNNNNNNN NNNNNNNNNN NNNNNNNNNN NNNNNNNNNN NNNNNNNNNN NNNNNNNNNN NNNNNNNNNN NNNNNNNNNN NNNNNNNNNN NNNNNNNNNN NNNNNNNNNN NNNNNNNNTT

#15b ---------- ---------- ---------- ---------- ---------- ---------- ---------- ---------- ---------- ---------- ---------- ---------- ----------

[ 4444444444 4444444444 44478]

[ 9999999999 9999999999 99937]

[ 5555566666 6666677777 77799]

[ 5678901234 5678901234 56791]

#15a GAGAGAGGTA TGAATGATTT GGGA-

#15b ---------- ---------- ----A

**Category 16**

**Variable nucleotides**

[ 11111 1111111111 1111111111 1111111111 1111111111 1111111111 1111111111 1111111111 1111111111 1111112222 ]

[ 11222667 7799900000 0001111111 3344444466 7777777777 7777788888 8888888888 8888888888 8888888888 8899999999 9999990000 ]

[ 1111 1111222223 7858129122 3714912234 6884588889 2200344408 0122233467 8889912222 2333333444 4455555566 6667777778 9900111455 7888990034 ]

[ 4567890134 5678024891 3244668290 7945373571 8363524581 2314703787 5004737481 1265872358 9134578456 8903457913 4691236792 1726235957 2478095851 ]

#16a GTTTGCTCTT GATTTATTAT CGTTTCATGG ACTGATACTT GTAAATGGTT ACCAGTCGTA TTACAACGGG AATGGTGAAG TGTCTTGGTA TGAAGCAAAT AGAGTTCCAT GCACCTGCTC TGGATTACAG

#16b CAAGATAAGG TTGGCGAACC TACCCTGCAA GTAAGCGACC AAGGGAAACC CTTGACACCG CCGTGGTAAA GGCTACATTT CAGGACAACG CTTCAATTGC TTGAAATTGC ATTTTCAAAT CAAGGCTTGA

[ 2222222222 2222222222 2222222222 2222222222 2222222222 2222222222 2222222222 2222222222 2222222222 2222222222 2222222222 2333333333 3333333333 ]

[ 0000000000 0000000000 0000000001 1111111111 2222222222 3333333333 3334444444 4445555555 5566666667 7777888888 8999999999 9000000000 0111111111 ]

[ 4444555555 6666666667 7778889990 0000234588 0011223459 0025566667 8890444778 8993345566 9901126891 2578023345 9002267779 9123357899 9000011112 ]

[ 2348235678 0123457890 1670360141 2359573625 1259176349 5823925672 4554467092 5271610713 0461462359 2836606951 2126932682 6630200014 7013505891 ]

#16a CAATATGCTA TCATTCCTGG GGTAATAATT TCCCGTTACG ATCTGAGTGC CATCCTTAGG GTTAGAACGA TCTGTTATAA TCGGTATCAA GTGGTGCCCT CTTTCTGGTT GGACATAATT TATAGCCATG

#16b ATTGGGATGG AATCCTTCTT TTGTGAGTGA GTTTACATTT GCTCAGACAT ACGATGGGAA ACCGATCTAC CTCACCGCCG GAATCGATGC ACTACATTTC TCCATCAACC AAGAGCTGCA ATATCGTGAT

[ 3333333333 3333333333 3333344444 4444444444 4]

[ 1111111111 1111111124 8889900001 1333345555 6]

[ 2223333444 4444556614 0028901235 7235730789 5]

[ 5671579012 3457344597 4728314978 3541196873 7]

#16a TGCATTAAGG CTGATAAGCA AATCTCCTTG TGGGTATAAC A

#16b AATCAATGTT TCAGGTCATG TGCTATTCCA CAATCGCCGT G

**Nucleotides with coverage <100%**

[ 333678]

[ 4000904]

[ 1448446]

[ 2231599]

#16a TGATTAA

#16b -------

**Category 17**

**Variable nucleotides**

[ 1 1111111111 1111111111 1111111111 1111111111 1111111111 1111111111 1111111111 1111111111 1111111111 1111111111 1111111111 ]

[ 111223555 5666799990 0000001111 2222222233 3334444445 5555566666 6667777777 7777777777 7788888888 8888888888 8888888888 8888888888 8899999999 ]

[ 1558344356 7135455561 3445680067 0000168900 1132256662 3446902255 8990122334 4455666668 8900011134 4444555555 5666677777 7888889999 9900112233 ]

[ 9469772183 1305104664 8468294746 3569235368 1222581482 4586053939 9288767011 5848025690 9223736980 3469024568 9567901456 8024570234 7803283734 ]

#17a CTGTCGTTTG TCAAGGGGGA TACTTGTAAA TGGTTCCTCT GGACAGTCGT TACTAGCTGG TTCAGTAGTA CAACATGCGT GGAATCGGTG GAAGTGTCTT GGTATGAAGC AAATAGAGTT CCATGCACCT

#17b TCACG.C..A C.G....... .......... .....TTCTG A......... .......... .......... .......... .......... .......... .......... .......... ..........

#17c T..C..C... CTG....... .......... .......... .........C CGT.CAGCA. ...G..G.CG T.G.CCATAA .A.GCT...T ......C.G. ..G...CG.. ..G....... ..........

#17d T..C.ACCG. A.GGA..... .......... .....TTCTG A.GTG.G... .G.C..GCAC CCAGAC..CG T.G...A..C ..G......T ......C... ...G...... .......... ..........

#17e T..C..C... CTG..ACAAG CGACCAAGGG AAACC..... .A.TGACAC. .......C.. ...G.C.ACG TGGT..A... AAGGC.TAC. ATTTCAGGAC AACGCTTCAA TTGCTTGAAA TTGCATTTTC

[ 1111111112 2222222222 2222222222 2222222222 2222222222 2222222222 2222222222 2222222222 2222222222 2222222222 2222222222 2222222222 2222222222 ]

[ 9999999990 0000000000 0000000000 0000000000 0111111111 1111111111 1112222222 2222222222 2222222223 3333333333 3333344444 4444444444 4555555555 ]

[ 3455577790 0012225666 6677777788 8888889999 9000111222 2334556778 8990000122 3333444445 5666777891 2224478888 8899900012 2235566688 9000115556 ]

[ 6816706835 8910696234 5934678912 3456890127 8147125234 6016284272 7013678023 0236025893 7234589127 0692340136 7803556862 5812857858 1036382792 ]

#17a GGAGGCTCTG GATTACAGCA ATATGCTATC ATTCCTGGGG TAATAATTTC CCCGTTTGAA CGTCGATAAT GCTCTGGACG GACTGAGTCG CCAGTCCCTT AGCGCGTTTT AACACGAACC CGATCTGTAT

#17b .......... .......... .......... .......... .......... .......... .......... .......... .......... .......... .......... .......... ..........

#17c A..AA.C..A ....TT.... ....A..... .......... .......... .......... ......CC.. .........A ....AG.G.C TGCA.....G ...A..A.CG GGGGT..C.. ..TC.CAC.C

#17d AAG....... .......... .......... ...T...... .......... ..T.C.CA.C ACC..GCC.. TGC.CACGG. .TT.A.A.AC TAC..A.T.G ..T.A.CC.. G..G...CTT ..TC.C..GC

#17e A....AATCA AGGCTTGAAT TGGGATGGAA TCCTTCTTTT GTGAGTGAGT TT.A.CA.T. ...TT.C.GC ...TCA.G.. A..CA..... TAC.GAT.GG GA.A.AC.C. G....ATC.. TACCTCAC.C

[ 2222222222 2222222222 2222222222 2222222222 2222222222 2222223333 3333333333 3333333333 3333333333 3333333333 3333333333 3333333333 4444444444 ]

[ 5555556666 6666666666 6666777777 7777888888 8888999999 9999990000 0000000000 0111111111 1111111111 1111111111 1111112334 4555556888 0000000000 ]

[ 6778891112 2333334455 6678012445 7899000024 5667224567 8889990011 3455678899 9011122223 3344444445 5556666666 6778894373 6008880224 1123557789 ]

[ 3182441453 7256782739 2973468039 9249036771 7062237051 3493791737 7413514601 4136923570 2701367891 3791234567 9566781150 9235691694 0536192862 ]

#17a GATAAGTCCG GGTCGTCACT AATTCATAGA TTGCAGCGTG CCCTTTTCTG TTCGGTATTG GACAATTTTA CATTTATAGG CCATGATGCT ATTAAGGCTG ATAAGGCGCA ATGGATAAAT CTCCTTTGTC

#17b .......... .......... .......... .......... .......... .......... .......... .......... .......... .......... .......... .......... ..........

#17c ...G...T.. AT.AAA.... ...ATG.TAG CCTT.A.T.A .TT.CAAT.A .C.A.CCCCA AGAG.CGGCT TG...C...A .T..TT..TC .....AT..A .A.CAATTTG GACAGGT... ..........

#17d C.CCGAG.AA AT.AAAT.TA GTC..GCTA. C.CTGATACA .T.CCCATC. CCTA.C.C.A AGA.G.A..T .GCAATATC. GTGAT.AAT. CAATGTTTCA G......... .......... ..........

#17e .GCCG.G.A. ATC....G.. ...ATG.CA. C.T....ACA TTTCCAAT.A .C.AAC..CA AGAG.C...T .GCAATATC. GTGAT.AAT. CAATGTTTCA GGTCA.T... GACAGGTTGC TATTCCATCT

[ 1111111111 1111111111 1111111111 1111111111 1111111111 1111111111 111]

[ 4444444444 4444444444 4444444444 4444555555 5555555555 5666667778 0000000000 0000000000 0000000000 0011111111 1111111112 2222233333 333]

[ 1111222233 3344444555 5566677788 9999011124 4444445899 9111242662 2255666666 6666666677 7778888899 9925566666 6666679990 2567700122 222]

[ 5578157812 7923689022 9900112955 1355602523 3344589501 4344939794 4999022225 5667788900 1490244911 1838900111 6899908990 7035734912 358]

[ 0330286757 3695898118 1309587303 0058783383 5614067888 5103613014 9627514686 7686826013 5403945515 6185979259 6403907033 2280708818 583]

#17a AACGTGTCAA GAATATATCT CGCGTTTAGT ACAGTGAGCT AAATTGTGTA CATATTTCAC AATAGTTATG AATATATTCG TTGATATCAG CTCATGCCTT TCTTCGGACT GGTTAGCCCC TGT

#17b .......... .......... .......... .......... .......... .......... .......... .......... .......... .......... .......... .....A.TTT CT.

#17c .......... .......... .......... .......... .......... .......T.. .......... .......... .......... .......... .......... .......... ...

#17d .......... ..C.T.GG.. T.AACACGAC GAGTCAGTT. .......... .......... .......... .......... .......... .......... .......... .......... C.C

#17e GGTAAACTGG TT.C.C..TC TAA..AC... .........G GGGCCACAGG TGCGGGC.GG TGCGAGACAA CCAGACCCTA CCAGCGATGA TCTGCATACC CTACATAGTC TAACGATTTT CT.

**Nucleotides with coverage <100%**

[ 3333356 6666777888 8888888888 8888888888 8888888888 8888888888 8888888888 8888888888 8888888888 8888888888 8888888888 ]

[ 4456666666 6666666666 6660001173 3339016555 5555555555 5555555555 5555555555 5555555555 5555555555 5555555555 5566666666 6666666666 6666666666 ]

[ 1117777777 8888888888 9996680017 7786509333 3344444444 4455555555 5566666666 6677777777 7788888888 8899999999 9900000000 0011111111 1122222222 ]

[ 2313456789 0123456789 0123482327 8907547567 8901234567 8901234567 8901234567 8901234567 8901234567 8901234567 8901234567 8901234567 8901234567 ]

#17a TT-------- ---------- ---GAG-T-G CTTTAAT--- ---------- ---------- ---------- ---------- ---------- ---------- ---------- ---------- ----------

#17b -.-------- ---------- ---...-.-. .......--- ---------- ---------- ---------- ---------- ---------- ---------- ---------- ---------- ----------

#17c ..-------- ---------- ------T.-. .......TTT AACCCCAATT ATTCACCCCT AATCTAANNN NNNNNNNNNN NNNNNNNNNN NNNNNNNNNN NNNNNNNNNN NNNNNNNNNN NNNNNNNNNN

#17d --AATCCAAA CACATCCAAC TAA--.---. ...------- ---------- ---------- ---------- ---------- ---------- ---------- ---------- ---------- ----------

#17e ..-------- ---------- -----.--A- ---....--- ---------- ---------- ---------- ---------- ---------- ---------- ---------- ---------- ----------

[...IS...]

[ 1111111111 1111111111 1111111111 1111111111 1111111111 1111111111 1111111111 1111111111 1111111111 1111111111 1111111111 1111111111 1111111111 ]

[ 0000000000 0000000000 0000000000 0000000000 0000000000 0000000000 0000000000 0000000022 2222222222 2222222222 2222222222 2222222222 2222222222 ]

[ 1111111111 1122222222 2222222222 2222222222 2222222222 2222266667 7777777777 7777777755 5555555555 5555555555 5555555555 5555555555 5555555555 ]

[ 8899999999 9900000000 0011111111 1122222222 2233333333 3344433475 5555555556 6666666600 0000111111 1111222222 2222333333 3333444444 4444555555 ]

[ 8901234567 8901234567 8901234567 8901234567 8901234567 8901289040 1234567890 1234567845 6789012345 6789012345 6789012345 6789012345 6789012345 ]

#17a ---------- ---------- ---------- ---------- ---------- ---------- ---------- ---------- ---------- ---------- ---------- ---------- ----------

#17b ---------- ---------- ---------- ---------- ---------- ---------- ---------- ---------- ---------- ---------- ---------- ---------- ----------

#17c NNNNNNNNNN NNNNNNNNNN NNNNNNNNNN TTGAGAGAGG TATGAATGAT TTGGG----- ---------- ---------- ---------- ---------- ---------- ---------- ----------

#17d ---------- ---------- ---------- ---------- ---------- ---------- ---------- ---------- ---------- ---------- ---------- ---------- ----------

#17e ---------- ---------- ---------- ---------- ---------- -----AGGCA GTTTTTAAAT TTTTCCGTTA ATACTCAATG AAAATCAAAG AGCAAACTAG GAAACTAGCT GCAGGCTGTA

[ 1111111111 1111111111 1111111111 1111111111 1111111111 1111]

[ 2222222222 2222222222 2222222222 2222222222 2222222222 2222]

[ 5555555555 5555555555 5555555555 5555555555 5555666666 6666]

[ 5555666666 6666777777 7777888888 8888999999 9999000000 0000]

[ 6789012345 6789012345 6789012345 6789012345 6789012345 6789]

#17a ---------- ---------- ---------- ---------- ---------- ----

#17b ---------- ---------- ---------- ---------- ---------- ----

#17c ---------- ---------- ---------- ---------- ---------- ----

#17d ---------- ---------- ---------- ---------- ---------- ----

#17e CTTGAGTACG GCAAGGCGAC GTTGACGTGG TTTGAATTTG ATTTTCGAAG AGTA

**Category 19**

**Variable nucleotides**

[ 1111 1111111111 1111111111 1111111111 1111111111 1111111111 1111111111 1111111111 1111111111 ]

[ 1111222233 3333344444 4444555566 6677778889 9999990000 0000000000 0111111111 2222222233 3333444444 5555555555 5566666666 6777777777 7777777777 ]

[ 3559446914 6679900011 2222135713 5624780261 3334791223 4445567778 8014588899 4677889912 4469034444 0112233457 7802347778 9001222334 4445677888 ]

[ 7468788834 4502628929 0125029241 6720118426 1577159579 2365840375 8515746703 4614792335 1917392359 3576935730 1642400399 8782269591 3670103348 ]

#19a GTGGGCGAGT ACGTTCATTT TTACATTCTG ACGCGCGTCT GGGGGATACT CTCGACGCCT ACAAATGGTT TTCCTGAGGC ATGCTGGCCG TTCGCCCCTC AGGTACCCAG ACACGTAGCA TACGCGGGAT

#19b .......... .........- .....CGAC. G.A....... ....A..... .......... .......... .......... .......... .......... .......... .......... ..........

#19c .......... .......... .......... .T........ ACAA.GCGAC .C....A..A G..GGAAACC CC.TCTGAA. .....AC.AC ...A...T.. ..C.GGTTC. G.....G.T. ....TAA.GC

#19d ....A..... .......... .....C.... G.A....... .......... .......... ..G....... .......... .......... .........T .......... .......... ..........

#19e ....A..... .......... .....C.... G.A....GT. ..A.A.CGAC TGTATTATTA G..GT....G CCTTCTG.T. GCATC.CAAC CC..TT.T.. CA...GTTC. GTG......C C.TAA.AAGC

#19f ...A...... .....T.... .....CGAC. G...A..... .....GCGAC .C....A..A G..GT....G CC.TCTG.T. GCATC.CAAC CC..T..T.. CA...GTTC. GTG......C C.TAA.AAGC

#19g ...A...... .....T.... .....CGAC. G...A..... .....GCGAC .C....A..A G..GT....G CC.TCTG.T. GCATC.CAAC CC..T..T.. CA...GTTC. GTG......C C.TAA.AAGC

#19h ....A..... .......... .....CGAC. G.A....... .......... .......... .......... .......... .......... .......... .......... .......... ..........

#19i ....A..... .......... .....CGAC. G.A....... .......... .......... .......... CC.TCTG.AT .....AC.AC ...A...T.. ..C.GGTTC. G.....G.T. ....TAA.GC

#19j ....A..... .......... .....CGAC. G.A....... .......... .......... .......... .......... .......... .......... .......... .......... ..........

#19k .CA....... .......... ......G.CA ..AG..A... .....GCGAC .C....A..A G..GG..ACC CC.TCTG.A. ....CAC.AC ..TAT.TTC. ...A..TTCA GT.TAC.A.. .G..T..A..

#19l A......... .......... ....G.G.CA ..A..T...A ...A.GCGAC .C....A..A G..GGAAACC CC.TCTG.AT .....AC.AC ...A...T.. ..C.GGTTC. G.....G.T. ....TAA.GC

#19m .CA..GACAC GAACC.TCCC GCCT.CG.C. G......... .......... .......... .A........ .......... .......... .......... ......TTC. .......... ..........

[ 1111111111 1111111111 1111111111 1111111111 1111111111 1111111111 2222222222 2222222222 2222222222 2222222222 2222222222 2222222222 2222222222 ]

[ 7788888888 8888888888 8888888888 8888888888 8899999999 9999999999 0000000000 0000000000 0000000000 0001111111 1111111111 1111222222 2222222222 ]

[ 9901222233 3333344445 5555556666 6777777889 9900111233 3555678899 0013444455 5555666666 6777777888 9990000112 3345556677 8889001111 2222333444 ]

[ 4709145701 3567906780 1256791356 8134589143 6948457927 8179746902 1707345604 5789023456 9012389258 2363457127 3953683812 4781341347 1369048345 ]

#19a CGGTGGAAGT GCCTTGGTGT GAAGCAAATA GAGTTCCATG ACACCTAAGG GCTCGTGGAT TACAGCAATA TGCTATCATT CTGGGGTAAT AATTTCCCTG CTCAAACACC CGACATTGCC CACGGGGTTT

#19b ....T..... .......... .......... .......... C......... .......... .......... .......... .......... .......... .......... .......... ..........

#19c .TAC.ATTTC AGGACAAC.C TTCAATTGCT TGAAATTGCA .TTTTC.GA. .AAT.CAAGG CTTGAATTGG GATGGAATCC TCTTTTGTGA GTGAGTTTCA TCAG.TACGT TT.AGCGCTT ..G.C.AACC

#19d ....T..... .......... .......... .......... .......... .......... .......... .......... .......... .......... .......... .......... ..........

#19e T...T..... ...G...GA. .CG....G.. .......... .......GAA A.C...A... .TT....... .A........ T.T....... .......... TC..G...AT ......GCT. TGGACA.AC.

#19f T...T..... ...G...GA. .CG....G.. ..AC.T.... .......GAA A.C...A... .TT....... .A........ T.T....... .......... TC..G...AT ......GCT. TGGACA.AC.

#19g T...T..... ...G...GA. .CG....G.. ..AC.T.... .......GAA A.C...A... .TT....... .A........ T.T....... .......... TC..G...AT ......GCT. TGGACA.AC.

#19h ....T..... .......... .......... .......... .......... .......... .......... .......... .......... .......... .......... ..G....... ..........

#19i .TAC.ATTTC AGGACAAC.C TTCAATTGCT TGAAATTGCA .TTTTC.GA. .AAT.CAAGG CTTGAATTGG GATGGAATCC TCTTTTGTGA GTGAGTTTCA TC........ .......... ..........

#19j ....T..... .......... .......... .......... .......... .......... .......... .......... .......... .......... .......... ..G....... ..........

#19k .......... .T......A. .......... .......... ......GGA. ....C..... .......... .......... .......... ........C. T.TG..ACGT ...A..GCT. TGGAC..AC.

#19l .TAC.ATTTC AGGACAAC.C TTCAATTGCT TGAAATTGCA .TTTTC.GA. .AAT.CAAGG CTTGAATTGG GATGGAATCC TCTTTTGTGA GTGAGTTTCA TCAG.TACGT TT.AGCGCTT ..G.C.AACC

#19m ....T..... .......... .......... .......... .......... .......... .......... .......... .......... .......... .......... .......... ..........

[ 2222222222 2222222222 2222222222 2222222222 2222222222 2222222222 2222222222 2222222222 2222222222 2222222222 2222222222 2223333333 3333333333 ]

[ 2222223333 3333333333 3333344444 4444444444 4445555555 5555556666 6666666666 6666677777 7777777778 8888888999 9999999999 9990000000 0000000000 ]

[ 5566790012 2566666677 7888900133 4445666788 8993344455 6679990011 1112234455 6689901224 6678888880 1234457002 3456677788 8990012233 3445666777 ]

[ 6902381703 4512478914 6679739239 6894069214 7493803429 3552564836 7893840308 4854796140 0350146788 7281731348 1624504802 8483685724 5342579023 ]

#19a AAATACTACG TACTTGAGTG AGCCTTACGC GACCTTTCGT CCCGTGCCAC CGAGCAAATT AAACATAGTC TACTGCATAA CTCTGACTAC AACTCCCCCA TCGCCTAGCA CTAACAGGAA CTGTATGTTC

#19b .......... .......... .......... .......... .......... .......... .......... .......... .......... .......... .......... .......... ..........

#19c ..G.CG.... G.TCG.GACA CA.TC...A. AT.TCCCTAC .T.ACA.GG. ..G...G..C CGT.GCTAAT A.T..T.C.. ..TCAG.C.. ..T.T...A. .TAT.C.A.. TC.......G ..ACT.....

#19d .......... .......... .......... ...TC..... .......... .......... .......... .......... .......... .......... .......... .......... ..........

#19e .GGGCG.... GC.C....CA C.ATCGGG.T ...TCCC... ...ACA.G.T GAGT.CGGG. CGT..CTAAT ...A.AG..G .CT.A..CTT T...TTA.A. .TAT.C...C .CGCT..A.. TA..GGTCCT

#19f .GGGC..G.A .C.C....CA C.ATCGGG.T ...TCCC... ...ACA.G.T GAGTTCG... .....C..CT ...A.A...G .CT.A..CTT ....TTA.A. .TAT.C...C .CGCT..A.. TA..GGTCCT

#19g .GGGC..G.A .C.C....CA C.ATCGGG.T ...TCCC... ...ACA.G.T GAGTTCG... .....C..CT .......... ......G... .......... .......... .......... ..........

#19h .......... .......... .......... ...TC..... .......... .......... ...T...... .......... .......... .......... .......... .......... ..........

#19i .......... .......... .......... ...TC..... .......... .......... ...T...... .......... .......... .......... .......... .......... ..........

#19j .......... .......... .......... ...TC..... .......... .......... ...T...... .......... .......... .......... .......... .......... ..........

#19k G.G.CGCCA. .C.C.T..C. C.TT....A. ..ATCCC..A T.T..ATG.T AAG..CGGG. CGT..CTAAT AG..AT.AG. T.GCAG.CGT .G.C.T.TTT CT.TTCG.T. T.G..GA.C. ....T...A.

#19l ..G.CG.... G.TCG.GACA CA.TC...A. AT.TCCCTAC .T.ACA.GG. ..G...G..C CGT.GCTAAT A.T..T.C.. ..TCAG.C.. ..T.T..... .T.T.C.A.. TC.......G ..ACT.....

#19m .......... .......... .......... ...TC..... .......... .......... .......... .......... .......... .......... .......... .......... ..........

[ 3333333333 3333333333 3333333333 3333333333 3333333333 5555555555 5555555555 5555555555 5555555555 5555555555 5555566666 6666666666 6666666666 ]

[ 0000000011 1111111111 1111111111 1111111111 1111111334 1112222222 2333444444 4455555556 6667777777 7788888899 9999900000 0111111112 2333344445 ]

[ 7777889900 0001111222 2222333333 3444444445 5556678151 5991177779 9347223445 6901123693 4890124466 7934668800 6679901468 8125578992 8046903783 ]

[ 5789124703 4681378124 6789012457 8023456780 4677897261 8125645680 2439247365 3105843965 0394250817 5092294718 1564646622 5840787029 0163274529 ]

#19a CGTCTATTTA TAGGTATGAG AATGAGCTAC TTAATGTTAA TAAAAGATTG TTGGGGAGTA ACTTGAGCAG CAATGCCGAA CACTCCCCTG TAAAAATGTC CTCCTGATGT TCTGTTGGCA TTGGTAACGA

#19b .......... .....G.... .......... .......... ......G... .......... .......... .......... .......... .......... ...TG..... .......... ..........

#19c .....GCAAT ACC.GT...T T.AATTTCTA AATG.T.C.G GGTC..GGCA G..AA..... CT..AC.TGC .GGAATTA.. .C...T..A. .GGGCG.AAG TC.......C .AC....T.. C..A.GG..G

#19d .......... .......... .......... .......... ......G... .......... .......... .......... .......... .......... ...TG..... .......... .......T.G

#19e GTCTGT...C ..AACT.ATT TT..TT.ATA ....ATC... G..C.AGG.. GAC..AG.GT ....ACATGC ..G..T.A.C .C.ATT.... ..GGT..AA. .CT...G... .AC.ACAT.. .GA..G..AG

#19f GTCTGT...C ...ACT.ATT TT..TT.ATA ....ATC... G..C.AG... GAC..AGAGT ....ACATGC ..G..T.A.. TC.ATT.... ..GGC..... .C...A..TC AA..A..T.. C...CG..AG

#19g .......... .......... .......... .......... ......G... .......... .......... T......... .......... .......... .......... .......... ..........

#19h .....GCAAT ATC.GT...T T.AATTTCTA AATG.T.C.G GGTC..GGCA G..AA..... CTCCAC.TGC .GG..T.A.. .C....TT.. .G..C.CA.. .......... ....A.ATAG ......G..G

#19i .....GCAAT ATC.GT...T T.AATTTCTA AATG.T.C.G GGTC..GGCA G..AA..... CTCCAC.TGC .GG..T.A.. .C....TT.. .G..C.CA.. .......... ....A.ATAG ......G..G

#19j .....GCAAT ATC.GT...T T.AATTTCTA AATG.T.C.G GGTC..GGCA G..AA..... CTCCAC.TGC .GG..T.A.. .C....TT.. .G..C.CA.. .......... ....A.ATAG ......G..G

#19k .......... ....CTCAT. T...CTTATA ....G.C.G. GT..G.GGCA A......... .T..AC.TGC ..GA.T.AGC .CTATT..AT CGGGT..AA. .CT...GCTC .AC.ACAT.. C....G..AG

#19l .....GCAAT ATC.GT...T T.AATTTCTA AATG.T.C.G GGTC..GGCA G..AA..... CT..AC.TGC .GG....... .......... .......... .......... .......... .G........

#19m .......... .......... .......... .......... ......G... .......... .......... .......... .......... .......... ...TG..... .ACAACAT.. C....G...G

[ 111 1111111111 1111111111 1111111111 1111111111 1111111111 1111111111 ]

[ 6666666666 6666666677 7777777777 7777777788 8888888999 9999999999 9999999000 0000000000 0000001111 1111111111 1111112222 5555555555 5566666666 ]

[ 5556666777 7788889900 1111111111 1122235588 8888899000 1222234455 7778889000 0000001111 1146780233 3444445555 6666791222 1335678999 9911111122 ]

[ 4691445345 7901140147 0222334567 7845854534 5788817567 0013470779 1340238011 2245671112 2245492088 8113440359 0133015223 2149174223 5633566644 ]

[ 2099479456 3732621752 9245037405 7830698472 2958985757 7020279067 9934429134 3410630190 1223737647 9571023507 4127787030 3099584465 9114537925 ]

#19a CCAAAGCGCT AGGAGGGCAC ATAAATGTCG CTAGTCGTGT ATGTCATAAA GACGTTTTCT CACCCTACTT CGAAAGGATC TGCAGTAGGG CCTGTTACAC GTGTCCCCCA AAGGCCTTCG GAATACATGG

#19b ..G.GTT... ........G. .......... ..G.C..C.. .CTCT.CC.. .......... .C........ .......... ......G..A TTCACCT... .......... G..A....T. ..........

#19c .T..GT..AC G.AG....G. G......... ....C.A..G G..C..C.GG A......... .......... .......... ...G.....A TTCACCT.G. ......TTTC G...T...T. ..........

#19d ..G.GTTAAC G...TT.TG. .........A ..G.C.A.A. ...C.GC.GG .GTA.GG... T.TT...... ........GA GA.GAG.C.A TTCACCT... .......... G.......T. ..........

#19e T..C..T.AC G.AG..A.GT .GGGGCACT. ..GAC.A.A. ...C.GC.GG .GTA.GG... T.TT...... ........GA GA.GAG.C.A TTCACCT... .......... G.......T. ..........

#19f .TG.GT..AC G.AG....G. G......... .G..C.A..G GCTC..C.GG .......... ...T...... .......... .......CTA TTCACCT..A AATC.T.... GG......T. ..........

#19g ..G.GTTAAC GA.G.T.TG. .........A ..G.CT.CA. ...C.GC.GG .GTA.GGCAC ...T...... .......... ..T....CTA TTCACCT..A AATC.T.... GG......T. ..........

#19h .T........ ........G. .......... .........G GCTC..C.GG .GT....... .......... .......... ...G...C.A TTCACCT.G. ......TTTC G.A..TCCTA TT...T.C..

#19i .T........ ........G. .......... .........G GCTC..C.GG .GT....... .......... .......... ...G...C.A TTCACCT.G. ......TTTC G.A..TCCTA TT...T.C..

#19j .T........ ........G. .......... .........G GCTC..C.GG .GT....... .......... .......... ...G...C.A TTCACCT.G. ......TTTC G.A..TCCTA TT...T.C..

#19k ....G..... ........G. .......... T....T.CA. ...C.GC.GG .GTA.GGCAC ....ACGTAA TAGGTTTC.. .......CTA TTCACCTT.. ....AT.... G.......T. ........TA

#19l ..G.GTTAAC GA.G.T.TG. .........A ..G.CT.CA. ...C.GC.GG .GTA.GGCAC ....ACGTAA TAGGTTTC.. .......CTA TTCACCTT.. ....AT.... G.......T. ........TA

#19m T..C..TAA. G.......G. .......... ....C.A..G GCT...C.GG AG.AG..... .......... .......... .........A TTCACC..GA AA....TTTC G.......T. ..GAG.GC..

[ 1111111111 1111111111 1111111111 1111111111 1111111111 1111111111 1111111111 1111111111 1111111111 1111111111 1111111111 1111111111 1111111111 ]

[ 6666666666 6666777777 7777777777 7777777777 7777777777 7777777777 7777777777 7777777777 7778888888 8888888888 8888888888 8888888888 8888888888 ]

[ 2667777777 7789222444 5555566666 6666666666 7777777777 7888888888 8999999999 9999999999 9990000000 0000000001 1111111111 1222223333 3333344444 ]

[ 4042555667 8813188159 0116801112 3445578889 0001122349 9024455568 9001111222 2344445667 7890112344 4455678880 0236677778 9559990246 7889902223 ]

[ 7909256161 1555414251 5694912351 9584501490 3461339800 6116967872 1370238125 6323584392 5866473234 5707824591 2545703698 5132897631 0024871580 ]

#19a CCTCCGTCCC TAACCCTCAC GGGCACCGCC TACATAATTT GTGTTCTCGC TGCGAGCTTT ATAGCCCTCG TGATGTAGTT CCTCTTACCA ACGCCGGTAT GCAGAGCCGC CGCTTCAGTA AGTATGAAGG

#19b ..AT.AATTT CC.T...... ....G....T CT.CG..AGC TGAG..CT.G CT.TGAAC.C TCGA..TC.A CAG...GTCC ..C..CGTTG .T.......C .T........ .......... ..........

#19c .......... .C......G. ...TGATATT CT.CGTGAGC TGAG.T.TAG C..T....CC TC.ATT...A CTGCAC..CC TAC.GA.T.. ...T.ATCC. A.TTCAAAAG TATCCTG.C. GACGCTCT..

#19d .......... .C..T....T ....G....T CTTCG..... .........G .......... .......... .......... .......... .......... .......... .........C ......CTTA

#19e .......... .C..T....T ....G....T CTTCG..... .........G .......... .......... .......... .......... .......... .......... .........C ......CTTA

#19f .......... .C..T....T ....G....T CTTCG..... .........G .......... .......... .......... .......... .......... .......... .......... ..........

#19g .......... .C..T....T ....G....T CTTCG..... .........G .......... .......... .......... .......... .......... .......... .......... ..........

#19h .......... .C..T....T A...G....T CTTCG..AGC TGAG...T.. C..T....CC TC.ATT...A CTGCAC..CC TAC.GA.T.. ...T.ATCC. A.TTCAAAAG TAT....... ..........

#19i .......... .C..T....T A...G....T CTTCG..AGC TGAG...T.. C.TT...CCC TCGA..TCTA CAG...G.CC ..CTGC.T.. ..ATT.T..C .T........ .......T.. ..........

#19j .......... .C..T....T A...G....T CTTCG..AGC TGAG...T.. C.TT...CCC TCGA..TCTA CAG...G.CC ..CTGC.T.. ..ATT.T..C .T........ .......T.. ..........

#19k TT........ .C........ ...TGATATT CT.CGTGAGC TGAG.T.TAG C..T....CC TC.ATT...A CTGCAC..CC TAC.GA.T.. ..TT.ATCC. A.TTCAAAAG TAT....... ..........

#19l TT........ .C........ ...TGATATT CT.CGTGAGC TGAG.T.TAG C..T....CC TC.ATT...A CTGCAC..CC TAC.GA.T.. ..TT.ATCC. A.TTCAAAAG TAT....... ..........

#19m ....T..... .CG..ACTG. .AA.G....T CT.CG..AGC TGAGC.CT.. CTTT...CCC TCGA..TCTA CAG...G.CC ..CTGC.T.. C.ATT.T..C .T...A.... .......... ......CT.A

[ 1111111111 2222222222 2222222222 222222222]

[ 8888888888 0000000000 0000000000 000000000]

[ 4455566666 4444444555 5556666668 888999999]

[ 4528900023 0014679015 6792344796 799122455]

[ 5778306757 1641204459 5326836942 529926701]

#19a CACCAAGTTA ATCGCCCCAT TCAGTGCCGT TTCCTCTTA

#19b .......... ..TA...... .........C CCT......

#19c T.-TGG.... ...A...... .........C CCT.CTC..

#19d .....GT... CC.AAATTCC CACAGATT.C CCT.CTC..

#19e .....GT... CC.AAATTCC CACAGATT.C CCT.CTC..

#19f .G-TGG.CGG .......... .......... ...ACTC..

#19g .G-TGG.CGG .......... .......... ...ACTC..

#19h .......CGG .......... .......... ....CTC..

#19i .......... ...A.A.... .T.......C CC..CTC..

#19j .......... ...A.A.... .T.......C CC..CTC..

#19k .......CGG .......... .......... ....CTC..

#19l .......CGG .......... .......... ....CTC..

#19m ..A...A... ...A.A.... ........A. .......AC

**Nucleotides with coverage <100%**

[ 333333 3333333333 3333333333 3333333333 3333333333 3333333333 3333333333 3333333333 3333333333 3333333333 3333333333 3333333333 3333333333 ]

[ 4456000044 4444444444 4444444444 4444444444 4444444444 4444444444 4444445555 5555555555 5555555555 5555555555 5555555555 5555555555 5555555555 ]

[ 1119558844 4444445555 5555556666 6666667777 7777778888 8888889999 9999990000 0000001111 1111112222 2222223333 3333334444 4444445555 5555556666 ]

[ 8968018923 4567890123 4567890123 4567890123 4567890123 4567890123 4567890123 4567890123 4567890123 4567890123 4567890123 4567890123 4567890123 ]

#19a -T-C--TT-- ---------- ---------- ---------- ---------- ---------- ---------- ---------- ---------- ---------- ---------- ---------- ----------

#19b --A---..CC CCAAATCATT CATACCTCTC TCAANNNNNN NNNNNNNNNN NNNNNNNNNN NNNNNNNNNN NNNNNNNNNN NNNNNNNNNN NNNNNNNNNN NNNNNNNNNN NNNNNNNNNN NNNNNNNNNN

#19c -.-.------ ---------- ---------- ---------- ---------- ---------- ---------- ---------- ---------- ---------- ---------- ---------- ----------

#19d -.A.--..-- ---------- ---------- ---------- ---------- ---------- ---------- ---------- ---------- ---------- ---------- ---------- ----------

#19e T.A.A-.--- ---------- ---------- ---------- ---------- ---------- ---------- ---------- ---------- ---------- ---------- ---------- ----------

#19f -.A.A-.--- ---------- ---------- ---------- ---------- ---------- ---------- ---------- ---------- ---------- ---------- ---------- ----------

#19g -.A.--..-- ---------- ---------- ---------- ---------- ---------- ---------- ---------- ---------- ---------- ---------- ---------- ----------

#19h -.A.------ ---------- ---------- ---------- ---------- ---------- ---------- ---------- ---------- ---------- ---------- ---------- ----------

#19i -.A.------ ---------- ---------- ---------- ---------- ---------- ---------- ---------- ---------- ---------- ---------- ---------- ----------

#19j -.A.------ ---------- ---------- ---------- ---------- ---------- ---------- ---------- ---------- ---------- ---------- ---------- ----------

#19k -.-.AA.--- ---------- ---------- ---------- ---------- ---------- ---------- ---------- ---------- ---------- ---------- ---------- ----------

#19l -.-.------ ---------- ---------- ---------- ---------- ---------- ---------- ---------- ---------- ---------- ---------- ---------- ----------

#19m -CA.--..-- ---------- ---------- ---------- ---------- ---------- ---------- ---------- ---------- ---------- ---------- ---------- ----------

[...IS...]

[ 4444445555 5555555555 5555555555 5555555555 5555555555 5555555555 5555555555 5555555555 5555555555 5555555555 5555555555 5555555555 5555555555 ]

[ 9999990000 0000000000 0000000000 0000000000 0000000000 0000000000 0000000000 0000000000 0000000000 0000000000 0000001111 1111111111 1111111111 ]

[ 9999990000 0000001111 1111112222 2222223333 3333334444 4444445555 5555556666 6666667777 7777778888 8888889999 9999990000 0000001111 1111112222 ]

[ 4567890123 4567890123 4567890123 4567890123 4567890123 4567890123 4567890123 4567890123 4567890123 4567890123 4567890123 4567890123 4567890123 ]

#19a ---------- ---------- ---------- ---------- ---------- ---------- ---------- ---------- ---------- ---------- ---------- ---------- ----------

#19b NNNNNNNNNN NNNNNNNNNN NNNNNNNNNN NNNNNNNNNN NNNNNNNNNN NNNNNNNNNN NNNNNNNNNN NNNNNNNNNN NNNNNNNNNN NNNNNNNNNN NNNNNNNNNN NNNNNNNNNN NNNNNNNTTA

#19c ---------- ---------- ---------- ---------- ---------- ---------- ---------- ---------- ---------- ---------- ---------- ---------- ----------

#19d ---------- ---------- ---------- ---------- ---------- ---------- ---------- ---------- ---------- ---------- ---------- ---------- ----------

#19e ---------- ---------- ---------- ---------- ---------- ---------- ---------- ---------- ---------- ---------- ---------- ---------- ----------

#19f ---------- ---------- ---------- ---------- ---------- ---------- ---------- ---------- ---------- ---------- ---------- ---------- ----------

#19g ---------- ---------- ---------- ---------- ---------- ---------- ---------- ---------- ---------- ---------- ---------- ---------- ----------

#19h ---------- ---------- ---------- ---------- ---------- ---------- ---------- ---------- ---------- ---------- ---------- ---------- ----------

#19i ---------- ---------- ---------- ---------- ---------- ---------- ---------- ---------- ---------- ---------- ---------- ---------- ----------

#19j ---------- ---------- ---------- ---------- ---------- ---------- ---------- ---------- ---------- ---------- ---------- ---------- ----------

#19k ---------- ---------- ---------- ---------- ---------- ---------- ---------- ---------- ---------- ---------- ---------- ---------- ----------

#19l ---------- ---------- ---------- ---------- ---------- ---------- ---------- ---------- ---------- ---------- ---------- ---------- ----------

#19m ---------- ---------- ---------- ---------- ---------- ---------- ---------- ---------- ---------- ---------- ---------- ---------- ----------

[ 5555555555 5555555555 5555556666 6666666677 7777777777 7777777777 7777777777 7777777777 7777777777 7777777777 7777777777 7777777777 7777777777 ]

[ 1111111111 1111111111 1111117777 7777777735 5555555555 5555555555 5555555555 5555555566 6666666666 6666666666 6666666666 6666666666 6666666666 ]

[ 2222223333 3333334444 4444440001 1111111146 6666666677 7777777788 8888888899 9999999900 0000000011 1111111122 2222222233 3333333344 4444444455 ]

[ 4567890123 4567890123 4567897890 1234567871 2345678901 2345678901 2345678901 2345678901 2345678901 2345678901 2345678901 2345678901 2345678901 ]

#19a ---------- ---------- ------CATA GGCATTCT-- ---------- ---------- ---------- ---------- ---------- ---------- ---------- ---------- ----------

#19b GATTTGGGGT GAATAATTGG GGTTTA---- --------C- ---------- ---------- ---------- ---------- ---------- ---------- ---------- ---------- ----------

#19c ---------- ---------- ------.... ........-- ---------- ---------- ---------- ---------- ---------- ---------- ---------- ---------- ----------

#19d ---------- ---------- ------.... ........-- ---------- ---------- ---------- ---------- ---------- ---------- ---------- ---------- ----------

#19e ---------- ---------- ------.... ........-- ---------- ---------- ---------- ---------- ---------- ---------- ---------- ---------- ----------

#19f ---------- ---------- ------.... ........-- ---------- ---------- ---------- ---------- ---------- ---------- ---------- ---------- ----------

#19g ---------- ---------- ------.... ........-- ---------- ---------- ---------- ---------- ---------- ---------- ---------- ---------- ----------

#19h ---------- ---------- ------.... ........-- ---------- ---------- ---------- ---------- ---------- ---------- ---------- ---------- ----------

#19i ---------- ---------- ------.... ........-- ---------- ---------- ---------- ---------- ---------- ---------- ---------- ---------- ----------

#19j ---------- ---------- ------.... ........-- ---------- ---------- ---------- ---------- ---------- ---------- ---------- ---------- ----------

#19k ---------- ---------- ------.... ........-- ---------- ---------- ---------- ---------- ---------- ---------- ---------- ---------- ----------

#19l ---------- ---------- ------.... ........-- ---------- ---------- ---------- ---------- ---------- ---------- ---------- ---------- ----------

#19m ---------- ---------- ------.... ........-C CCAAATTTCA AGTTGAAGTT AGCNNNNNNN NNNNNNNNNN NNNNNNNNNN NNNNNNNNNN NNNNNNNNNN NNNNNNNNNN NNNNNNNNNN

[...IS...]

[ 8888888888 8888888888 8888888888 8888888888 8888888888 8888888888 8888888888 8888888888 8888888888 8888888888 8888888888 8889999999 9999999999 ]

[ 6666666677 7777777777 7777777777 7777777777 7777777777 7777777777 7777777777 7777777777 7777777777 7777777777 7777777788 8881111111 1111133339 ]

[ 9999999900 0000000011 1111111122 2222222233 3333333344 4444444455 5555555566 6666666677 7777777788 8888888899 9999999900 0004444445 5555522234 ]

[ 2345678901 2345678901 2345678901 2345678901 2345678901 2345678901 2345678901 2345678901 2345678901 2345678901 2345678901 2344567890 1234578906 ]

#19a ---------- ---------- ---------- ---------- ---------- ---------- ---------- ---------- ---------- ---------- ---------- ---------- -----GCTT-

#19b ---------- ---------- ---------- ---------- ---------- ---------- ---------- ---------- ---------- ---------- ---------- ---------- -----....-

#19c ---------- ---------- ---------- ---------- ---------- ---------- ---------- ---------- ---------- ---------- ---------- ---------- -----....-

#19d ---------- ---------- ---------- ---------- ---------- ---------- ---------- ---------- ---------- ---------- ---------- ---------- ----------

#19e ---------- ---------- ---------- ---------- ---------- ---------- ---------- ---------- ---------- ---------- ---------- ---------- ----------

#19f ---------- ---------- ---------- ---------- ---------- ---------- ---------- ---------- ---------- ---------- ---------- ---TAGGTGA TAACC....-

#19g ---------- ---------- ---------- ---------- ---------- ---------- ---------- ---------- ---------- ---------- ---------- ---------- ----------

#19h ---------- ---------- ---------- ---------- ---------- ---------- ---------- ---------- ---------- ---------- ---------- ---------- -----....-

#19i ---------- ---------- ---------- ---------- ---------- ---------- ---------- ---------- ---------- ---------- ---------- ---------- -----....-

#19j ---------- ---------- ---------- ---------- ---------- ---------- ---------- ---------- ---------- ---------- ---------- ---------- -----....-

#19k ---------- ---------- ---------- ---------- ---------- ---------- ---------- ---------- ---------- ---------- ---------- ---------- ----------

#19l ---------- ---------- ---------- ---------- ---------- ---------- ---------- ---------- ---------- ---------- ---------- ---------- ----------

#19m NNNNNNNNNN NNNNNNNNNN NNNNNNNNNN NNNNNNNNNN NNNNNNNNNN NNNNNNNNNN NNNNNNNNNN NNNNNGGCTA ACTTCATTAT AGAACTTTCA AAAAGGTTTC ATA------- -----....G

[ 11111 1111111111 1111111111 1111111111 1111111111 1111111111 1111111111 1111111111 1111111111 1111111111 1111111111 1111111111 ]

[ 9999999999 9999900112 2222222222 2222222222 2222222222 2222222222 2222222222 2222222222 2222222222 2222222222 2222222222 2222222222 2222222222 ]

[ 9999999999 9999900663 3333333333 3333333333 3333333333 3333333333 3333333333 3333333333 3333333333 3333333333 3333333333 3333333344 4444444444 ]

[ 4445555555 5556677120 0000000011 1111111122 2222222233 3333333344 4444444455 5555555566 6666666677 7777777788 8888888899 9999999900 0000000011 ]

[ 7890123456 7890129901 2345678901 2345678901 2345678901 2345678901 2345678901 2345678901 2345678901 2345678901 2345678901 2345678901 2345678901 ]

#19a ---------- -----AA--- ---------- ---------- ---------- ---------- ---------- ---------- ---------- ---------- ---------- ---------- ----------

#19b ---------- -----..--- ---------- ---------- ---------- ---------- ---------- ---------- ---------- ---------- ---------- ---------- ----------

#19c ---------- -----..--- ---------- ---------- ---------- ---------- ---------- ---------- ---------- ---------- ---------- ---------- ----------

#19d ---------- -----.---- ---------- ---------- ---------- ---------- ---------- ---------- ---------- ---------- ---------- ---------- ----------

#19e ---------- -----.---- ---------- ---------- ---------- ---------- ---------- ---------- ---------- ---------- ---------- ---------- ----------

#19f ---------- -----..TAG ACTTGTTCGG AATCTAGTTC ACTNNNNNNN NNNNNNNNNN NNNNNNNNNN NNNNNNNNNN NNNNNNNNNN NNNNNNNNNN NNNNNNNNNN NNNNNNNNNN NNNNNNNNNN

#19g ---------- -----..TA- ---------- ---------- ---------- ---------- ---------- ---------- ---------- ---------- ---------- ---------- ----------

#19h ---------- -----..--- ---------- ---------- ---------- ---------- ---------- ---------- ---------- ---------- ---------- ---------- ----------

#19i ---------- -----..--- ---------- ---------- ---------- ---------- ---------- ---------- ---------- ---------- ---------- ---------- ----------

#19j ---------- -----..--- ---------- ---------- ---------- ---------- ---------- ---------- ---------- ---------- ---------- ---------- ----------

#19k ---------- ------.--- ---------- ---------- ---------- ---------- ---------- ---------- ---------- ---------- ---------- ---------- ----------

#19l ---------- ------.--- ---------- ---------- ---------- ---------- ---------- ---------- ---------- ---------- ---------- ---------- ----------

#19m AGGTGGCTTG AAATA..TA- ---------- ---------- ---------- ---------- ---------- ---------- ---------- ---------- ---------- ---------- ----------

[...IS...]

[ 1111111111 1111111111 1111111111 1111111111 1111111111 1111111111 1111111111 1111111111 1111111111 1111111111 1111111111 1111111111 1111111111 ]

[ 3333333333 3333333333 3333333333 3333333333 3333333333 3333333333 3333333333 3333333333 3333333333 3333333333 3333333333 3333333333 3333333333 ]

[ 0000000000 0000000000 0000000000 0000000011 1111111111 1111111111 1111111111 1111111111 1111111111 1111111111 1111111111 1112222222 2222222222 ]

[ 6666666677 7777777788 8888888899 9999999900 0000000011 1111111122 2222222233 3333333344 4444444455 5555555566 6666666677 7774455555 5555566666 ]

[ 2345678901 2345678901 2345678901 2345678901 2345678901 2345678901 2345678901 2345678901 2345678901 2345678901 2345678901 2348901234 5678901234 ]

#19a ---------- ---------- ---------- ---------- ---------- ---------- ---------- ---------- ---------- ---------- ---------- ---------- ----------

#19b ---------- ---------- ---------- ---------- ---------- ---------- ---------- ---------- ---------- ---------- ---------- ---------- ----------

#19c ---------- ---------- ---------- ---------- ---------- ---------- ---------- ---------- ---------- ---------- ---------- ---CTTGTTC GGTAACTGTA

#19d ---------- ---------- ---------- ---------- ---------- ---------- ---------- ---------- ---------- ---------- ---------- ---------- ----------

#19e ---------- ---------- ---------- ---------- ---------- ---------- ---------- ---------- ---------- ---------- ---------- ---------- ----------

#19f NNNNNNNNNN NNNNNNNNNN NNNNNNNNNN NNNNNNNNNN NNNNNNNNNN NNNNNNNNNN NNNNNNNNNN NNNNNNNNNN NNNNCTTTTT ACAGTTACCG AACAAGTCTA TTA------- ----------

#19g ---------- ---------- ---------- ---------- ---------- ---------- ---------- ---------- ---------- ---------- ---------- ---------- ----------

#19h ---------- ---------- ---------- ---------- ---------- ---------- ---------- ---------- ---------- ---------- ---------- ---------- ----------

#19i ---------- ---------- ---------- ---------- ---------- ---------- ---------- ---------- ---------- ---------- ---------- ---------- ----------

#19j ---------- ---------- ---------- ---------- ---------- ---------- ---------- ---------- ---------- ---------- ---------- ---------- ----------

#19k ---------- ---------- ---------- ---------- ---------- ---------- ---------- ---------- ---------- ---------- ---------- ---------- ----------

#19l ---------- ---------- ---------- ---------- ---------- ---------- ---------- ---------- ---------- ---------- ---------- ---------- ----------

#19m ---------- ---------- ---------- ---------- ---------- ---------- ---------- ---------- ---------- ---------- ---------- ---------- ----------

[ 1111111111 1111111111 1111111111 1111111111 1111111111 1111111111 1111111111 1111111111 1111111111 1111111111 1111111111 1111111111 1111111111 ]

[ 3333333333 3333333333 3333333333 3333333333 3333333333 3333333333 3333333333 3333333333 3333333333 3333333333 3333333333 3333333333 3333333333 ]

[ 2222222222 2222222222 2222222222 2222233333 3333333333 3333333333 3333333333 3333333333 3333333333 3333333333 3333333333 3333333333 3333333333 ]

[ 6666677777 7777788888 8888899999 9999900000 0000011111 1111122222 2222233333 3333344444 4444455555 5555566666 6666677777 7777788888 8888899999 ]

[ 5678901234 5678901234 5678901234 5678901234 5678901234 5678901234 5678901234 5678901234 5678901234 5678901234 5678901234 5678901234 5678901234 ]

#19a ---------- ---------- ---------- ---------- ---------- ---------- ---------- ---------- ---------- ---------- ---------- ---------- ----------

#19b ---------- ---------- ---------- ---------- ---------- ---------- ---------- ---------- ---------- ---------- ---------- ---------- ----------

#19c AAAAGNNNNN NNNNNNNNNN NNNNNNNNNN NNNNNNNNNN NNNNNNNNNN NNNNNNNNNN NNNNNNNNNN NNNNNNNNNN NNNNNNNNNN NNNNNNNNNN NNNNNNNNNN NNNNNNNNNN NNNNNNNNNN

#19d ---------- ---------- ---------- ---------- ---------- ---------- ---------- ---------- ---------- ---------- ---------- ---------- ----------

#19e ---------- ---------- ---------- ---------- ---------- ---------- ---------- ---------- ---------- ---------- ---------- ---------- ----------

#19f ---------- ---------- ---------- ---------- ---------- ---------- ---------- ---------- ---------- ---------- ---------- ---------- ----------

#19g ---------- ---------- ---------- ---------- ---------- ---------- ---------- ---------- ---------- ---------- ---------- ---------- ----------

#19h ---------- ---------- ---------- ---------- ---------- ---------- ---------- ---------- ---------- ---------- ---------- ---------- ----------

#19i ---------- ---------- ---------- ---------- ---------- ---------- ---------- ---------- ---------- ---------- ---------- ---------- ----------

#19j ---------- ---------- ---------- ---------- ---------- ---------- ---------- ---------- ---------- ---------- ---------- ---------- ----------

#19k ---------- ---------- ---------- ---------- ---------- ---------- ---------- ---------- ---------- ---------- ---------- ---------- ----------

#19l ---------- ---------- ---------- ---------- ---------- ---------- ---------- ---------- ---------- ---------- ---------- ---------- ----------

#19m ---------- ---------- ---------- ---------- ---------- ---------- ---------- ---------- ---------- ---------- ---------- ---------- ----------

[...IS...]

[ 1111111111 1111111111 1111111111 1111111111 1111111111 1111111111 1111111111 1111111111 1111111111 1111111111 1111111111 1111111111 1111111111 ]

[ 4444444444 4444444444 4444444444 4444444444 4444444444 4444444444 4444444444 4444444778 8888888888 8888888888 8888888888 8888888888 8888888888 ]

[ 0000000000 0000000000 0000000000 0000000000 0000000000 0000011111 1111111111 1111111442 4444444444 4444444444 4444444444 4444444455 5555555555 ]

[ 4444455555 5555566666 6666677777 7777788888 8888899999 9999900000 0000011111 1111122557 6666666677 7777777788 8888888899 9999999900 0000000011 ]

[ 5678901234 5678901234 5678901234 5678901234 5678901234 5678901234 5678901234 5678901788 2345678901 2345678901 2345678901 2345678901 2345678901 ]

#19a ---------- ---------- ---------- ---------- ---------- ---------- ---------- -------A-C TACTCAATGA AAATCAAAGA GCAAACTAGG AAACTAGCCG CAGGCTGTAC

#19b ---------- ---------- ---------- ---------- ---------- ---------- ---------- -------.-. .......... .......... .......... .......... ..........

#19c NNNNNNNNNN NNNNNNNNNN NNNNNNNNNN NNNNNNNNNN NNNNNNAGTG AACTAGATTC CGAACAAGTC TAATAGA.-. ---------- ---------- ---------- ---------- ----------

#19d ---------- ---------- ---------- ---------- ---------- ---------- ---------- -------.-. .......... .......... .......... .......... ..........

#19e ---------- ---------- ---------- ---------- ---------- ---------- ---------- -------.-. .......... .......... .......... .......... ..........

#19f ---------- ---------- ---------- ---------- ---------- ---------- ---------- -------.A. ---------- ---------- ---------- ---------- ----------

#19g ---------- ---------- ---------- ---------- ---------- ---------- ---------- -------.A. ---------- ---------- ---------- ---------- ----------

#19h ---------- ---------- ---------- ---------- ---------- ---------- ---------- ---------. .......... .......... .......... .......... ..........

#19i ---------- ---------- ---------- ---------- ---------- ---------- ---------- ---------. .......... .......... .......... .......... ..........

#19j ---------- ---------- ---------- ---------- ---------- ---------- ---------- ---------. .......... .......... .......... .......... ..........

#19k ---------- ---------- ---------- ---------- ---------- ---------- ---------- -------.-. .......... .......... .......... .......... ..........

#19l ---------- ---------- ---------- ---------- ---------- ---------- ---------- -------.A. .......... .......... .......... .......... ..........

#19m ---------- ---------- ---------- ---------- ---------- ---------- ---------- -------.-- .......... .......... .......... .......... ..........

[ 1111111111 1111111111 1111111111 1111111111 1111111111 1111111111 1111111111 1111111111 1111111111 1111111111 1111111111 1111111111 1111111111 ]

[ 8888888888 8888888888 8888888888 8888888888 8888888888 8888888888 8888888888 8888888888 8888888888 8888888888 8888888888 8888888888 8888888888 ]

[ 5555555555 5555555555 5555555555 5555555555 5555555555 5555556666 6666666666 6666666666 6666666666 6666666666 7777777777 7777777777 7777777777 ]

[ 1111111122 2222222233 3333333344 4444444455 5555555566 6666661555 6666666666 7777777777 8888888888 9999999999 0000000000 1111111111 2222222222 ]

[ 2345678901 2345678901 2345678901 2345678901 2345678901 2345673789 0123456789 0123456789 0123456789 0123456789 0123456789 0123456789 0123456789 ]

#19a TTGAGTACGG CAAGGCGACG TTGACGTGGT TTGAATTTGA TTTTCGAAGA GTATAA---- ---------- ---------- ---------- ---------- ---------- ---------- ----------

#19b .......... .......... .......... .......... .......... ......---- ---------- ---------- ---------- ---------- ---------- ---------- ----------

#19c ---------- ---------- ---------- ---------- ---------- ---------- ---------- ---------- ---------- ---------- ---------- ---------- ----------

#19d .......... .......... .......... .......... .......... ......G--- ---------- ---------- ---------- ---------- ---------- ---------- ----------

#19e .......... .......... .......... .......... .......... ......G--- ---------- ---------- ---------- ---------- ---------- ---------- ----------

#19f ---------- ---------- ---------- ---------- ---------- ---------- ---------- ---------- ---------- ---------- ---------- ---------- ----------

#19g ---------- ---------- ---------- ---------- ---------- ---------- ---------- ---------- ---------- ---------- ---------- ---------- ----------

#19h .......... .......... .......... .......... .......... ......-CCC CAATTATTCA CCCCAAATCT AANNNNNNNN NNNNNNNNNN NNNNNNNNNN NNNNNNNNNN NNNNNNNNNN

#19i .......... .......... .......... .......... .......... ......---- ---------- ---------- ---------- ---------- ---------- ---------- ----------

#19j .......... .......... .......... .......... .......... ......---- ---------- ---------- ---------- ---------- ---------- ---------- ----------

#19k .......... .......... .......... .......... .......... ......---- ---------- ---------- ---------- ---------- ---------- ---------- ----------

#19l .......... .......... .......... .......... .......... ......---- ---------- ---------- ---------- ---------- ---------- ---------- ----------

#19m .......... .....A.... .......... .......... .......... ......---- ---------- ---------- ---------- ---------- ---------- ---------- ----------

[...IS...]

[ 2222222222 2222222222 2222222222 2222222222 2222222222 2222222222 2222222222 22222]

[ 0000000000 0000000000 0000000000 0000000000 0000000000 0000000000 0000000000 00000]

[ 2222222222 3333333333 3333333333 3333333333 3333333333 3333333333 3333333333 33333]

[ 9999999999 0000000000 1111111111 2222222222 3333333333 4444444444 5555555555 66666]

[ 0123456789 0123456789 0123456789 0123456789 0123456789 0123456789 0123456789 01234]

#19a ---------- ---------- ---------- ---------- ---------- ---------- ---------- -----

#19b ---------- ---------- ---------- ---------- ---------- ---------- ---------- -----

#19c ---------- ---------- ---------- ---------- ---------- ---------- ---------- -----

#19d ---------- ---------- ---------- ---------- ---------- ---------- ---------- -----

#19e ---------- ---------- ---------- ---------- ---------- ---------- ---------- -----

#19f ---------- ---------- ---------- ---------- ---------- ---------- ---------- -----

#19g ---------- ---------- ---------- ---------- ---------- ---------- ---------- -----

#19h NNNNNNNNNN NNNNNNNNNN NNNNNNNNNN NNNNNNNNNN NNNNNTTGAG AGAGGTATGA ATGATTTGGG TTGAT

#19i ---------- ---------- ---------- ---------- ---------- ---------- ---------- -----

#19j ---------- ---------- ---------- ---------- ---------- ---------- ---------- -----

#19k ---------- ---------- ---------- ---------- ---------- ---------- ---------- -----

#19l ---------- ---------- ---------- ---------- ---------- ---------- ---------- -----

#19m ---------- ---------- ---------- ---------- ---------- ---------- ---------- -----

**Category 23**

**Variable nucleotides**

[ 111111 1111111111 1111111111 1111111111 2222333333 3333333444 4444444444 4555555555 5555555666 6666666666 6666677777 7789999999 ]

[ 1114566778 9999113444 5555555566 6777777777 7777778889 0457444555 5677788023 5555556899 9000444444 4457888122 2467788899 9999900000 0033444444 ]

[ 5560335230 3334882034 0112357823 8012234444 4678892336 6095488666 8234408733 0077899303 4236111222 6677389434 6726746803 4567700122 3375023455 ]

[ 4692105197 0466354282 2465420519 8711540256 9023730466 6519812458 0412556176 0702847294 9453245039 5769777535 3567359650 3581746689 8907880023 ]

#23a TGATCGGGCG ACAAAATTGA CCCGCTCATG GGCGTGCCAT AAAGCTTCGG CATCGACAGG TCCCTTATAT TCTAGTATCA TAAGGGGGCC ACCCAGGCAC GCCGAGCGAG TCCGACATAA TATGTCATAA

#23b CATCTAAAGA GGGGTGCCAC TTTATCAGAC AATACAATGC GTGATCGTTC TGGTATGGAT ATTTCAGCGC CTCGACGCAG CGCATAAATT GTTTGTATGT ATTAGAATGA CATCGATCTT CGCACATCTT

[ 11]

[ 9999999999 922]

[ 4444444444 558]

[ 5666668889 041]

[ 8023475690 203]

#23a TGTACTGTGT TTT

#23b AACGAAAGAC AGC

**Nucleotides with coverage <100%**

[ 555555555 5555555555 5555555555 5555555555 5555555555 5555555555 5555555555 5555555556 6666666666 6666666666 6666666666 6999999999 9999999999 ]

[ 5999999999 9999999999 9999999999 9999999999 9999999999 9999999999 9999999999 9999999991 1111111111 1111111111 1111111999 9333333333 3333333333 ]

[ 1000000000 0111111111 1222222222 2333333333 3444444444 4555555555 5666666666 6777777771 1111112222 2222223333 3333334255 7444455555 6666666666 ]

[ 1012345678 9012345678 9012345678 9012345678 9012345678 9012345678 9012345678 9012345673 4567890123 4567890123 4567890801 6678901234 0123456789 ]

#23a AGAAGAGATT AGGGTTTTGT GCCAGTAAGA CGCCCCCTAC AAGAATCAGA ATAGAAACAT CCAGAAAAAG AGAAAGAATT AATCTCTGGC TCAGAACGGT TAGGAGAG-- AAGTAAAAAA TAGAAAACAT

#23b ---------- ---------- ---------- ---------- ---------- ---------- ---------- ---------- ---------- ---------- --------TA ---------- ----------

[ 9999999999 9999999999 9999999999 999]

[ 3333333333 3333333333 3333344444 444]

[ 7777777777 8888888888 9999900000 000]

[ 0123456789 0123456789 0678901234 567]

#23a CTTTTTTAAA AATAGAGATG AAAACAAAAA AGC

#23b ---------- ---------- ---------- ---

**Category 24**

**Variable nucleotides**

[ 111111111 1111111111 1111111111 1111111111 1111111111 1111111111 1111111111 1111111111 ]

[ 11122222 2455556667 7778999999 9000000001 1111112222 2233333344 4444555555 5555666666 6667777777 7777777777 7777778888 ]

[ 1111 1111222223 7855812334 9035671352 3780133347 9122346884 5888894678 8912446903 4444011223 4578023367 7890012223 3444446678 8889991222 ]

[ 4567890134 5678024891 3246466788 8218313051 9007504660 4846829474 6356923536 8124080628 1248246584 6205313992 8876711584 8025690922 3783698034 ]

#24a CAAGATAAGG TTGGCGAACC TACGCCTCAG GCTTGCCAAA CGTGAGGGAG GCGACCAAGG GAAACCCCTC TGATATGCTA CCACTTCACC TTAGCTGGTT CGGCACGTGG TATACGTAAG GCACTACGAT

#24b GTTTGCTCTT GATTTATTAT CG.ATTC.GC A..G...... G.CAT...G. .......... .TG....... ...C....C. ......T.TT .C..GAAC.. .A.T.TACAA C..G...GGA AT..GGT.GA

#24c GTTTGCTCTT GATTTATTAT CGT.TTC.GC AT....TG.G ..C.T...G. ATACTTGTAA ATGGTTTTCT GAGC.....G G.CG...G.. C...G.ACCC A.A.....A. C.....CGG. ATG.GGTTGA

#24d GTTTGCTCTT GATTTATTAT CG.ATTCGGC A...A..G.G ..C.T...G. ATACTTGTAA ATGGTTTTCT GAGC...... G.CG...G.. C...G.ACCC A.A.....A. C.....CGG. AT..GGT.GA

#24e GTTTGCTCTT GATTTATTAT CGT.TTC.GC A.....TG.G ..C.T...GA ATACTTG..A ATGGTG.... ..TCGCATCG .A..CC.GT. ..CAG.A... ...TG...A. CCC.TAAG.A ...TGGTTGA

#24f GTTTGCTCTT GATTTATTAT CGT.TTC.GC ATCG.A.GGG .AC.TACA.. .......... .......... .........G .A..CC.GT. ..CAG.A... ...TG...A. CCC.TAAG.A ...TGGTTGA

[ 1111111111 1111111111 1111111111 1111111111 1111111111 1122222222 2222222222 2222222222 2222222222 2222222222 2222222222 2222222222 2222222222 ]

[ 8888888888 8888888888 8888888888 8888999999 9999999999 9900000000 0000000000 0000000000 0000000111 1111111111 1111111222 2222222222 2222222223 ]

[ 2233333334 4445555556 6666777777 8899001112 3335556788 8900034444 4455555566 6666667777 7888999000 0112334555 6677889001 1112222233 4445556790 ]

[ 6902456895 6790145680 2457023478 0328373468 1670686358 9106961234 5934678912 3456890127 8147125234 6016284257 2701360230 2360258937 2345891270 ]

#24a TTCAGGACAA CGCTTCAATT GCTTGAAATT GCATTTTCAG AGGAATGCAA GGCTTGAAAT TGGGATGGAA TCCTTCTTTT GTGAGTGAGT TTCATCAGAT ACGTTTAGCG CTTCAGGCGA ACCAAGTCGT

#24b AGTGTCTTGG TATGAAGCAA ATAGAGTTCC ATGCACCTG. ...CTCCTGG ATTACAGGCA ATATGCTATC ATT.CTGGGG TAATAATTTC CC.G.TT..A ....CG.AT. ..CTG.A..G ..TG.....C

#24c AGTGCCTTGG T.TGAAGCAA ATAGAGTTCC ATGCACCT.A G..CTC.TGG ATTACAGGCA ATATGCTATC ATT.CTGGGG TAATAATTTC CCTGCTCA.A CACCCGCATT GCC..C.G.G TTT..A.AC.

#24d AGTGCCTTGG T.TGAAGCAA ATAGAGTTCC ATGCACCT.A G..CTC.TGG ATTACAGGCA ATATGCTATC ATT.CTGGGG TAATAATTTC CCTGCTCA.A CACCCGCATT GCC..C.G.G TTT..A.AC.

#24e AGTGCCGTGG GATGCGGCAA .TAGAGTTCC ATGCACCT.- .AACCC.T.G ATT..AGGCA ATAT.CTATC ATT..T.GGG TAATAATTTC CCTG..CAGA CAA.CGCAT. ..CTG.A.AG ..T.G.G.C.

#24f AGTGCCGTGG TATGAAGCAA ATAGAGTTCC ATGCACCTG. ...CTC.TGG ATTACAGGCA ATATGCTATC ATTCCTGGGG TAATAATTTC CC.G.TT..A ....CG.AT. ..CTG.A..G ..TG.....C

[ 2222222222 2222222222 2222222222 2222222222 2222222222 2222222222 2222222222 2222222222 2222222222 2222222222 3333333333 3333333333 3333333333 ]

[ 3333333333 3333333344 4444444444 4444455555 5555555566 6666666666 6666677777 7777777788 8888999999 9999999999 0000000000 0000000000 1111111111 ]

[ 0022566666 6777888900 3344455667 8889933344 5566799900 1111123344 5668902235 6778888802 3445002345 6667778899 0012233344 5667778999 0000111122 ]

[ 6923401367 8035568625 2857839581 0363827923 1824414537 2567873929 7374680399 2490356771 7062237051 3493791737 2574613423 1460141369 2357026701 ]

#24a ACGGATCGGG ACACACTCTG ACATCTCCCT ACCTCACACG GCCGGGCAGA TCCGTGCTAA TAATGTCAAC TTCAGCCACA TTTCCCATTG TCCAACATCA ACAGGAGCTA CTTTTCGCAA TATCGGTTGA

#24b CA.TCC.TTA G.G.GT.T.. ..GAA....C GATCTGT.T. ATAA...C.G GT...A.... ..GCA.AG.T .G.....GTG CCCTTTTC.. .T.GGT..TG .......... .......... ..........

#24c ...T.CTT.A GTGAG.CT.. G.GA...TTC GT.C.GTG.C A...A...A. .TAAAATAGT CT.C.CT... .CTGA.T... C.C.....C. C.T.G..CT. ......A..G TA....ATTT ATAG.TA...

#24d ...T.CTT.A GTGAG.CT.. G.GA.CTTTC GT.C.GTG.C A...A...A. .TAAAATAGT CT.C.CT... .CTGAGT... C.C.....C. C.T.G..CT. ......A..G TA....ATTT ATAG.TA...

#24e G.ATCC.T.A G...GA..G. GTGA.....C GT.C...... ATGA.TTC.. .TAAAA.... ......T.G. C.T.A..TT. C..T.A...A ....G.CC.G CT..A.ATAG TGGC.T.TTT CTAGAC..AT

#24f CA.TCC.TTA G.G.GT.T.A ..GAA....C GATCTGT.T. ATAA.T.C.G GT...A.... ..GCA.AG.. .G.....GTG CCCTTTTC.. .T.GGT..TG ..GA.CA..G T...A.ATTT ATAG.C.CAT

[ 3333333333 3333333333 3333333333 3333333333 3333333333 3333333333 3444444444 4444444444 4444444444 4444444444 4455555555 5555555555 5566666666 ]

[ 1111111111 1111111111 1111111334 4445555555 5667777777 7788888899 9000000001 1111222344 4444555555 5667778889 9900000112 2445568899 9911222244 ]

[ 2222223333 3334444444 4555667151 4880026668 8271123334 4901123638 9012667893 4668006812 7899112288 9040183394 4534579140 1074755934 4723457825 ]

[ 3567890134 6791234567 9356678150 9236705691 3563582473 6105843953 9425175109 2297185284 8702073609 8467622994 7945637221 7953294351 5413464613 ]

#24a TTAAATTTCT AAATGTTTCA GGGTCAGGCA GTGAACGATA CTCACGTTGT CGGTGCCGAA CTCCTGTCAA AAAGTCTTCT TGGCCTGATA ACGACGCAGG TTAACGAGTG TAAGATCATG CGGGGTATGG

#24b .......... .......... .......... .......... .......... .......... .......... .......... .......... .......... .......... .......... ..........

#24c GA.TGAGCTA CTTAA.G.T. ATAAA..TTG T..GG..... AC.GA..CA. GAA....... .......... .......... .......... .......... .......... ...A...... ..........

#24d GA.TGAGCTA CTTAA.G.T. ATAAA..... .....T.... ..A...C..C TA.AATTA.C TATTA...GG GCGAAGCCAC CATTTCCTCC GTATTATGAA GCGCTAGAG. C.G..C.GCT TAAAACG.TT

#24e ..TTG..CA. .TTAAA.CT. A.AA..A.TG .ACGG.AGGT AC...C.... .A.AATTA.C TATTA...GG GCGAAGCCAC CATTTCCTCC GTATTATGAA GCGCTAGAG. C.G..C.GCT TAAAACG.TT

#24f G..TGC..A. .TTAAGGCTG A.TAAG.... .ACGG.AGGT AC....C..C TA...T.ACC TATTATCTGG GT........ .......... .......G.. .........A .GGAGCTGCT .A.....G..

[ 111 1111111111 1111111111 1111111111 1111111111 1111111111 1111111111 1111111111 1111111111 1111111111 1111111111 ]

[ 6666667777 7777777777 7777899000 0000111111 1111111111 1111111111 1111111222 2222222222 2222222222 2222222222 2222222222 2222222222 2222222222 ]

[ 5666880000 0000000011 1299567125 5556115566 6666666666 6777777777 7888999000 0001111111 1111111111 1111111111 1122222222 2222222222 2222222222 ]

[ 1024671224 4556779903 3812566631 2441292900 0112244458 9125567789 9034022446 6772333333 3445566666 7777888899 9900000001 1111112223 3344455566 ]

[ 4701867091 2129485618 9635135981 2688665801 6693537885 5626733910 3524889285 9183234678 9053413469 2359124703 7801257891 2345670792 5902713448 ]

#24a CTACACGATA ATAGAGTATT CGCGCGTAGT GGAGCTATTA TGAGGTTAAC TGGTCTCCTT GAGCCAAAAA CGCCAGTTCA ACATAGCTCT CTATCGATTT CACTGGTTAC GTCCAACCAT TCAACTATAT

#24b .......... ......-T.. .A........ ...AAC.... .......... .......T.. .......... .......... G..C.AT.AC .C........ .......... .......... ..........

#24c .......... T.....-T.. ..T...CG.. ....A..... .......... .......... .......... .......... .......... .......... .......... .......... ..........

#24d T.CTCTA.CT TCGA.AA.GG A..T.ACGA. ....A.G... .......... .......... .......... .......... .......... .......... .......... .......... ..........

#24e T.CTCTA.CT TCGA.AA.GG A..T.ACGA. ....A.G... .......... .......... .......... .......... .......... .......... .......... .......... ..........

#24f TCC...AG.. ....G....G A...T.CG.C ATT.A..GCT CACTTCCCGT CAAATGA.CA ACAATGGGGG TAAAGACCGC TATCGATCT. T.GCGTTCAG TGACACAATT TGGAGCTGGC GTGGAATAGC

[ 1111111111 1111111111 1111111111 1111111111 1111111111 1111111111 1111111111 1111111111 1111111111 1111111111 1111111111 1111111111 1111111111 ]

[ 2222222222 2222222222 2222222222 2222222222 2222222222 2222222222 2222222222 2222222222 2222222222 2222222222 2222222222 2222222222 2222222222 ]

[ 2222222222 2333333333 3333333333 3333333333 3333444444 4444444444 4444444444 4555555555 5555555555 5555555555 6666666666 6666666666 6666666666 ]

[ 7778889999 9000011111 2344445566 6777788899 9999000111 3333444555 5666678899 9000011112 2222444555 5678899999 0000111122 2333444455 5667777888 ]

[ 2480390235 9235803579 7703695912 4014927801 4579056347 0378345156 8038971404 6056924580 3569579367 9211712578 2478367928 9147236728 9070579035 ]

#24a TGTATTGAGC AGTGCTTAAA GCCTAAACTT GGATAATGTA GTAAGCTGTA TTTCGCGTGC GAGGTGTCAC AATAGCGTTT GTTAATAAAT AGTCCTTTTT TGTCTTATAT CCTTCACCTG TTCCTGAAGT

#24b .......... .......... .......... .......... ..C....... .......... .......... .......... .........C .......... .......... .......... ..........

#24c .......... .......... .......... .......... .......... .......... .......... .......... .......... .......C.. .....C..G. .T..TGT... ........T.

#24d .......... .......... .......... .......... ..C....... .......... .......... .......... .......... .......... .......... .......... ..........

#24e .......... .......... .......... .......... ..C....... .......... .......... .......... .......... .......... .......... .......... ..........

#24f ACCTCCTCTG GTGATGCGTG CTTCTGGTGA CTGCGGCCCT TG.TTGCTAT CCGGAATGAA TGTCCAATGT TGCCATAAGA AGATGAGGCC GTCGAGGGAG CTCTGATC.C TGCC.T.TAC GCTTAAGG.G

[ 1111111111 1111111111 1111111111 1111111111 1111111111 1111111111 1111111111 ]

[ 2222222222 2222222222 2222222222 2222222222 2223333333 3333333333 3333333333 ]

[ 6666667777 7777777777 7777777777 7888888899 9990122222 3333444555 5556677788 ]

[ 8899990000 0112233444 4555666677 7223589901 4885923399 3567458233 3880715811 ]

[ 8901240146 9284736035 9124346915 8166251768 2196235616 1204422803 6482762226 ]

#24a AATCACTGTG CCCCTACGGG GGACTGAGCC GAGCCCTTAA TTTGTTATAT GCCTCGATAG CAACGTTTTC

#24b .......... .......... .......... .......... ...AA..... .......... ...T......

#24c ......C... ...T...... .......... .......... ....A..... A.A....... ..........

#24d .......... .......... .......... .......... .......... .......... ..........

#24e .......... .......... .......... .......... .......... .......... ..........

#24f CTGTCGCTCA ATGTCTTTAT ATGTCACAAA ACCTTACCGG CCCAAGGCCC AAAAGAGGGA TGTAACCCCT

**Nucleotides with coverage <100%**

[ 1111111111 1111111111 1111111111 1111111111 1111111111 1111111111 1111111111 1111111111 1111111111 ]

[ 11111111 1113333333 3333333566 6667777899 0112222222 2222222333 3333333333 3333333333 3333333333 3333333333 3333333333 3333333333 3333333333 ]

[ 4599999999 9990000077 7777777633 3390000477 4220000000 0111111000 0000000000 0000000000 0000000000 0000000001 1111111111 1111111111 1111111111 ]

[ 1111222222 2224468866 6677777466 6754499278 5553999999 9000000556 6666666667 7777777778 8888888889 9999999990 0000000001 1111111112 2222222223 ]

[ 2189012345 6784582367 8901234478 9074545484 0237123456 7123456890 1234567890 1234567890 1234567890 1234567890 1234567890 1234567890 1234567890 ]

#24a --CTTTCTTA TTG--G---- ---------- --TTA-TAAA -TAA------ ---------- ---------- ---------- ---------- ---------- ---------- ---------- ----------

#24b --........ ...--.---- ---------- --...--.-. C..------- -------TAA TACTCAATGA AAATCAAAGA GCAAACTAGG AAACTAGCCG CAGGCTGTAC TTGAGTACGG CAAGGCGACG

#24c -A........ ..A--.TT-- ---------- --------.- -..------- ---------- ---------- ---------- ---------- ---------- ---------- ---------- ----------

#24d --........ ..A--.TTTT AATTCTGCGC TT...AA... -...------ ---------- ---------- ---------- ---------- ---------- ---------- ---------- ----------

#24e T--------- ----A-TT-- -------CGC TT-..-A... -...------ ---------- ---------- ---------- ---------- ---------- ---------- ---------- ----------

#24f TA........ ...AA.-T-- ---------- ---..-.... ---.CTATCC TTTGTAGTAA TACTCAATGA AAATCAAAGA GCAAACTAGG AAACTAGCCG CAGGCTGTAC TTGAGTACGG CAAGGCGACG

[ 1111111111 1111111111 1111111111 111]

[ 3333333333 3333333333 3333333333 333]

[ 1111111111 1111111111 1111111111 111]

[ 3333333334 4444444445 5555555556 666]

[ 1234567890 1234567890 1234567890 123]

#24a ---------- ---------- ---------- ---

#24b TTGACGTGGT TTGAATTTGA TTTTCGAAGA GTA

#24c ---------- ---------- ---------- ---

#24d ---------- ---------- ---------- ---

#24e ---------- ---------- ---------- ---

#24f TTGACGTGGT TTGAATTTGA TTTTCGAAGA GTA

**Category 25**

**Variable nucleotides**

[ 1 1111111111 1111111111 1111111111 1111111111 1111111111 1111111111 ]

[ 111222233 3333333444 4444445555 5555667777 7778888990 0000000000 0011111111 2222222333 3444444455 5555556667 7777777777 ]

[ 1111 1111122222 3558346913 3466799001 1122280234 6677560022 4781268131 2234445567 7745688899 0036677446 9013478901 1223780230 0234445678 ]

[ 4567890134 5678902489 1469588832 7445026892 8901585124 5723653724 0111420679 5792365840 3757646703 2582614191 7659387635 7693164847 8991370103 ]

#25a GTTTGCTCTT GATTCTATTA TTGCGCGAGG GTACGTTATT TTTTCTGCTA GCCCAACCAA CGTTTCTAGT ACTCTCGACG CCAAAAAACT ACTATCCATG CACACGAGTT TACCAGCCGC AGTATCGTAG

#25b .......... .......... ...T...... .......... .......... .......... .......... .......... .......... .......... .......... .......... ..........

#25c CAGGATAAGG TTGGTCGAGC CCA.AGACAA TCGAACCTCC CGCCTGATCG TTTTGGTAGG GACCGTCTAC GACTGTATTA TTGTGTGGTG GTCTC.TGCA TGTGAAGACC CGT.CAGTAT GACCCTAAGA

#25d .......... .......... .CA..GACAA TCGAACCTCC CGCCTGATCG TTTTGGTAGG GACCGTCTAC GACTGTATTA TTGTGTGGTG GTCTCTTGCA TGTGAAGACC CGTTCAGTAT GACCCTAAGA

[ 1111111111 1111111111 1111111111 1111111111 1111111111 1111112222 2222222222 2222222222 2222222222 2222222222 2222222222 2222222222 2222222222 ]

[ 7788888888 8888888888 8888888888 8888888888 8999999999 9999990000 0000000000 0000000000 0000000000 1111111111 1111111122 2222222222 2333333333 ]

[ 9901222233 3333344445 5555556666 6777777889 9001113355 5788990013 4444555555 6666666677 7777888999 0000112345 5566788900 1222334556 9001225666 ]

[ 4709145701 3567906780 1256791356 8134589143 9484577817 9469021707 3456045789 0234567901 2389258236 3457127953 6838147134 7139485692 8170345147 ]

#25a CTACGATTTC AGGACAACGC TTCAATTGCT TGAAATTGCA TTTTCAGGAA TCAAGGCTTG AATTGGGATG GAATCCTTCT TTTGTGAGTG AGTTTCACAG ATACGTTAGC TCAGGACAAT GTACGGATGG

#25b .......... .......... .......... .......... .......... .......... .......... .......... .......... .......... .......... .......... ..........

#25c TGGTTGAAGT GCCGTGGTAT GAAGCAAATA GAGTTCCATG CACCTG..CT CTGGATTACA GCAATATGCT ATCATTCCTG GGGTAATAAT TTCCC.GTT. .A...CG.AT CTGA.GTG.. .CCA.TCCTT

#25d TGGTTGAAGT GCCGTGGGAT GCGGCAA.TA GAGTTCCATG CACCT.AACC CT.GATT..A GCAATAT.CT ATCATT..T. GGGTAATAAT TTCCCTG.CA GACAACGCAT CTGAAGT.GG C.G.ATCCT.

[ 2222222222 2222222222 2222222222 2222222222 2222222222 2222222222 2222222222 3333333333 3333333333 3333333333 3333333333 3333333333 3333333333 ]

[ 3333334444 4444444444 4555555555 6666666667 7777777778 8888888999 9999999999 0000000000 0000000011 1111111111 1111111111 1111111111 3444555555 ]

[ 6678890001 3344478889 9334556699 0112668990 1224678880 1234457002 3567788899 0012233345 6677789900 0001112222 2233334444 4444555667 5188006678 ]

[ 8946773692 3968921474 9383293526 8368485479 6140350488 7281731348 1254802848 3685724532 5712524703 4681381247 8902480234 5678067789 6134786702 ]

#25a GAAACCTGAC ACATCTACCT CACCGCCGGA ATCGAATTGT ACAATTCGAC AATTTCCCAA TACAACATCA ACAGGAGCTC TTTTCGCAAT ATCGGTGATA AATTCAATGT TTCAGGTCAG CATGAAGATA

#25b .......... .......... .......... .......... .......... .......... .......... .......... .......... .......... .......... .......... ..........

#25c AGGGTT.A.. ..GAACGATC TGTTATAATC GGTA.GC.A. .AG..G..GT .GCCCT.TTT CGTGGT..TG ..GA.CA..T ...A.ATTTA TAG.CCATG. TGC.ATTAAG GCTGATAAG. ..ACGGAGGT

#25d AG.GA.G.GG GTGA.CGT.C ....ATGATC GGTAT.CA.A GT.GC.TATT T.C..TA... ....G.CC.G CT..A.ATAT GGC.T.TTTC TAAAC.AT.T TG.CATTAAA .CT.AAA..A TGACGGAGGT

[ 11111]

[ 3333333333 3344444444 4444444444 4444444444 4444444444 4455555555 5555555556 6666666666 6667777777 777901112]

[ 5677888889 9900000111 1112223333 3445555555 5556678889 9900000011 2358889990 1112222446 6880000000 011607890]

[ 8228012562 3901348335 7795690235 7480001178 9993902391 5623446909 2373892332 1253467141 3562344557 844552562]

[ 4693705167 2647202144 6937868874 4942495821 0368984411 1767285543 3789996252 2445157241 2974689896 545000684]

#25a CTGGAATCTA CTCCTTGAAC CATTCGAAAT CACCACGACC GTTGGCCTGA TCAATTGGAG TGCAGGCTCC GGGACGTTTC TCTACTTCGA ATCAGAACA

#25b .......... .......... .......... .......... .......... .......... .......... .......... .......... .....GTTC

#25c ACAATGCTC. TATTCCAGG. TG.C.A.GGG .TTTT.CT.. ...ATTTCA. CGGCC.A... CATGTATGTT AA..T..... .......... .........

#25d ACAATGCTCC .ATTCCAGGT T.ACT.GGGG T....T..TA AGCA....AC ..G.CC.AGA C..G..TG.. ..AGTAGGGA CACCTAATAG GGAGA....

**Nucleotides with coverage <100%**

[ 111111 1111111111 1111111111 1111111111 1111111111 1111111111 1111111111 1111111111 1111111111 1111111111 ]

[ 33333666 6666666666 6666666777 7777111111 1111111111 1111111111 1111111111 1111111111 1111111111 1111111111 1111111111 1111111111 1111111111 ]

[ 4500000333 3999999999 9999999000 0011777777 7777777777 7777777777 7777777777 7777777777 7777777777 7777777777 7777777777 7788888888 8888888888 ]

[ 2144688556 6666666677 7777777999 9900222222 2233333333 3344444444 4455555555 5566666666 6677777777 7788888888 8899999999 9900000000 0011111111 ]

[ 2245934890 1345678901 2345678567 8901234567 8901234567 8901234567 8901234567 8901234567 8901234567 8901234567 8901234567 8901234567 8901234567 ]

#25a A---G--GCT TTAGAGGTGG CTTGAAA--- ----TAATAC TCAATGAAAA TCAAAGAGCA AACTAGGAAA CTAGCCGCAG GCTGTACTTG AGTACGGCAA GGCGACGTTG ACGTGGTTTG AATTTGATTT

#25b .---.--... .......... .......--- ---------- ---------- ---------- ---------- ---------- ---------- ---------- ---------- ---------- ----------

#25c -AGA.-T... .......... .......--- ----...... .......... .......... .......... .......... .......... .......... .......... .......... ..........

#25d -AG--TT--- ---------- -------AAA AATT...... .......... .......... .......... .......... .......... .......... .......... .......... ..........

[ 1111111111]

[ 1111111111]

[ 8888888888]

[ 1122222222]

[ 8901234567]

#25a TCGAAGAGTA

#25b ----------

#25c ..........

#25d ..........
